# Kidney epithelial cells are active mechano-biological fluid pumps

**DOI:** 10.1038/s41467-022-29988-w

**Published:** 2022-04-28

**Authors:** Mohammad Ikbal Choudhury, Yizeng Li, Panagiotis Mistriotis, Ana Carina N. Vasconcelos, Eryn E. Dixon, Jing Yang, Morgan Benson, Debonil Maity, Rebecca Walker, Leigha Martin, Fatima Koroma, Feng Qian, Konstantinos Konstantopoulos, Owen M. Woodward, Sean X. Sun

**Affiliations:** 1 Department of Mechanical Engineering, Johns Hopkins University, Baltimore, MD United States; 2 Institute of NanoBioTechnology, Johns Hopkins University, Baltimore, MD United States; 3 Department of Mechanical Engineering, Kennesaw State University, Marietta, GA United States; 4 Department of Chemical and Biomolecular Engineering, Johns Hopkins University, Baltimore, MD United States; 5 Department of Chemical Engineering, Auburn University, Auburn, AL United States; 6 Department of Biochemistry and Molecular Biology, Maryland PKD Research and Clinical Core Center, University of Maryland School of Medicine, Baltimore, MD United States; 7 Department of Physiology, Maryland PKD Research and Clinical Core Center, University of Maryland School of Medicine, Baltimore, MD United States; 8 Maryland PKD Research and Clinical Core Center, University of Maryland School of Medicine, Baltimore, MD United States

**Keywords:** Permeation and transport, Morphogenesis, Tissues, Kidney

## Abstract

The role of mechanical forces driving kidney epithelial fluid transport and morphogenesis in kidney diseases is unclear. Here, using a microfluidic platform to recapitulate fluid transport activity of kidney cells, we report that renal epithelial cells can actively generate hydraulic pressure gradients across the epithelium. The fluidic flux declines with increasing hydraulic pressure until a stall pressure, in a manner similar to mechanical fluid pumps. For normal human kidney cells, the fluidic flux is from apical to basal, and the pressure is higher on the basal side. For human Autosomal Dominant Polycystic Kidney Disease cells, the fluidic flux is reversed from basal to apical. Molecular and proteomic studies reveal that renal epithelial cells are sensitive to hydraulic pressure gradients, changing gene expression profiles and spatial arrangements of ion exchangers and the cytoskeleton in different pressure conditions. These results implicate mechanical force and hydraulic pressure as important variables during kidney function and morphological change, and provide insights into pathophysiological mechanisms underlying the development and transduction of hydraulic pressure gradients.

## Abstract (web summary)

How mechanical forces drive fluid transport in the kidney remains unclear. Here, the authors use a microfluidic platform to show that kidney epithelial cells generate hydraulic pressure gradients across the epithelium, and that the fluid flux is from apical to basal for normal cells, and inverted in autosomal dominant polycystic kidney disease cells.

## Introduction

Many organs are made of a series of tubules lined with epithelial cells. For the human kidney, roughly one million nephrons with 30 kilometers of epithelial tubules re-absorb 180 L of water per day^1^. While the absorption activity of renal epithelial cells has been studied both in vitro and in vivo^2–4^, the influence of forces and hydraulic pressures during absorption has not been examined, mainly due to difficulty in controlling these variables during experimentation. Mechanical forces are recognized as important elements during cell growth, differentiation, and tissue morphogenesis^5–7^. For kidney disorders such as the Autosomal Dominant Polycystic Kidney Disease (ADPKD), where tubular morphology of the epithelium becomes disrupted and uncontrolled expansion of the cyst results, mutations in the polycystin genes (*PKD1* and *PKD2*) and their protein products (PC1 and PC2) could also alter the shape and mechanical state of the kidney epithelium^8,9^. Several groups have investigated the mechanism of a fluid-driven epithelial morphogenesis in models such as embryos^10,11^, vasculature^12^, and during lumen formation^13^. However, an understanding of the interplay between fluid flow and the hydraulic pressure gradient in driving tissue shape change in the kidney tubular epithelial cells is missing. This is due to lack of tools that can simultaneously measure trans-epithelial fluid flux and hydraulic pressure gradient across epithelial monolayers in a physiologically relevant setup. Additionally, while numerous studies have investigated the role of mechanosensitive ion-channels in sensing absolute hydraulic pressure experienced by epithelial cells^14–17^, it is unclear how polarized epithelia generate, sense, and transduce hydraulic pressure gradients into downstream molecular events. Moreover, in some epithelial monolayer models^18^, it is hard to decouple pressure and mechanical stretch, making it difficult to draw clear conclusions regarding how hydraulic pressure gradients influence trans-epithelial fluid transport.

To make progress, we developed a microfluidic platform to study the mechanics of fluid transport by epithelial cells. The device is designed to simultaneously measure fluid flow across the epithelium and careful control/monitoring of hydraulic pressures at the apical and basal sides of the epithelium. Using this device, we show that kidney epithelial cells are active mechanobiological fluid pumps, i.e., they generate a hydraulic pressure gradient across the epithelium (opposite of the fluid flow direction), and the pump stalls when the pressure gradient is too high. Using live cell imaging, we show that due to formation of asynchronous spatio-temporal cortical invaginations, hydraulic pressure gradient decreases the baso-lateral localization of Na^+^/K^+^ ATPase by disrupting the F-actin cortex. We also used this device to examine human primary kidney epithelial cells as well as human patient-derived ADPKD cystic cells and mouse PKD2 knockout cells. The device is able to capture the influence of osmotic and apical shear stress on the epithelial pumping action and explicitly demonstrate the reversed pumping action of ADPKD cells. Moreover, hydraulic pressure gradients can alter the transcriptome of kidney cells, and ADPKD cells and mouse PKD2 KO cells show an altered response to applied hydraulic pressure gradients. These results together with fluid pumping measurements provide mechanistic insights to the interplay of hydraulic pressure gradients and fluid transport by the kidney epithelium.

## Results

### Kidney epithelial cells are active mechanobiological fluid pumps

**Fig. 1:**
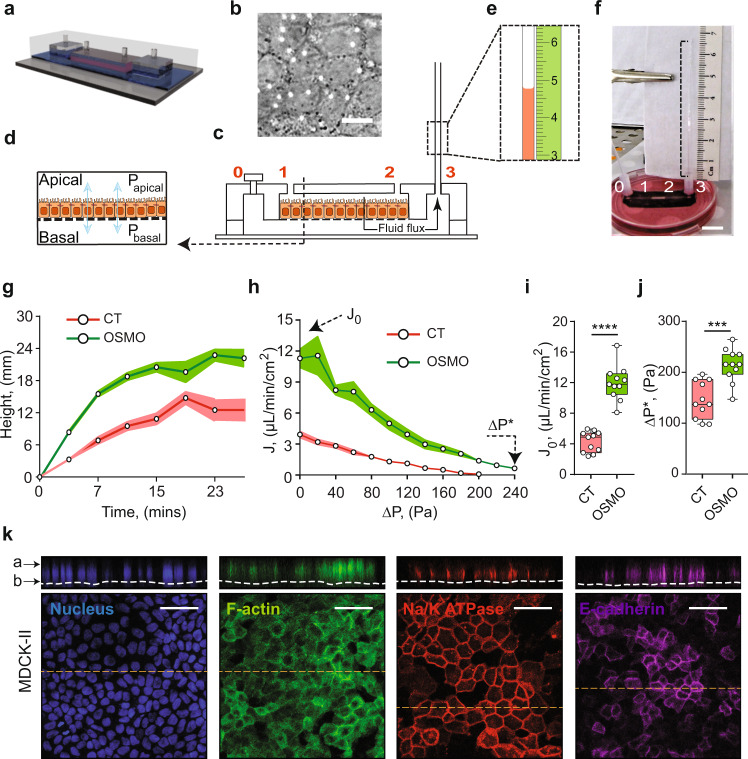
Fluid pumping performance of MDCK-II epithelium. **a** A schematic representation of the Micro-fluidic Kidney Pump (MFKP). **b** A DIC image of the MDCK-II monolayer. White dots indicate pores. Scale bar = 10 mm. **c** Longitudinal section of the device. Black arrows indicate fluid flux. **d** A schematic of a polarized epithelium. **e** Dashed rectangle shows zoomed schematic of fluid flow in the microcapillary (MC) and a mm scale (green). **f** A snapshot of the MFKP inside an incubator for time-lapsed videography. Scale bar = 1 cm. **g** The height of fluid in the MC is plotted as function of time for MDCK-II epithelium in MFKP. Compared to static condition (CT, red) (*n* = 11, *N* = 3, devices, biological replicates), the fluid pumping action changed when the apical medium is switched to 20% hypo-osmotic condition (OSMO, green) (*n* = 11, *N* = 3). Shaded area is the standard error of the mean (SEM). **h** Pump performance curve (PPC) of MDCK-II epithelium, showing the measured trans-epithelial fluid flux (*J*) (apical to basal) as a function of the hydrostatic pressure gradient (Δ*P* = *P*_basal_ − *P*_apical_). *J*_0_ is the fluid flux at zero pressure gradient (Δ*P* = 0) and ΔP* is the stall pressure when *J* = 0. Both *J*_0_ and ΔP* change in the hypo-osmotic condition. Shaded area is the SEM. Comparison of *J*_0_ (**i**) and ΔP* (**j**) for MDCK-II epithelium in the static condition (CT, red) (*n* = 11, *N* = 3, devices, biological replicates,) and 20% apical hypo-osmotic condition (OSMO, green) (*n* = 11, *N* = 3, devices, biological replicates, *p* = 0.0002). ****p < 0.0001 and ***p < 0.001 (two-tailed Mann–Whitney *t*-test). **k** Immunofluorescence (IF) images showing nuclei (blue), F-actin (green), NKA (red) and E-cadherin (purple) in MDCK-II epithelium in the MFKP. Scale bar = 25 mm. For the box plots, lower and upper box boundaries 25th and 75th percentiles, respectively, line inside box median, lower and upper error lines 10th and 90th percentiles, filled circles indicate data points, respectively.

Our micro-fluidic device is designed to mimic the microphysiology of nephron tubular epithelia in the kidney. The device measures the trans-epithelial fluid flux and hydraulic pressure gradient developed across the kidney epithelium while allowing for live cell imaging and simultaneous control of fluid pressure, shear stress (FSS), and media chemical composition (Fig. 1a–f and Supplementary Fig. 6a). The device measures fluidic flux across the epithelium as a function of apical and basal pressures using a microcapillary (MC) connected to port 2 (S2) or 3 (S1) (Fig. 1e, f). The MC simultaneously measures the trans-epithelial fluid flux and the hydraulic pressure (Fig. 1e, f) with a volume resolution of 0.31 μL, and can detect pressure changes of 10 Pa. Calibration experiments were performed to obtain the pressure contribution from the MC due to capillary action and the static pressure profiles of each device for the shear flow condition considered (Supplementary Fig. 6b, c). Flow and pressure profiles of the entire device were also validated by finite element simulation (Supplementary Fig. 6d–i and Supplementary Information).

We first used the well-studied MDCK-II renal cell model to characterize the device. When MDCK-II cells were seeded in the apical channel of the microfluidic device, cells settled on the porous membrane pre-treated with fibronectin and grew to confluence in 2–3 days. Upon further maturation, the epithelium showed classical cuboidal columnar morphology and formed a strong barrier, as tested using a dye permeation assay (see Supplementary Information and Supplementary Fig. 8a–f). Visualization using immunofluorescence (IF) showed that F-actin, Na^+^/K^+^ ATPase and E-cadherin were localized in typical fashion (Fig. 1k). Mature MDCK-II epithelium developed apical to basal fluid flow, which can be visualized as a rise in fluid height in the MC beyond the static equilibrium height (Supplementary Movie 1 and Fig. 1g). The trans-epithelial fluid flux (*J*) from the apical to the basal channel decreased with the hydrostatic pressure gradient (Δ*P* = *P*_basal_ – *P*_apical_) across the epithelium. *J* is maximal when Δ*P* = 0 (denoted as *J*_0_, or zero-pressure flux), and declined until a stall pressure (static head) of ΔP*~100–250 Pa was reached (Fig. 1h). This flux vs pressure curve resembled the classic pump performance curve (PPC) of mechanical fluid pumps. In contrast, for a passive filter, the pressure needs to be higher on the apical side to generate apical-to-basal flow, and the flux is zero when apical and basal pressures are equal (Supplementary Fig. 2, Supplementary Fig. 7a, b). Therefore, kidney cells are active fluid pumps and our device can be considered as a microfluidic kidney pump (MFKP). The developed mechanical force is 30–100 nanoNewtons per cell, and constitutes another force generation mechanism that is perpendicular to the epithelium and opposite to the fluid flux. This may be a general phenomenon for absorptive or secretory epithelia that perform fluid transport. Moreover, we found that PPC changes substantially under mechanical (FSS) and hypo-osmotic gradient (OSMO) perturbations (Fig. 1g–j, Supplementary Fig. 7c, d), indicating active regulation by cells.

### Epithelial domes are pressurized fluid-filled cavities

**Fig. 2:**
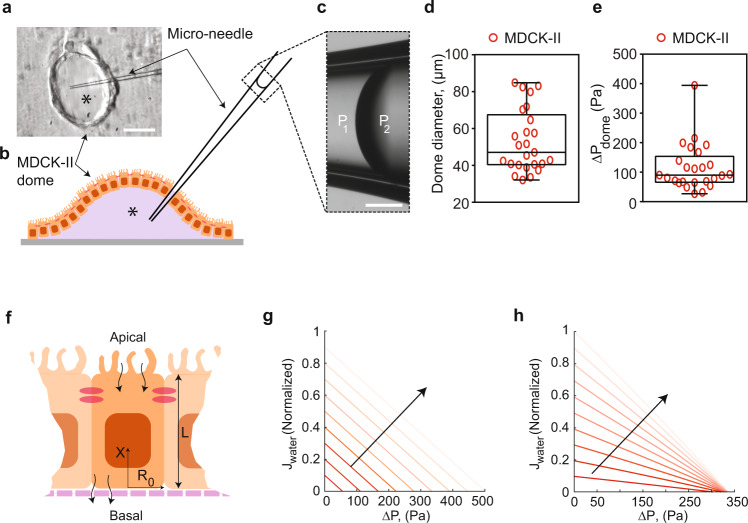
Validation of Δ*P* in epithelial domes and theoretical pump performance curves. **a** Schematic of micro-needle inserted into a fluid filled epithelial dome. Asterisk indicates lumen side in the dome schematic (**b**). **c** Dashed rectangle shows phase-contrast image of oil-media interface in the micro-needle. The curvature of the interface was used to measure the hydrostatic pressure in the domes. P2 and P1 indicate the hydrostatic pressure at the oil-media interface in the micro-needle. Scale bar = 20 µm. **d** Measured dome sizes in an MDCK-II epithelium on glass. **e** Measured hydrostatic pressure in fluid-filled domes in an MDCK-II epithelium on glass. Error bars indicate standard deviation. For the box plots, lower and upper box boundaries 25th and 75th percentiles, respectively, line inside box median, lower and upper error lines 10th and 90th percentiles, respectively, filled circles indicate data points. **f** Schematic of the theoretical model of fluidic pumping by epithelial cells. Flow field along the x-direction and apical and basal surface water and solute fluxes are considered by the model. **g** Theoretical pump performance curve calculated for the model epithelium using active flux in Eq. (19) in Supplementary Information notes, where *J* is the transepithelial fluid flux and ∆*P* is the hydrostatic pressure gradient. **h** Converging nature of PPC to a constant ∆P* was achieved by changing the ${J}_{{active}}^{a}$ to Eq. (20) (Supplementary Information notes). Lighter lines indicate decrease in apical osmolarity. The fluid flux values were normalized against the maximum *J*_0_. Black arrow indicates decreasing osmolarity on the apical side.

To validate the trans-epithelial pressure gradient measured from our device, we examined mature polarized MDCK-II monolayer on 2D impermeable substrates (glass), which formed dynamic fluid-filled domes with elevated internal hydrostatic pressure^18,19^. We measured this pressure by inserting a glass micro-needle into MDCK-II domes while monitoring the curvature of an oil-media interface in the needle (Fig. 2a–c, Supplementary Information). The dome hydraulic pressure is found to be in the same range as measured in the MFKP (Fig. 2d, e), although in domes we were unable to simultaneously monitor the trans-epithelial fluid flux and the hydraulic pressure gradient, which is likely to be near stall pressure. This result, together with traction force measurements of dome pressure^18^, show that MDCK-II epithelium is capable of developing hydrostatic pressure of the order of 200 Pa by actively pumping fluid. In contrast, a confluent layer of 3T3 fibroblast cells and immature MDCK-II epithelia were unable to develop active flux or pressure in our device (Supplementary Fig. 7a, b).

### A theoretical model can explain epithelial pump performance curve

**Fig. 3:**
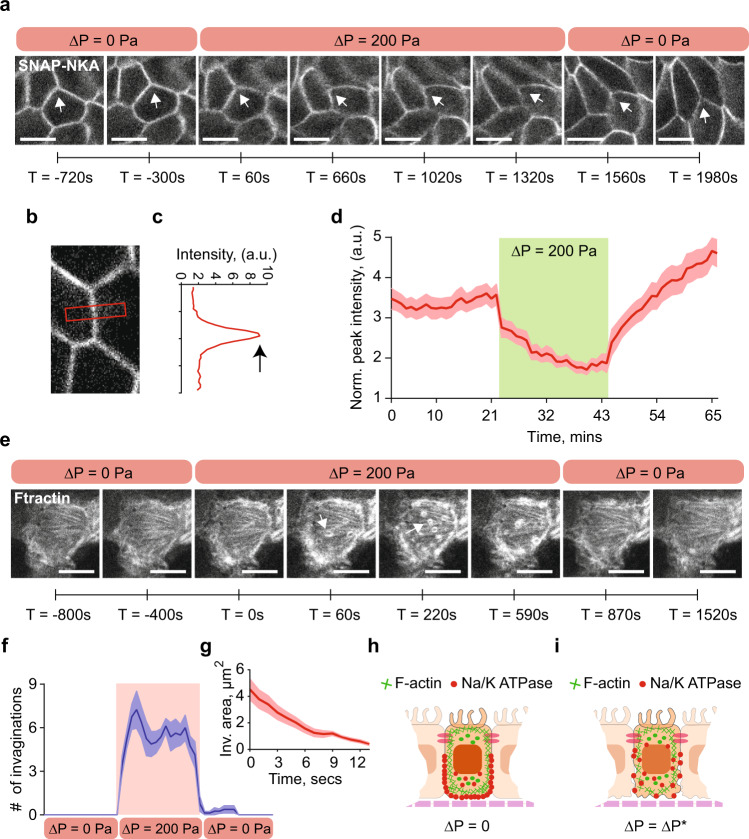
Δ*P* decreases baso-lateral Na^+^/K^+^ ATPase localization by disrupting F-actin cortex. **a** Live cell imaging MDCK-II cells stably expressing SNAP-tagged Na^+^/K^+^ ATPase (NKA) a-subunit and stained with TMR-STAR dye in the MFKP. Time-lapsed confocal images of cells’ lateral side in three consecutive conditions: zero pressure (Δ*P* = 0), pressure gradient (Δ*P* = 200 Pa) and pressure released (Δ*P* = 0). The arrowhead indicates disruption of lateral NKA expression in regions of interest (ROIs) at various time stamps. Scale bar = 10 mm. **b** A zoomed confocal slice of an ROI in MDCK-II cells expressing SNAP-tagged NKA and stained with TMR-STAR dye. **c** NKA intensity is quantified in a red band centered between two cells in **b**. The intensity profile in **c** is the vertical average intensity along the red box in **b**, the peak (indicated by black arrow) is the maximum intensity of NKA. **d** Mean of normalized peak lateral NKA intensity for the same cell over the course of three pressure conditions in **a**. Shading represents the SEM. (*n* = 25, *N* = 3, cells, biological replicates). **e** Live cell imaging of transiently transfected MDCK-II cells expressing GFP-tagged F-actin (Ftractin). Time-lapsed confocal images of the baso-lateral side of the cells were taken under three consecutive conditions: Δ*P* = 0, Δ*P* = 200 Pa, and pressure released (Δ*P* = 0). Scale bar = 10 mm. Pressure application induces rapid high-frequency invaginations in the baso-lateral domain. White arrowhead indicates F-actin rings, which are cross-section of invaginations. **f** Number of invaginations per unit cell over the course of three pressure conditions in **e** (*n* = 8, *N* = 3, cells, biological replicates). **g** Mean area of invaginations as a function of time under pressure. Shading represents the SEM. (*n* = 10, *N* = 3, cells, biological replicates). The invaginations have an average lifetime of ~15 s and disappear when pressure is released as shown in **f**. **h** Schematic showing co-localization of F-actin (green lines) and NKA (red dots) in cells in MFKP at Δ*P* = 0. **i** Hydrostatic pressure gradient results in high-frequency invaginations on the baso-lateral F-actin cortex and a reduction in NKA localization in the lateral domain.

To further understand the PPC curve, we also developed a mathematical model of the PPC based on active transport of an idealized solute (^20,21^, see Supplementary Information). In the model, the cell actively generates an influx of an ideal charge-neutral solute at the apical side, and an efflux of the solute at the basal-lateral side (Fig. 3f). This active passaging of the solute generates a slight osmotic gradient in the cell, which drives the overall flux of water across the epithelium. The model is simple and does not consider possible charges of ions, potential cell-cell coupling, and any active sensing and regulatory mechanisms. The model shows that if the active flux for the ideal solute depends linearly on the osmotic pressure difference across the cell apical and basal surface, then the model predicts a similar PPC that shifts with changes in apical hypo-osmotic gradient (Fig. 2g). On the other hand, if the active flux explicitly depends on the hydraulic pressure at the apical side, then the stall pressure becomes insensitive to osmolarity (Fig. 2h). Depending on the cell type, experimental results appear to fall between these two predicted PPCs.

### Tubular epithelial cells can sense hydraulic pressure to regulate the spatial organization of Na^+^/K^+^ ATPase

In kidney epithelial cells, Na^+^/K^+^ ATPase (NKA) is one of the many ion pumps and transporters responsible for directional Na^+^ transport and generation of osmotic gradients and subsequent water flow, and is a major driving force for vectorial fluid transport^22^. The existence of a stall pressure could be the result of an intrinsic limit in ion pumps such as NKA, or the result of active regulation by cells in response to pressure change. NKA is polarized and accumulates in the cell baso-lateral surface. Blocking NKA by adding ouabain in the device’s apical channel decreased trans-epithelial flux and stall pressure (Supplementary Fig. 9b–d). To visualize NKA dynamics in live cells, we imaged MDCK-II epithelium stably expressing SNAP-tagged NKA α-subunit stained with TMR-STAR dye^23^ while modulating the hydraulic pressure of the basal chamber (Fig. 3a and Supplementary Movie 3). This allowed us to visualize NKA localization in real time using confocal microscopy. Upon application of a pressure gradient (Δ*P*), NKA levels at the cell baso-lateral surface dramatically reduced over several minutes, but recovered when the applied pressure is released (Fig. 3b–d). Comparing IF images of cells in MFKP at Δ*P* = 0 and Δ*P* = 200 Pa showed unchanged F-actin distribution, but NKA showed reduced enrichment at the baso-lateral surface when Δ*P* = 200 Pa is applied (Supplementary Fig. 9e–n). Live cell imaging of F-actin (See Methods) also showed the appearance of highly dynamic F-actin invaginations at the cell basal surface with the application of Δ*P* (Fig. 3e and Supplementary Movie 4). The invaginations have a lifetime of ~15 s and eventually diminished in time when Δ*P* is removed (Fig. 3f, g). It was previously shown that disruption of cortical F-actin leads to disruption of baso-lateral NKA^24,25^. Therefore, we conclude that ∆*P* causes remodeling of NKA localization, which indicates a mechanism of cell pressure sensing and a negative regulatory feedback between pressure gradient and fluid flux.

For MDCK II domes on glass (Supplementary Movies 5 and 6), NKA also showed a similar reduction in enrichment on the baso-lateral domain when the dome is stable (Supplementary Fig. 9o–z). These results indicate that cells can actively sense the apical-basal pressure difference and modulate NKA localization at the baso-lateral surface.

### Human ADPKD cystic cells pump fluid in the opposite direction to normal tubular epithelial cells

Next, we considered whether the device is useful for understanding fluidic pumping by primary normal human kidney cells (NHK) and ADPKD cystic cells from patients with germline *PKD1* mutations. AQP2 (blue), Na^+^/K^+^ ATPase (red) and F-actin (green) stains for cortical cells (NHKc), medullary cells (NHKm) and cystic cells (ADPKD) in MFKP showed the same distribution and morphology as those obtained from immunohistochemistry images of kidney tissue sections (Supplementary Fig. 10a–d, f–k), suggesting that our device is capturing the general organization of these different types of epithelia. The barrier function of the epithelia was again assessed using dye permeation assay and trans-epithelial electrical resistance (TEER) (Supplementary Fig. 11a–i and Supplementary Information).

**Fig. 4:**
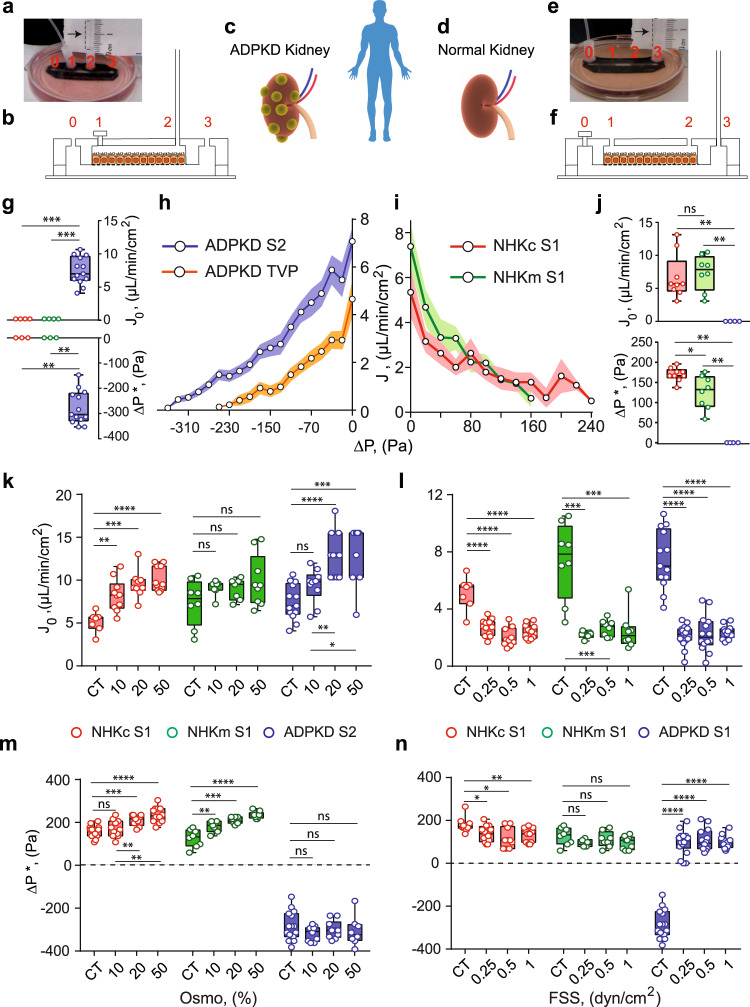
Cystic cells derived from ADPKD patients pump fluid in the opposite direction as normal human kidney cells. **a**, **b** A snapshot and schematic of MFKP in setup-2 (S2). A cystic ADPKD kidney (**c**) and an normal kidney (**d**) derived from patients. **e**, **f** A snapshot and schematic of the MFKP in setup-1 (S1). **g** Measured *J*_0_, and ΔP*, for NHKc (*N* = 4, biological replicates), NHKm (*N* = 4) and ADPKD (*N* = 13) epithelia using S2. For *J*_0_, ***p_NHKc-ADPKD_ = ***p_NHKm-ADPKD_ = 0.0008 and for ∆P*, **p_NHKc-ADPKD_ = **p_NHKm-ADPKD_ = 0.0036. **h** Pump performance curve (PPC) of ADPKD epithelium using S2. (*N* = 13, biological replicates). 10 nM Tolvaptan (TVP) (*N* = 12, biological replicates) decreases fluid pumping in ADPKD cystic epithelium. **i** PPCs of NHKc (*N* = 10) and NHKm (*N* = 8) epithelia using S1. Shaded area is the SEM. **j** Comparison of *J*_0_, and ΔP*, for NHKc (*N* = 9, biological replicates), NHKm (*N* = 8) and ADPKD (*N* = 4) epithelium using S1. For *J*_0_, *p*_NHKc-NHKm_ = 0.5249, **p_NHKc-ADPKD_ = 0.0014, **p_NHKm-ADPKD_ = 0.0040 and for ∆P*, *p_NHKc-NHKm_ = 0.0166, **p_NHKc-ADPKD_ = 0.0028, **p_NHKm-ADPKD_ = 0.0040. Comparisons of *J*_0_ with (**k**) varying hypo-osmotic gradients in NHKc (*N* = 7, 10, 9, 11, biological replicates for CT, 10%, 20 and 50%, **p_CT-10%_ = 0.0018, ***p_CT-20%_ = 0.0002, ****p_CT-50%_ < 0.0001), NHKm (*N* = 8, 7, 7, 9, *p*_CT-10%_ = 0.2319, *p*_CT-20%_ = 0.3969, *p*_CT-50%_ = 0.1388) and ADPKD (*N* = 12, 10, 11, 8, *p*_CT-10%_ = 0.0554, ****p_CT-20%_ < 0.0001, ***p_CT-50%_ = 0.001) epithelia. **l** Fluid shear stresses in NHKc (*N* = 7, 12, 10, 12, biological replicates for CT, 0.25, 0.5 and 1, ****p < 0.0001), NHKm (*N* = 8, 6, 8, 8, ***p_CT-0.25_ = 0.0007, ***p_CT-0.5_ = 0.0006, ***p_CT-1_ = 0.0006) and ADPKD (*N* = 13, 16, 14, 11, ****p < 0.0001) epithelia. **m** Comparison of ΔP* in NHKc (*N* = 12, 11, 9, 12 biological replicates for CT, 10%, 20 and 50% osmotic gradients, *p*_CT-10%_ = 0.4324, ***p_CT-20%_ = 0.0003, ****p_CT-50%_ < 0.0001), NHKm (*N* = 8, 7, 7, 8, **p_CT-10%_ = 0.0099, ***p_CT-20%_ = 0.0002, ****p_CT-50%_ < 0.0001) and ADPKD (*N* = 15, 9, 9, 8, *p*_CT-10%_ = 0.22, *p*_CT-20%_ = 0.594, *p*_CT-50%_ = 0.417). **n** And for fluid shear stresses in NHKc (*N* = 9, 12, 10, 11, biological replicates for CT, 0.25, 0.5 and 1 dyn/cm^2^, *p_CT-0.25_ = 0.0237, *p_CT-0.5_ = 0.015, **p_CT-1_ = 0.003), NHKm (*N* = 8, 5, 8, 9, *p*_CT-0.25_ = 0.1152, *p*_CT-0.5_ = 0.4547, *p*_CT-1_ = 0.1306) and ADPKD (*N* = 15, 12, 14, 12, ****p < 0.0001. Unpaired, two-tailed, Mann–Whitney *t*-test. See Fig. 1 caption for explanation of box-plot.

Once grown in the device, as with MDCK-II epithelium, apical to basal fluid flux was observed as a function of hydrostatic pressure gradient in NHKc and NHKm epithelium, resulting in a similar PPC (Fig. 4i). ΔP* was higher in case of NHKc as compared to NHKm (Fig. 4j). Unlike absorptive function of normal kidney cells, ADPKD cystic cells had a secretory phenotype even though there is no dramatic difference in typical markers of apical-basal polarity^8,26^ (Supplementary Fig. 10a–d). The reversal of fluid flux in cystic cells remains unexplained and the underlying physical parameters of reversal have not been previously quantified. When the MC is connected to port 2 (S2) instead of port 3, we observed fluid rises in the MC beyond the equilibrium height by ADPKD cells, indicating basal-to-apical fluidic pumping (Fig. 4g, h, Supplementary Movie 2), whereas NHKm and NHKc did not develop fluid flux in S2. In contrast, when the MC is connected port 3 (S1), ADPKD cells did not generate any fluid flux (Fig. 4j). In both normal and ADPKD cells, the trans-epithelial fluid flux (*J*) and the PPCs are modulated by mechanical (FSS) and hypo-osmotic (OSMO) perturbations. These changes were quantified by plotting *J*_0_ and ΔP* under different conditions (Fig. 4k–n).

Under apical hypo-osmotic treatment, proximal tubule cells (NHKc) changed their pumping performance by increasing both *J*_0_ and ΔP*. NHKm cells also increased *J*_0_ and ΔP* with apical hypo-osmotic shock (Fig. 4k, m). For ADPKD cells, while *J*_0_ increased with decreasing basal osmolarity, ΔP* didn’t change and remained constant around −300 Pa (Fig. 4m). The converging nature of the PPC to a constant ∆P* for ADPKD epithelium (Supplementary Fig. 12c) cannot be explained by the simple active flux model (see Supplementary Information), suggesting differential mechanisms of regulation during fluidic pumping in normal and ADPKD epithelium.

In both NHKc and NHKm cells, apical FSS for 5 h did not change *J*_0_ or ΔP* significantly (Fig. 4l, n). At 1 dyn/cm^2^, both *J*_0_ and ΔP* decreased for both NHKc and NHKm as compared to control cells. However, in case of ADPKD, even though increasing apical FSS resulted in a decrease in the average *J*_0_, the fluidic flux direction reversed under FSS (Fig. 3l) with ΔP* going from −300 Pa to 100 Pa (Fig. 4n and Supplementary Fig. 12f). IF images indicate an increase in NKA localization on the lateral surface of ADPKD cells under FSS, which could potentially explain the fluidic flux reversal (Supplementary Fig. 18).

### PKD 2 KO in mouse cells decreases fluid pumping efficiency and increases barrier strength

**Fig. 5:**
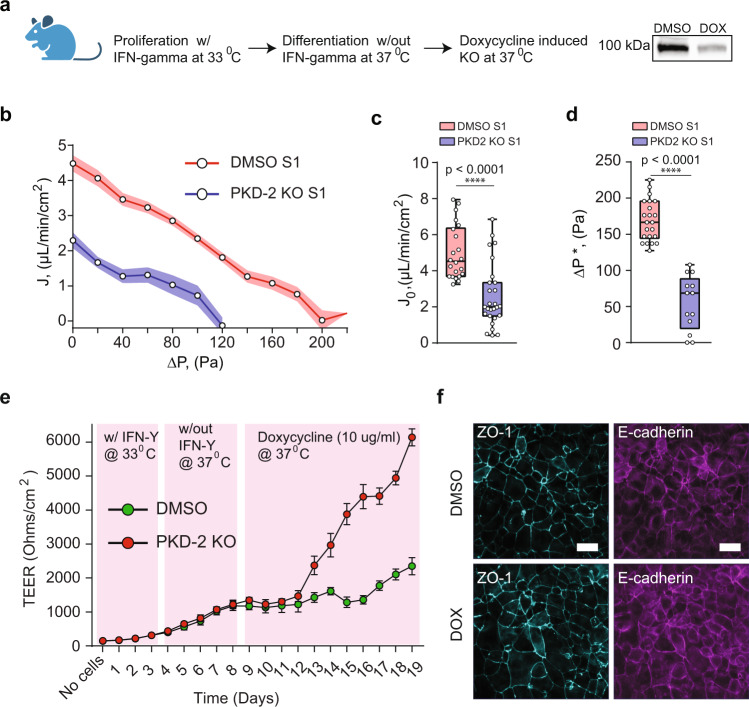
PKD2 KO in mouse kidney cells decreases fluid pumping and increases the barrier strength. **a** Protocol for genetic knock-out in a *Pkd2*^*fl/fl*^, *Pax8rtTA, TetOCre* mouse cell line. Western blot showing depletion of polycystin-2 protein in doxycycline (Dox) treated cells. **b** PPCs for normal (*n* = 22, *N* = 3) vs *Pkd2* KO (*n* = 27, *N* = 3, devices, biological repeats) mouse kidney epithelium. Shaded area is the SEM. Comparison of *J*_0_, (**c**) and ΔP*, (**d**) for normal (*n* = 22, *N* = 3) vs. *Pkd2* KO (*n* = 27, *N* = 3, devices, biological repeats) mouse kidney epithelium. ****p < 0.0001. (Unpaired, two-tailed, standard *t*-test). See Fig. 1 caption for explanation of box-plot. **e** Trans-epithelial electrical resistance (TEER) was measured in mouse cells grown on permeable supports (24 well plate format) (*n* = 15, *N* = 3, independent experiments, biological replicates. Data are presented as mean values ± SEM. PKD-2 was gradually knocked out using doxcycline (10 µg/ml). Compared to DMSO control, PKD-2 KO leads to an increased barrier function in mouse cells. **f** Confocal IF images of ZO-1 (turquiose) and Ecadherin (purple) for DMSO control and Dox (PKD-2 KO).

To confirm our observations from the human primary cells we next utilized an immortalized engineered mouse cell line from the Maryland PKD Cell Culture and Engineering Core with inducible *Pkd2* knockout (*Pkd2*^*fl/fl*^, *Pax8rtTA*, *TetOCre*^20,21^). These cells are clonal, and the inducible nature of the KO provides isogenic controls with low genotypic and phenotypic noise, allowing for mechanistic insights into ADPKD^27,28^. These cells also formed a tight epithelium in our device, and we measured their PPC before and after the application of doxycycline that triggered depletion of PC2 levels (Fig. 5a). Results show that mouse *Pkd2* KO cells lowered apical-to-basal flux, but did not reverse as seen in human ADPKD cells (Fig. 5b–d). Noticeably, baso-lateral localization of F-actin and NKA changed dramatically when PC2 abundance was significantly depleted, which could explain the decrease in apical-to-basal fluid flux (Supplementary Fig. 14). Additionally, PKD2 KO caused a dramatic increase in the barrier strength of the epithelium compared to wild type cells (Fig. 5e). However, no noticeable change was observed in the expression levels of ZO-1 and E-cadherin in both cells types (Fig. 5f). The inducible *Pkd2* KO cell model of PKD provides insights into the most acute changes after the loss of polycystin proteins and the early stages of disease progression^26^; observed phenotypical differences with the human ADPKD mature cysts cells suggests that further transformation may occur after PC2 loss and ADPKD cells from mature cysts behave differently.

### Diseased cells are less sensitive to hydraulic pressure gradients than normal cells in both humans and mouse

**Fig. 6:**
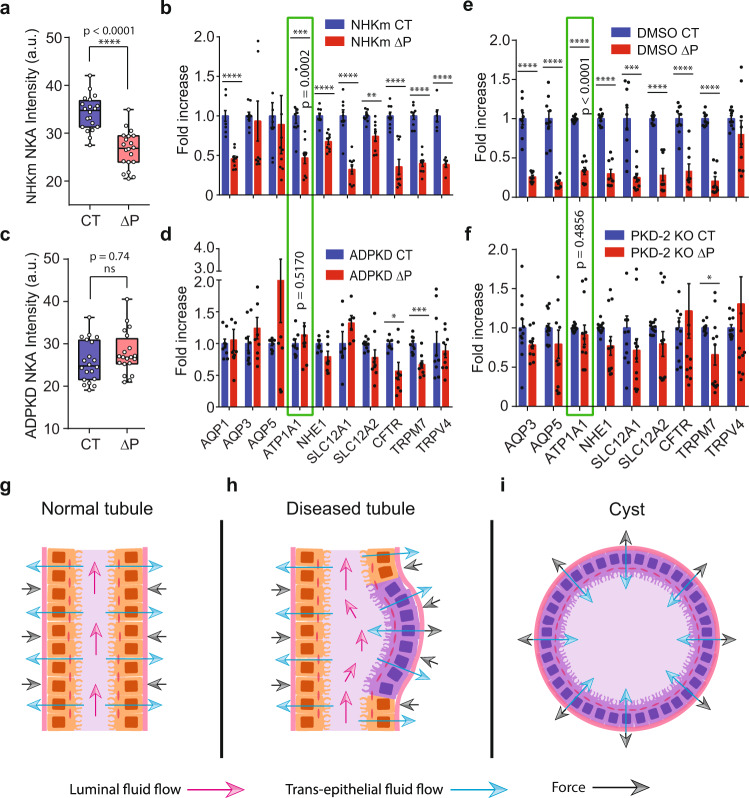
Diseased cells have defects in sensing hydrostatic pressure as compared to normal cells. **a** Total intensity of NKA in NHKc cells at control, CT and pressure, Δ*P*. (*n* = 20, *N* = 3, cells, independent biological repeats). See Fig. 1 caption for explanation of box-plot. **b** Relative expression changes of genes in NHKm cells for CT (Δ*P* = 0) and Δ*P* (Δ*P* = 200 Pa). (*n* = 9, *N* = 3). **c** Total intensity of NKA in human ADPKD cystic cells for CT and Δ*P* = (ns not significant, *p* = 0.74, two-tailed Mann–Whitney *t*-test), (*n* = 20, *N* = 3). **d** Comparison of the same genes ADPKD cystic cells for CT (Δ*P* = 0) and Δ*P* = −200 Pa (*n* = 9, *N* = 3). Green rectangle highlights ATP1A1 (encodes for NKA). For human NHKm cells, mRNA levels of ATP1A1 decreased significantly (***p = 0.0002) under ∆*P* for 10 h but remained unchanged for cystic cells (*p* = 0.5170). For NHKm cells, **p_SLC12A2_ = 0.0012, ****p < 0.0001. For ADPKD cells, *p_CFTR_ = 0.0499, ***p_TRPM7_ = 0.0008. **e** Relative gene expression in inducible mouse cells with DMSO vehicle for CT and Δ*P* (*n* = 6, *N* = 2). **f** Relative expression changes of genes in *Pkd2* KO mouse cells for CT and Δ*P*. (*n* = 12, *N* = 4). For normal mouse cells, ATP1A1 mRNA levels increased significantly (****p < 0.0001) under ∆*P* but not for *Pkd2 KO* cells (*p* = 0.4856). For normal mouse cells, ***p_SLC12A1_ = 0.0003, ****p < 0.0001. For PKD2 KO cells, *p_TRPM7_ = 0.0165. Error bars indicate SEM. (Unpaired, two-tailed, Mann–Whitney *t*-test). **g** A schematic of epithelial cells in MFKP device. Blue arrows indicate absorptive fluid flux. Black arrows indicate the restoring force due to pressure gradient (Δ*P*). **h** PKD2 KO in a mouse cell line decreased the absorptive fluid flux. Decreased pumping force due to diseased cells will cause a force imbalance and may destabilize the epithelium. **i** In human ADPKD cystic cells, fluid pumping reverses in direction causing elevated pressure on the apical side. Hydraulic pressure gradient may aid in expansion of mature cysts.

In ADPKD kidneys, the progressive growth of individual fluid filled cysts leads to a collective increase in total kidney volume (TKV)^29,30^. Currently, the only Food and Drug Administration (FDA) approved pharmacological treatment that decreases TKV is Tolvaptan (TVP)^29,31^, a V2R antagonist and has been shown to decrease cAMP levels^32^. We treated mature ADPKD epithelium in MFKP with 1 nM TVP on the baso-lateral side for 1 h and measured PPC. Interestingly, TVP decreased both *J*_0_ and ΔP* of the ADPKD epithelium as compared to the control (Fig. 4h), confirming the efficacy of MFKP in modeling renal function in-vitro. To further confirm that human kidney cells are sensitive to pressure changes as seen in MDCK II (Fig. 3), and to obtain a molecular profile of kidney cells during fluidic pumping, we performed qPCR measurements for aquaporins (AQP1, AQP3 and AQP5), ion-pumps and exchangers (Na^+^/K^+^ ATPase, NHE1, NKCC1, NKCC2, CFTR), and tension sensitive Ca^2+^ channels (TRPM7 and TRPV4), which are all potentially involved in regulating absorptive and secretory water/ion transport, and mechano-sensation. Heatmaps indicating the expression of mRNAs extracted from NHKc, NHKm and ADPKD cells grown on permeable and impermeable substrate (tissue culture treated polystyrene dishes) show substantial expression differences (Supplementary Fig. 13a). Moreover, we collected cells from MFKP under two conditions: Δ*P* = 0 (CT) and Δ*P* = 200 Pa. Figure 6b, d show that when exposed to pressure Δ*P* = 200 Pa, NHKm cells decreased the expressions of genes, particularly ATP1A1 (NKA). However, ADPKD cells did not respond to Δ*P*, where expressions of these genes either remained constant or increased slightly. Mouse *Pkd2* KO cells also showed substantial expression change similar to human cells when exposed to Δ*P* = 200 Pa as compared to DMSO control (Fig. 6e, f). Interestingly, diseased cells (both mouse and human) did not respond to Δ*P* as normal cells, particularly the mRNA levels of ATP1A1 (NKA) (green rectangle) (Fig. 6b, d, e, f). IF images of F-actin and NKA in the MFKP device corroborates the qPCR results at Δ*P* = 0 and Δ*P* = 200 Pa. The total intensities of NKA in NHKc, NHKm and ADPKD epithelia under the two conditions were also consistent with the mRNA readings from qPCR (Supplementary Figs. 15–17 and Fig. 6a, c). However, spatial arrangement of F-actin and NKA in NHKc, NHKm and ADPKD epithelia showed significant differences at Δ*P* = 0 and Δ*P* = 200 Pa. For all the three human primary cell types, F-actin was generally the highest at the cell basal surface at Δ*P* = 0, but the F-actin stress fiber density was significantly decreased at Δ*P* = 200 Pa (Supplementary Figs. 15–17). Interestingly, Δ*P* disrupted the baso-lateral polarization of NKA in NHKm cells (Supplementary Fig. 16a–r). This depolarization effect was completely absent in NHKc but was subtle in ADPKD cells exposed to Δ*P*. This indicates that collecting duct cells (NHKm population) have altered pressure sensing properties than NHKc and ADPKD cells.

## Discussion

Tubular fluid transport in the kidney has been studied in the past extensively^4^. It is also known that cystic cells in ADPKD kidneys secrete fluid into the lumen due to Cl^−^ ion secretion^3,26,30^. However, these studies were conducted in kidney tissues and perfused tubules without precise control of hydraulic pressure gradients. It is also difficult to exclude the potential effects of myofibroblasts, endothelial cells, and other cells in the ECM, and investigate pressure-sensing properties of kidney epithelial cells. The ability to simultaneously measure the fluid flux versus pressure in a single monolayer epithelium setup makes this study unique; allowing us to map the mechanical fluid pumping performance of epithelial cells themselves. The microfluidic design can be used for any epithelial system to examine fluid transport. The precise pressure control allows for general studies of the effects of hydraulic pressure gradients across the epithelium.

We have shown that kidney epithelial cells can actively generate a hydrostatic pressure gradient (Δ*P*) during fluid pumping, and the cystic epithelium can generate and sustain a pressure gradient of up to ~300 Pa, which acts against the direction of flow. This behavior should be contrasted with passive filtration, which requires a pressure gradient in the direction of fluid flow. It is noteworthy that the observed pressure differences could mechanically deform the epithelium despite large absolute hydraulic pressures in the lumen and the interstitium. Moreover, we discovered that the stall pressure gradient ΔP* depends on environmental conditions surrounding the epithelium and is actively regulated by cells. The regulation of ΔP* is therefore potentially critical for understanding normal kidney function and disease morphogenesis. In response to hydraulic pressure gradient changes, cells actively re-arrange spatial positioning of NKA in a cytoskeleton-dependent manner. The observed pressure sensitivity of the epithelium is rapid, and reveals a connection between hydraulic pressure and epithelial polarity. The pressure gradient sensitivity by epithelial cells also propagates to the proteomic and transcriptomic level, and suggests a mechanism of mechanotransduction that couples hydraulic pressure with both protein and gene expression.

Figure 6g shows a section of a normal human kidney epithelium. Blue arrows represent fluidic flux from the lumen into the interstitial space. Black arrows indicate the restoring force as a result of the fluidic pumping, arising from the apical-basal hydraulic pressure difference during pumping. In situ, the apical-basal pressure difference could prevent tubule dilation in the nephron. As a result of decreased fluid pumping by a few diseased cells, the epithelium will experience spatially varying forces due to the non-uniform pressure gradient, which could lead to dilated tubules as observed in early stages of the disease (Fig. 6h). Additionally, due to altered morphology of the tubule, the local fluid shear stress can decrease, which may lead to reversed direction of fluid pumping by ADPKD cells. Mature cystic epithelium from late-stage ADPKD patients pump fluid in the opposite direction (basal-to-apical) and develop a reversed pressure gradient, which changes the direction of the restoring force towards the interstitial side (Fig. 6i). Fluid secretion^26,30^ together with aberrant pressure sensing response of ADPKD cells, coupled with force-dependent cell proliferation^33^, can lead to gradual expansion of mature cysts in late stages of the disease. Our results demonstrate that secretory and absorptive functions of epithelia can generate significant mechanical forces, and these forces may be important in tubular morphogenesis in general. Our combined results offer insights into the mechanics of kidney epithelial fluidic pumping action, hydraulic pressure gradient transduction and the mechanobiology of ADPKD cyst development.

## Methods

### Fabrication of micro-fluidic kidney pump (MFKP)

The MFKP device has four primary components- micropatterned PDMS block (Ellsworth Adhesives 4019862), porous membrane (Sigma Z681822-3EA), micropatterned intermediate layer (Ted Pella 16081-2) and glass slide (WPI FD5040-100) as shown in Supplementary Fig. 6a. The PDMS block was fabricated by mixing PDMS base and curing agent in a 10:1 ratio and pouring over an aluminum master mold, manufactured using standard micromachining. Then it was cured for 12 h at room temperature and for 6 h at 800 °C. The central pattern has dimensions of 6 mm × 2 mm × 250 µm. Two more patterns in the PDMS block have dimensions of 2 mm × 2 mm × 250 µm. Four holes were punched in the PDMS block to create ports for media inlet and exit. The porous membrane has a thickness of 10 µm and pore size of 1 µm, was cut into the right size before bonding it with the PDMS block. A sharp scalpel was used to make patterns with dimensions of 30 mm × 2 mm × 200 µm in the intermediate layer. The PDMS block, the porous membrane and the intermediate layer were bonded using an RTV sealant (Sky Geek 4102963) by manually aligning them to form an assembly. This assembly was then connected to the glass slide after oxygen plasma treating (Plasma Etch PE-25) all components to make final assembly. The interfaces of and the glass slide were sealed with an RTV sealant.

### Calibration for pressure and flux

A syringe pump (New Era Pump Sys NE-1000) with a 30 ml syringe (Fisher Scientific 302832) filled with cell culture media was connected to ports 1 and 2 of the device to apply a range of shear stress and hydrostatic pressure in the chamber A, whereas the chamber B was filled with the same media. Port 0 was closed and port 3 was connected to microcapillary (Sigma P2049-1PAK) (Fig. 1c, e). A mm scale paper ruler (Wintape PT-025) was glued to a holder next to the microcapillary to image the height fluid in the microcapillary using videography. As shown in Fig. 1f, due to the pressure gradient media will flow from channel A to B, though the porous membrane and start filling the microcapillary. As the fluid rises in the microcapillary, the final height (h) was recorded using a camera (GoPro Hero7) by making time-lapsed videography of the entire setup in the incubator. Pressure in channel B can be measured by multiplying density (ρ) of the media and acceleration due to gravity (g) to the measured height (h) of the fluid column in the microcapillary. This is also the average pressure (P_apical_) in channel A due to steady state pressure equilibration (see SM). The average pressure (P_apical_) in channel A was mapped for all shear stresses and pressures P_2_ in port 2 of the devices (Supplementary Fig. 6b). The variability in a single device was calculated by taking 3 pressure measurements in the same device and device-to-device variability was obtained by taking measurements from 5 different devices.

### Cell lines and source

Primary human normal (cortex and medulla) and ADPKD (cortical cyst wall) renal epithelial cells were received from the Cell Culture and Engineering Core of the Maryland Polycystic Kidney Disease Research and Clinical Core Center (collection and usage of human material under the approval of the University of Maryland, Baltimore Internal Review Board). All primary human cells were cultured in the Maryland CCEC renal epithelia cell media (REC): 1:1 mixture of RenaLife Complete Medium (Lifeline Cell Technology LL-0025) and Advanced MEM medium (Fisher Scientific #12492) with 5% FBS (ThermoFisher #26140-079), 2.2% Pen/Strep (Fisher Scientific #30-002-Cl), 0.6% L-alanyl-Glutamine (Gemini Bio-products #400-106), and 0.03% Gentamicin (Quality Biological #120-098-661).

For live-cell imaging and measurement of Na^+^/K^+^ ATPase (NKA) dynamics, MDCK-II cells were stably transfected with SNAP-HA– Na^+^/K^+^ ATPase α-subunit and the rat β1 subunit (SNAP cells) and were selected in 500 µ/ml G418 and 500 µ/ml Zeocin, which select for transfection with the α subunit and the β subunit, respectively^23^. The cell lines were a gift from Dr. Michael J. Caplan (Yale University).

For live-cell imaging and measurement of actin dynamics, MDCK-II cells were transiently transfected using pEGFP-C1 F-tractin-EGFP which was a gift from Dr. Dyche Mullins (UCSF) (Addgene plasmid # 58473). Lipofectamine 3000 was used for transfection and selected with G418 Sulfate. Standard protocols for lipofectamine based transfection on 24 well plate were followed.

Immortalized single cell preparations from Pkd2^*fl/fl*^, Pax8rtTA, TetOCre, Sv40% were generously provided by the CCEC Core of the Maryland PKD Research and Clinical Core Center. These cells were cultured on collagen coated transwells with 0.4 µm pores (Corning 3491) at 33^0 ^C. The cells were propagated at this temperature until five days past confluency and fed with the Maryland CCEC renal epithelia cell media (REC): 1:1 mixture of RenaLife Complete Medium (Lifeline Cell Technology LL-0025) and Advanced MEM medium (Fisher Scientific #12492) with 5% FBS (ThermoFisher #26140-079), 2.2% Pen/Strep (Fisher Scientific #30-002-Cl), 0.6% L-alanyl-Glutamine (Gemini Bio-products #400-106), and 0.03% Gentamicin (Quality Biological #120-098-661). For propagation, 10 ng/mL interferon-gamma (CST 39127) is also added to the media when cultured at 33 °C. Polarized plates were then moved to 37 °C and replaced with REC media without interferon-gamma. These cells have been validated to model polycystic kidney disease^27^.

### Culturing cells in MFKP

Prior to seeding cells, all the devices were oxygen plasma treated to increase hydrophilicity and maintain sterility. The devices were then rinsed in 70% ethanol for 10 min and then washed thoroughly with cell culture media and kept filled with the same media for 12 h in an incubator maintained at 37 °C and 5% CO_2_. To improve cell adhesion, the channels were filled with 50 µg/ml of fibronectin solution for overnight at 4 °C. The fibronectin solution was then washed off and the devices were thoroughly rinsed with cell culture media and incubated for 12 h, before seeding cells in the chamber A of the device.

MDCK-II and 3T3 fibroblast cells were passaged at least once in growth media comprising of DMEM1x (Corning 10-013-CV), supplemented with 10% Fetal Bovine Serum (FBS) (ATCC 30-2020) and 1% Penicillin-streptomycin (ThermoFisher 15140163), before seeding in the device.

However, normal human kidney (NHK) cells and cystic cells from ADPKD patients were not passaged in order to avoid fibroblast overgrowth but seeded directly into the microfluidic device after thawing the vials containing frozen cells. After thawing with warmed up REC media, the cell solution was gently centrifuged at 112 × *g* for 2 min. The cell pellet was then collected by discarding the supernatant and then resuspended in the warmed up in 200 µL of REC media. The cells were seeded at very high density using a 1 ml syringe (BD 309628) with 18-gauge blunt tips (McMaster 75165A676) and grew to confluency over 2–3 days which was evaluated using a phase-contrast microscope. At day 1 post-seeding, the non-adherent cells were washed out by gently adding media through port 1 in chamber A. The media was exchanged every 2 days for 2 weeks post confluence to form a tight mature epithelium before preparing for experiments. The devices with incomplete monolayer as observed by phase-contrast microscope were discarded.

The mouse inducible cells were grown using growth media supplemented with Interferon-gamma^2^ in MFKPs placed inside incubators at 33 °C. Once confluent, the devices were then transferred to incubators at 37 °C without media containing Interferon-gamma to enhance differentiation into primary cell-like phenotype. Following a 4-day differentiation period, cells in MFKP were treated with either 10 µg/ml of doxycycline (Sigma D3072) to inactivate Pkd2, or DMSO for controls, for 5 days. The media was exchanged every day before preparing for experiments. The devices with incomplete epithelium as observed by phase-contrast microscope were discarded.

### SNAP tag labeling, pressure perturbation and live-cell imaging

SNAP-tagged NKA in MDCK-II cells was tagged with cell permeable dye TMR-STAR (New England Biolabs, Inc.). SNAP-tagged MDCK-II cells were grown in MFKP for a week to let the cells mature into a monolayer. Normal growth media was perfused every day on both apical and basal side using a syringe. On the day of experiment, cells were incubated for 30 min with 3 µM TMR-STAR dye solution in normal growth media. The cells were then washed three times very gently using normal media to get rid of residual TMR-STAR in the device. MFKPs were then incubated for 1 h in the incubator before imaging.

A mcirocapillary (MC) (Sigma P2049-1PAK) in port-0 and a 1 ml syringe (BD 309659) with 400–500 µL media in port-3 were connected. By gently pushing the plunger media was let into the MC and appropriate pressure gradient on the basal side was achieved by changing the height to fluid in the MC. The same setup was used to perform live-cell imaging of cells under fluid pressure and to condition cells for qPCR experiments.

Confocal imaging (Zeiss) was used to take fluorescence images of one field of view (slice) on the baso-lateral domain of SNAP-tagged MDCK-II cells and F-tractin MDCK-II cells at 1 min interval. The intensity analysis was done using FIJI (ImageJ version 1.41).

### Epithelial barrier strength

The trans-epithelial electric resistance was measured using off-the-shelf TEER measurement device (EVOM-2, WPI Inc, Sarasota, FL) using Ag/AgCl chopstick electrodes (STX-2) with cells seeded on track-etched PET membranes with 1 µm pore diameter (Corning 353104). For each measurement, the plates were taken out of the incubator and equilibrated to room temperature for about 10 min in the biosafety cabinet (during this time the chopstick electrodes were equilibrated in room temperature culture media). Three measurements for each well were taken, measuring the blank transwell in between each round. The order of the wells was random each time. The reported TEER values were calculated by subtracting the average blank reading (no cells) for each set.

To test the permeability of the epithelium 5% v/v FITC-conjugated dextran (FITC-Dex, Sigma) solution with a molecular weight of 2000 kDa was added to the apical side (chamber A) of the device and the basal side sealed off. Confocal imaging (Zeiss) was used to take fluorescence images of 20 field of views from the basal to the apical side each 20 µm apart. Stacks of images were taken every hour for 3–4 h at the same location. The final intensity (total-background) of the field of the views were calculated using FIJI software (ImageJ version 1.41) and plotted against the distance. Control experiments were done without cells in the device, to estimate the diffusion characteristics of the dye through the porous membrane.

### Pump performance curve

Before starting the experiment, all connectors, tubes and micropipettes were washed with IPA (Sigma W292907), rinsed thoroughly with RO water, dried and then oxygen plasma treated before use. In case of NHK cells, after about 3 weeks of confluence, micropipettes were connected to port 3 of the devices and port 0 is closed with a stopper thereby sealing the basal side. This setup was placed inside the incubator such that the micropipette is aligns with the mm scale ruler and the fluid flux in the microcapillary has been recorded at a rate of 60 frames/s. The height vs time profile was calculated by analyzing the video prepared by stitching the time lapse pictures. The fluid flux was then calculated by multiplying the cross-sectional area of the microcapillary to the slope of the height vs time curve. In case ADPKD cells, micropipettes were connected to port 2 of the devices and port 1 is closed with a stopper thereby sealing the apical side. For fluid shear stress perturbation, a syringe pump was connected to the port 2 and 3 to apply physiological levels of shear stress. For AVP perturbation, the micropipette side was first sealed and then media containing different concentrations of AVP was added and incubated for 30 min before starting the time-lapse videography. Similarly, for osmotic perturbation, the micropipette side was first sealed and then hypotonic media was added to the device before starting the time-lapse videography.

### Immunohistochemistry

Fixed (4% paraformaldehyde) human kidney tissue samples were received from the Cell Culture and Engineering Core of the Maryland Polycystic Kidney Disease Research and Clinical Core Center (collection and usage of human material under the approval of the University of Maryland, Baltimore Internal Review Board). Tissue samples were first washed in 1X PBS, then three times for 10-min washes on a rocker at 4 °C. Tissue samples were then embedded in paraffin, sectioned, and mounted onto glass coverslips with a gelatin coating solution (50 ml distilled water, 0.25 g Gelatin, 25 mg Chrome Alum). Deparaffination of tissue samples was performed by washing coverslips in a coplin jar with xylene and ethanol (5 min in 100% Xylene, 5 min in 100% Xylene, 5 min in 100% Ethanol, 5 min in 95% Ethanol, 5 min in 70% Ethanol, 5 min in distilled water, 5 min in distilled water). Following the last wash, cover slips were placed in a heat induced epitope retrieval (HIER) solution, pH 8.0 (1 mM Tris (American Bioanalytical AB02000-01000), 0.5 mM EDTA (Sigma E5134) in final volume of 300 mL of distilled water) with 0.02% SDS (American Bioanalytical AB01920-00500). Samples were warmed in HIER solution with SDS to 100 °C, then transferred to a 100 °C water bath for 15 min. Following this incubation, samples were allowed to cool to room temperature, and washed with distilled water and 1XPBS. Samples were treated with 2–3 drops of Image-iT FX Signal Enhancer (Molecular Probes 136933) for 15 min with rotation at room temperature, and then blocked in Incubation Media [1% BSA (Sigma A7638), 0.1% Tween 20 (BioRad 170-6531), 0.02% sodium azide (Sigma S2002) in a final volume of 50 mL 1X PBS (Bio-Rad 161-0780)] with 1% donkey serum (Sigma D9663) for another 15 min with rotation at room temperature. Primary antibody was added in incubation media and serum solution [AQP2 (Wade 95 chicken, 1:600)] and NKA (Clone C464.6, Millipore, 05-369, Lot # 3061212, 1:200)] and incubated overnight in a humidifier chamber at room temperature. Coverslips were then quickly washed three times in 1X PBS, with a fourth wash lasting for 30 min. Secondary antibodies [1:200 Goat anti-chicken secondary antibody (Invitrogen A-21449) and 1:200 Goat anti-mouse secondary antibody (Invitrogen A-11001)] were added in Incubation Media with serum and incubated at room temperature, in the dark for two hours. Cover slips were then again quickly washed (3X) in 1X PBS with a fourth wash lasting 30 min. Following washes, cover slips were mounted onto glass slides with Vectashield Mounting Media [Vector Labs H1000], and sealed with nail polish. Samples were imaged on a Olympus IX83 inverted imaging system.

### Immunofluorescence

Before fixing both chamber A and chamber B were rinsed gently and thoroughly with PBS (ThermoFisher 14190250). The cells were fixed by adding 3% paraformaldehyde (Electron Microscopy Sciences 15714-S) in PBS in the apical and basal side of the device on ice. After 15 min the paraformaldehyde was washed off by gently adding PBS three times with a gap of 5 min between each wash. The cells were then permeabilized for 10 min using 0.1% Triton (Sginam T- 9284) in PBS. The cells were then washed with PBS in the same fashion as previously on ice. 5% FBS (ATCC 30-2020) in PBS was used for 30 min on ice as blocking agent. The cells were then incubated with primary antibodies [AQP2 (Clone P41181, Invitrogen, PA538004, Lot # SI2454403, 1:200) and mouse NaKATPase (Clone C464.6, Millipore, 05-369, Lot # 3061212, 1:200), rat ZO-1 (Clone R40.76, Santa Cruz, SC-33725, 1:200) and rabbit anti-E-Cadherin (24e10, Cell Signaling, 3195 T, Lot # Lot# 04/2014, 1:200] with 2.5% FBS in PBS overnight at 40 °C on a rocker. The primary antibody was washed off using PBS consecutively for three times with 5 min interval between each wash. The cells in the device were then incubated with secondary antibodies: Donkey anti-rabbit secondary antibody (Clone- Poly4064, Biolegend, 406410, Lot # B243995, 1:200), Goat anti-mouse secondary antibody (Invitrogen, A-21235, Lot # 1939631, 1:500), Goat anti-rat secondary antibody (Invitrogen, A-21247, Lot # 1921562, 1:200) and Donkey anti-rabbit secondary antibody (Clone- Poly4064, BioLegend, 406416, Lot # B243796, 1:200) with 2.5% FBS in PBS for 1 h at room temperature in the dark. Traces of the secondary antibody was removed from the device by washing the cells with PBS consecutively for three times with an interval of 5 min between each wash. F-actin was stained using acti-stain 488 Phalloidin (Cytosckeleton PHDG1-A) at 1:100 with 2.5% FBS in PBS for 1 h at room temperature. The devices were then filled with Vectashield mounting media (Vector Labs H-1200), sealed with parafilm and stored at 4 °C.

### Western blot

Immortalized Pkd2^fl/fl^, Pax8rtTA, TetOCre, Sv40% cells from transwells were lysed with Deoxycholic Acid RIPA buffer (1% deoxycholic acid, 1% triton X-100, 0.1% SDS, 150 mM NaCl, 1 mM EDTA, 10 mM Tris HCl pH 7.5) with 1:10 protease inhibitor (Sigma P-8340). Cell lysate was rotated in the cold room at 4 °C for 30 min and then centrifuged for 15 min at 21,913 × *g*. Supernatant was collected and protein abundance was quantified by bicinchoninic acid (BCA) assay (Thermo Scientific 23225). Samples were heated with 5X laemmeli buffer with sodium dodecyl sodium (SDS) and 10% 2-beta-mercaptoethanol for 30 min at 37 °C. Samples were then loaded on 10% stain free gels (BioRad 4568033) with kaleidoscope marker (BioRad 161-0375) and run for 50 min at 200 V. Before transfer, gels were crosslinked using UV (BioRad ChemiDoc MP Imaging System) and imaged to quantify total loaded protein (LC) for normalization of protein abundance. Gels were then transferred onto 0.2% nitrocellulose membranes (BioRad 1704158) using semidry BioRad Trans-blot Turbo System. Membranes were blocked in 5% Milk in 1X TBS-T and primary antibodies (1:1000 rabbit PC2; Maryland PKD Research and Clinical Core Center #3374) were incubated overnight in 2.5% milk in 1X TBS-T at 4 °C. Blots were washed three times in 1X TBS-T, then incubated with secondary antibody (1:5000 goat anti-rabbit HRP; Jackson ImmunoResearch Laboratories 111035144) in 2.5% milk in 1X TBS-T for 1 h, rocking, at room temperature. Blots were washed again three times with 1X TBS-T and developed in Pico (Thermo Scientific 34577). Blots were developed using Biorad Chemidoc Imaging machine and quantified using Image Lab (BioRad Version 6.0.1 build 34). Normalization was done by calculating the total protein loaded in each lane using the BioRad Stain-Free Gel System. Statistical comparisons of density measurements from western blots were done with the Student’s *t* test for pair-wise comparisons (Prism 7, GraphPad, USA). All reported means are ± standard error of the mean (SEM).

Cystic and non-cystic cells plated on impermeable (regular cell culture dish) and permeable (porous membrane) substrates were extracted for western blot experiments. For lysis, cells were washed twice with PBS and incubates with 100 µL lysis buffer (25 mM Sodium Phosphate pH7.2, 150 mM NaCl, 10% Glycerol, 1 mM EDTA, 1% Triton, Protease Inhibitor (Roche 11873580001)) for 3 min. Plates were scraped and cell solution transferred to 1 ml tube. Cell lysates were incubated on ice for 30 min with regular vortexing. Cell debris was pelleted by centrifugation at 15,000 × *g*. samples were prepaired fro SDS-PAGE and equal amounts of protein (75 µg for each sample) were loaded onto 3–8\% Tris-Acetate SDS-polyacrylamide precast gel (invitrogen). Membranes were incubated with primary antibodies overnight, washed with TBS-Tween 20 buffer then incubated with florescent secondary antibodies for detection using a Biorad chemidoc system. Primary antibodies used for immunoblot included mouse anti-PC1 (7e12) from Santa Cruz Biotechnology (sc-130554, 1:500) directed against the LRR; rat monoclonal E8 antibody raised against PC1-CTF (1:1000); Rabbit polyoclonal 3374 antibody raised against C-terminal tail of PC2 (1:1000); mouse anti-β-actin from Sigma-Aldrich (A5316, 1:10,000).

### Cystic fluid analysis

Cyst fluid, collected from individual cysts of nephrectomized ADPKD kidneys, was received from the Cell Culture and Engineering Core of the Maryland Polycystic Kidney Disease Research and Clinical Core Center (collection and usage of human material under the approval of the University of Maryland, Baltimore Internal Review Board). Collected fluid is stored at −80 °C until time for analysis. Electrolyte analysis is performed using the EasyLyte (Medica Corporation, MA), following the manufacturer’s protocol.

### Gene expression analysis

RNA isolation was performed by using the Direct-zolTM RNA MiniPrep Plus (Zymo Research, Irvine, CA) according to manufacturer’s instructions. RNA was reverse transcribed by QuantiTect Reverse transcription Kit (QIAGEN, Germantown, MD) and real time PCR was performed by SYBR Green Supermix (Bio-Rad Laboratories, Hercules, CA) using specific primers presented in Table 1 (for human cells) and Table 2 (for mouse cells). The relative amounts of each mRNA were quantified using the ∆CT method with 18S rRNA as a housekeeping gene. Each experiment was performed two independent times with two or three technical replicates each time. Samples which produced background CT values were discarded. Heatmaps were generated in R by applying the heatmap.2 function and Z-score was calculated using the formula: *Z* = *(χ* *−* *µ)/σ*, where *χ* is the amount of each mRNA, *µ* is the population mean and *σ* is the population standard deviation.

**Table 1:** Forward and reverse sequences of primers for the genes used for gene expression analysis in human cells.

Gene Name	Forward sequence (5’−3’)	Reverse sequence (5’−3’)
AQP3	CTCGTGAGCCCTGGATCAAGC	AAAGCTGGTTGTCGGCGAAGT
AQP5	CAGCTGGCACTCTGCATCTT	TGAACCGATTCATGACCACC
ATP1A1	TTAATCCCCCAGGCCTCACT	TCTGGTTATGCCAGAGTGACTG
NHE1	ACCTGGTTCATCAACAAGTTCCG	TTCACAGCCAACAGGTCTACCA
SLC12A1	AGTGCCCAGTAATACCAATCGC	GCCTAAAGCTGATTCTGAGTCTT
SLC12A2	TGGGTCAAGCTGGAATAGGTC	ACCAAATTCTGGCCCTAGACTT
CFTR	AGGCAGCCTATGTGAGATAC	TCCGGAGGATGATTCCTTTG
TRPM7	GGAGATGCCCTCAAAGAACA	TGCTCAGGGGGTTCAATAAG
TRPV4	CCCGTGAGAACACCAAGTTT	GTGTCCTCATCCGTCACCTC
18S rRNA	CAGCCACCCGAGATTGAGCA	TAGTAGCGACGGGCGGTGTG

**Table 2:** Forward and reverse sequences of primers for the genes used for gene expression analysis in mouse cells.

Gene Name	Forward sequence (5’−3’)	Reverse sequence (5’−3’)
AQP3	TTTGGACCTCGCCTCTTCAC	TGGTACACGAAGACACCAGC
AQP5	TGAACCCAGCCCGATCTTTC	CGAGGAGGGGAAAAGCAAGT
ATP1A1	CCGAGGAGCTGGATGACATC	AGAGCCAACAATCCCCATGG
NHE1	GCAAGAAACAAAGCGCTCCA	AGCTCAATGGCCTGCTTCAT
SLC12A1	AACTCAGTGCCCAGTAGTGC	CGTGGTGTCTGTTTTGGCAG
SLC12A2	GGCTGCGTTGCTCACATATG	TGCACATGGCCACAGATCAT
CFTR	AGCAGTTTCCTGGACAGCTC	ATGGGGTCTAGATGGGCACT
TRPM7	GTGGCCATACTCACTGCACT	AAGGATCCAACCAGCCACTG
TRPV4	CGCCTACTATCAGCCACTGG	GGAGCCATCGACGAAGAGAG
18S rRNA	CAGCCACCCGAGATTGAGCA	TAGTAGCGACGGGCGGTGTG
18S rRNA	CAGCCACCCGAGATTGAGCA	TAGTAGCGACGGGCGGTGTG

### Statistics and reproducibility

Statistical analysis was performed following standard methods using Graphpad Prism7 software. The micrographs shown in Figs. 1b, k, 2a, e, 4a (right panel) and in Supplementary Figs. 7c, 10a, c, f, h, j, 13b, e, 14b–c, 15e–l, 16e–l, 17e–l, 18 are representative of experiments done at least 3 times independently.

### Reporting summary

Further information on research design is available in the Nature Research Reporting Summary linked to this article.

## Supplementary information

Supplementary Information

Reporting Summary

Description of Additional Supplementary Files

Supplementary Movie 1

Supplementary Movie 2

Supplementary Movie 3

Supplementary Movie 4

Supplementary Movie 5

Supplementary Movie 6

## Data Availability

All data supporting the findings described in this manuscript are available in the article and in the Supplementary Information and from the corresponding author upon reasonable request. Source data are provided with this paper.

## Source data

Source Data

## Code availability

Matlab (R2017b) codes for plotting figures are available at Github: https://github.com/sxslabjhu/pump_code.
