# Supplementary material for: Kidney epithelial cells are active mechano-biological fluid pumps
Source: Nat Commun. 2022 Apr 28;13:2317. doi: 10.1038/s41467-022-29988-w (PMC9050750; doi:10.1038/s41467-022-29988-w)
Supplement: Supplementary file 1 — Supplementary Information [file 41467_2022_29988_MOESM1_ESM.pdf]

Kidney Epithelial Cells are Active Mechano-biological Fluid Pumps: Supplementary Information

April 4, 2022

This document includes:
Supplementary Notes (1-7)
Supplementary Figures (1-18)
Supplementary References

Supplementary Note 1: Hydraulic pressure profile in MFKP

Device calibration

The hydraulic pressure profile in the apical channel of MFKP was measured under different physiologically relevant fluid shear stress (FSS) and hydrostatic pressure gradients across the device. Fluid flow rate (Q) in the apical channel was changed using a syringe pump connected to port 1 and FSS was calculated using:

$$\tau = \frac{6\mu Q}{ab^2} \tag{1}$$

where τ is the fluid shear stress, μ is the fluid viscosity, Q is the flow rate, a is the width and b is the height of the channel. Fluid flow in the apical channel is due to the pressure gradient developed by the syringe pump between port 1 and 2 in order to maintain a constant flow rate (**Supplementary Fig. 1**). Here, J_1, J_2 and J_3 are the fluid fluxes through port 1, 2 and 3 and P_1, P_2 and P_3 are hydrostatic pressures at port 1, 2 and 3 respectively. From mass balance, the fluid fluxes through the ports are linked according to Eq. 2:

$$J_1 = J_2 + J_3 \tag{2}$$

$J_1 \equiv Q$ is a known constant that is maintained by the syringe pump. P_1, however is unknown. The average pressure in the apical channel (P_{apical}) is greater than the basal channel (P_{basal}), which drives the apical-to-basal fluid flux, J_3. When port 0 is closed, this fluid rises into the microcapillary (MC) at port 3. Here MC acts as a sensor to measure both the fluid flux J_3 and hydrostatic pressure P_3 at port 3. At steady state, P_{apical} is equal to P_{basal} and $J_3 = 0$. The height of fluid (h) in the MC at steady state was used to calculate the P_3 using Eq. 3, which is also equivalent to the average basal hydrostatic pressure P_{basal}.

$$P_3 = \rho g h \tag{3}$$

while maintaining the same FSS, P_{apical} can be changed by changing the exit pressure at port 2 (P_2). P_2 was varied by changing the height of the reservoir connected to port 2. By measuring the fluid height h in port 3

at $J_3 = 0$, P_{apical} was measured under two different FSS and multiple (P_2) conditions (**Supplementary Fig. 6b**). Horizontal error bars indicate same-device variation (mean $\pm$ standard deviation, n = 3) and vertical error bars indicate device-device variation (mean $\pm$ standard deviation, n = 5). MFKP can therefore be used to apply a variety of physiologically relevant FSS and hydrostatic pressure to an epithelium.

Due to capillary action, fluid also rises in the MC without any excess pressure. The capillary pressure is a constant pressure that we can calibrate and measure. We can take the capillary pressure into account by measuring capillary height. The capillary height was measured for 5 different MCs with cell culture media. Error bars indicate standard deviation (n = 5) (**Supplementary Fig. 6c**). All MCs were oxygen-plasma treated before experiments, which increases the hydrophilicity of glass. This causes the first measured capillary height at around $\approx$ 34mm. Subsequent measurements every five minutes caused a steady state capillary height of $\approx$ 26-28mm. Therefore the 30 mm mark in the MC was used as baseline to normalize the height of fluid in the MC for all experiments with and with out cells.

Fluid flux (J) vs hydrostatic pressure gradient (ΔP) plot without cells

All pump performance plots (PPCs) were measured under static condition unless indicated by the legend titled- FSS. The following conditions were used in the manuscript-

Control/CT - Static control.

Osmo - Apical/basal hypo-osmotic shock.

FSS - Fluid shear stress.

Static condition (Control) means that the epithelium was not subjected to any fluidic shear stresses (FSS) or hypo-osmotic perturbations (OSMO). FSS indicates fluid was perfused on the apical side of the epithelium using a syringe pump. During FSS experiments, to let the monolayer adjust, the syringe pump is started well ahead of starting the camera (which records fluid flow into the capillary).

P_{apical} was also measured under static conditions using the same technique described in the previous section. Once P_{apical} is known, the fluid flux J_3 was plotted as a function of the the pressure difference, $\Delta P = P_{\text{basal}} - P_{\text{apical}}$ in the absence of cells. When P_{apical} is greater than P_{basal}, apical-to-basal (A to B) fluid flux (red) decreases to zero as the system approached equilibrium i.e. ΔP = 0, which is indicated with dashed line in the MC schematic (left) (**Supplementary Fig. 2**).

When P_{basal} is greater than P_{apical} , fluid flux in the MC reverses and Basal-to-apical (B to A) fluid flux (blue) also decreased to zero at ΔP = 0 (**Supplementary Fig. 2**). Slope of the J_3-ΔP plot is related to the permeability of the porous membrane. The slope for the A-to-B is higher than that of B-to-A because, in case of A-to-B the fluid flow is due to both ΔP and capillary effect of the MC.

The capillary pressure of the microcapillaries (MC) used in the device was $\sim$30 mm as shown in (**Supplementary Fig. 6c**). This means that fluid flux is zero at that height, which is also called the equilibrium height (ΔP = 0). This was validated by calibration experiments (**Supplementary Fig. 7a,b** and **Supplementary Fig. 2**). The capillary pressure is a constant background pressure in the setup. Therefore, in order to avoid the influence of the capillary pressure in the PPC measurements, fluid flux and pressure differential were measured by considering changes in the fluid height beyond 30 mm mark in the MC.

Longitudinal experiments

Under static conditions, with no cells or a semi-confluent epithelium, fluid flow across the porous membrane is passive (only due to capillary effect) and it comes to a halt at ΔP = 0 mark. **Supplementary Fig. 2** shows

that without cells, capillary effect of the MC causes the fluid to flow from channel A to B and it stalls until a steady state capillary height (ΔP = 0) is reached. Here, fluidic flux J_3 is zero at ΔP = 0 (red) and the reverse is also true i.e. in case there is too much fluid in the MC, the fluid moves back rapidly from channel B to A and equilibrates to the steady state capillary height. However, in the presence of an epithelium under static condition, the fluid flux, J is non-zero at ΔP = 0, and has a finite value, which is an indication of active flux, which leads to development of the trans-epithelial hydrostatic pressure gradient. The fluid flux comes to a halt at the new pressure gradient called stall pressure (ΔP^*).

Supplementary Fig. 7a shows longitudinal experiments for MDCK-II cells under static conditions. PPCs were plotted every 2 days under no cells, semi-confluent, confluent and mature monolayers. Both zero-pressure flux (J_0) and stall pressure (ΔP^*) were zero for No-cell, C-2D, C, C+2D, where C = confluence and D = days. This shows that a leaky epithelium (with poor barrier function) cannot actively pump fluid and generate differential pressure. However, for C+4D and C+6D, both J_0) and ΔP^* are positive. This indicates that a mature monolayer (strong barrier function) can actively pump fluid and sustain pressure gradients on the order of 200 Pa. **Supplementary Fig. 3a** shows that for MDCK-II monolayers TEER (barrier function) values saturates around 3 days after confluence.

As a negative control, we chose mouse fibroblast (3T3) cells as these cells can keep growing even after confluence, coating the porous membrane entirely. The only difference is that they do not form tight junctions to form a mature monolayer with high barrier function. **Supplementary Fig. 7b** shows longitudinal experiments for 3T3 cells under static conditions. In all the cases- No cells, C, C+2D, C+4D and C+6D, both J_0) and ΔP^* are close to zero. This indicates that progressively coating 3T3 cells can decrease the permeability of the porous membrane (decreasing slope of PPC from No cell to C+6D) but is not enough to generate a pressure difference or active fluid flux.

Finite Element Simulations

To validate the experimental measurement of P_{apical} in our device, the entire microfluidic device has been modeled using the COMSOL Multiphysics software. The system was divided into three domains; apical domain (A), porous membrane and basal domain (B) (**Supplementary Fig. 6d**). Navier-Stokes equation (Eq. 4) was used to model the fluid domain in apical and basal channels and no slip boundary condition was enforced at the walls.

$$\rho\left(\mathbf{v}\cdot\nabla\right)\mathbf{v} = -\nabla p + \mu\nabla^2\mathbf{v} + \rho\mathbf{v}\,, \tag{4}$$

where ρ is density of the fluid, $\mathbf{v}$ is the fluid velocity vector field, p is the pressure, μ is the fluid viscosity and $\mathbf{g}$ is the acceleration due to gravity.

The following set of boundary conditions were used:

Port 0: No Slip boundary condition as the port was blocked.

Port 1: Flux boundary condition where we prescribe flow velocity. Two flow velocities were employed, namely, 0.0047 m/s and 0.0094 m/s corresponding to fluid shear stresses 0.5 and 1 dyn/cm^2.

Ports 2 and 3: The height of port 2 was varied in steps of 15mm from an initial height of 10mm. This was done to recapitulate the exact experimental condition wherein a reservoir was connected to port 2. During calibrations experiments, the exit pressure P_2 was applied by changing the height of the reservoir.

Membrane-Channel interface: Velocity boundary conditions are used at the interface. For velocity parallel to the membrane surface, no slip zero velocity is imposed. For velocity perpendicular to the interface, a vertical velocity between 0-10μm/s can be assigned. This models the fluid flow across the porous membrane.

The computed pressure, velocity and shear stress profiles in the cross section of the device and on the surface of the porous membrane has been plotted using heat maps (**Supplementary Fig. 6d-i**). For both 0.5 and 1 dyn/cm^2, the apical channel had a longitudinal pressure gradient (along the channel direction) of about 5 Pa. Moreover, flows across the membrane did not change the apical pressure significantly ($\sim$ 3Pa). For simulations, P_{apical} was calculated by taking average of the hydrostatic pressures in port 1 and 2 under all FSS and P_2 conditions identical to the calibration experiment (**Supplementary Fig. 6b**). Both experimental and simulation calibration data match closely at low P_2. However, at large P_2, the P_{apical} shows a diverging trend with increase in FSS from 0.5 to 1 dyn/cm^2. This is because increasing the exit pressure P_2 causes an additional increase in the average pressure in the apical channel. Heatmaps show that the pressure (P), fluid velocity (v) and FSS (τ_{xy}) are uniform along the XZ-cross section (**Supplementary Fig. 6e**). The hydrostatic pressure on the apical channel is higher than that of the basal channel. The fluid velocity is maximum at the center of the apical channel and both velocity and FSS are uniform along the width on top the porous membrane (**Supplementary Fig. 6f**).

In order to validate whether P_3 is equal to P_{basal}, we plotted the pressure profile along the apical and basal channel in the YZ-axis. For both FSS 0.5 and 1 dyn/cm^2, hydrostatic pressure below the membrane showed minimal spatial variation, and was similar to that of port 3 (**Supplementary Fig. 6g-i**), indicating that Eq. (3) is accurate for measuring P_3. In summary, device has been calibrated experimentally and by using FEM model for hydraulic pressure profiles in both the apical and the basal channels. Once calibrated, the device accurately reports P_{apical} and P_{basal} within $\pm$10 Pa.

Supplementary Note 2: Barrier Strength of the epithelium in MFKP

MDCK-II cells

Trans-epithelial electrical resistance (TEER) is considered as the the gold standard for estimating epithelial barrier strength [3]. However, in case of MFKP, off-the-shelf electrodes do not fit in the apical and basal channel in the device. Therefore, TEER values were recorded using standard Ag/AgCl electrodes by seeding cells on membrane filters pre-treated with fibronectin. MDCK-II cells showed a sharp rise in normalized TEER value of $\approx$200 Ω/cm^2 in 3 days post confluence. This value then plateaued to $\approx$160 Ω/cm^2 with time (**Supplementary Fig. 3a**). This indicates that MDCK-II cells form a strong barrier post confluence. In the MFKP the barrier strength was quantified by using a FITC-conjugated dextran dye of MW 2000 kDa. The dye was dissolved in cell culture media at 5% v/v ratio and added to the apical channel through port 1 and 2 in the device and allowed to diffuse into the basal channel. Stacks of confocal fluorescence images, $20\mu m$ apart, were taken spanning the basal and apical channels. The diffusion of the dye across the epithelium to the basal channel was measured by quantifying the temporal variation of the average relative intensity of the images. Upon addition of the dye into the device without cells, the intensity quickly increased within a minute of adding the dye to the apical channel, keeping the basal channel closed. Within 5 minutes the dye diffused completely into the basal channel. The dashed line was used to indicate the position of the porous membrane that separates the basal and apical side (**Supplementary Fig. 8a**). This indicates that dye diffusion across the porous membrane is relatively quick (<10min). For MDCK-II epithelium 2 days post confluence, it took about 3 hours for the dye to diffuse into the basal side from the apical side, indicating relatively poor barrier strength (**Supplementary Fig. 8b**). In this case, comparison of the images of the basal and apical channel in the MFKP at $T = 0$hr and at $T = 3$hr indicated slow diffusion of the dye across the epithelium. However, For MDCK-II epithelium that is 2 weeks post confluence, the dye did not diffuse

from the apical to basal channel at all. The average intensity of the images in the basal side remained the same after 3 hours post injection of dye into the apical channel (**Supplementary Fig. 8c**). In this case, comparison of the fluorescence images of the basal and apical channel in MFKP at T = 0 hour and at T = 3 hours indicated no diffusion of dye across the epithelium.

Human Primary cells

For human primary cells from both non-cystic and cystic kidneys, tissue samples were dissected from the cortex, medulla, or cortical cyst wall (see methods). The cells were labelled as Normal human kidney cortex (NHKc), Normal human kidney medulla (NHKm) and cystic (ADPKD) for all experiments. The cells were not passaged thereafter to avoid fibroblast overgrowth. The TEER values for NHKc cells seeded on membrane filters reached a peak of ≈250 Ω/cm^2 after 5 days post confluence and for ≈160 Ω/cm^2 for NHKm cells before plateauing (**Supplementary Fig. 3b**). However, for ADPKD cells the TEER values increased rapidly post confluence and plateaued at ≈400 Ω/cm^2 (**Supplementary Fig. 3b**). This indicates that both normal human kidney and PKD cystic cells exhibit strong barrier function on porous substrates. The steady state TEER value for some batch of cells reached as high as 854.22 Ω/cm^2 for NHKc cells (plot not shown) and 526.5 Ω/cm^2 for NHKm cells. However, sample-to-sample variability was higher for NHKm and ADPKD cells. To measure the barrier strength of primary cells in MFKP, cells were seeded at high density by thawing directly from frozen vials (see methods). Cells grew to confluence in 2-3 days and then MFKPs were perfused with media every day for the next 2 weeks to form a strong barrier between the apical and basal channel. In all three cell types, the FITC dye did not diffuse from the apical to basal channel. The average fluorescence intensity of the basal channel remains the same as without dye after 3 hours post injection in the apical channel (**Supplementary Fig. 11a-c**). The dashed white line is used to indicate the position of the porous membrane that separates the basal and apical channel. The variability of fluorescence intensity in the same device and from device to device was low (**Supplementary Fig. 11g-i**). Horizontal error bars indicate same-device variability and vertical error bars indicate device-to-device variability (mean ± standard deviation, n = 3). In all three types of epithelia, fluorescence images of the basal and apical channels in MFKP at $T = 0$ and at $T = 4$hr indicated no diffusion of the dye through these epithelia as shown in representative images (**Supplementary Fig. 11d-f**).

Mouse inducible cells

Trans-epithelial electrical resistance (TEER) was measured in mouse cells grown on permeable supports (24 well plate format). It takes around 4-5 days of doxycycline (10 μg/ml) treatment to significantly decrease the polycystin-2 (PKD-2) abundance (**Fig. 5a**). Interestingly, the TEER value is significantly higher for PKD-2 KO (≈6000 Ω/cm^2) compared to normal controls (DMSO) (≈2000 Ω/cm^2) (**Fig. 5e**). Confocal IF images of ZO-1 (turquoise) and E-cadherin (purple) for DMSO control and Dox (PKD-2 KO) is shown in (**Fig. 5f**).The ZO-1 and E-cadherin levels do not look different in DMSO control and PKD-2 KO cells.

Supplementary Note 3: Simple theoretical model can explain increase in J_0 and ΔP^* with apical hypo-osmolarity

Our experiments showed that the epithelial layer can actively pump fluid across the epithelium from the apical to basal sides. To mathematically describe this fluid pumping, we consider a steady-state description

and model the flow of water across the epithelium, driven by the passage of an idealized charge-neutral solute. We assume that cells are stationary without translation or deformation. Each cell in the monolayer can be approximated as a cylinder with radius R_0 and height L (**Fig. 2f**). The cell has an apical end (a) and a basal end (b). The direction points from the basal end to the apical end of a cell is defined as the x-direction. $x = 0$ is the basal position while $x = L$ is the apical position. We assume that the fluid flow and solute diffusion and convection only occur in the x-direction within each cell.

In cylindrical coordinates, the Stokes' equation for cytoplasmic water flow in the cell is

$$0 = -\frac{dp}{dx} + \mu \frac{1}{r} \frac{d}{dr} \left(r \frac{dv}{dr} \right) , \tag{5}$$

where p is the intracellular hydrostatic pressure, μ is the dynamic viscosity of the fluid, and v is the velocity of the flow in the positive x direction. The spatial profile of v is

$$v(x, r) = 2\bar{v}(x) \left[1 - \left(\frac{r}{R_0} \right)^2 \right] , \tag{6}$$

where

$$\bar{v}(x) = \frac{1}{\pi R_0^2} \int_0^{R_0} v(r, x) 2\pi r dr \tag{7}$$

is the averaged velocity in each cross section. Eq. 6 results from the non-slip boundary condition at the cell cortex. Substituting Eq. 6 into Eq. 5 gives

$$0 = -\frac{dp}{dx} - \frac{8\mu}{R_0^2} \bar{v} \, . \tag{8}$$

The conservation of mass requires

$$\frac{dv}{dx} = 0 \, , \tag{9}$$

which suggests that $\bar{v}$ must be a constant in x. In this case, p must be linear in x as seen from Eq. 8. We only need the pressure at the apical end, $p^{\mathrm{a}} = p|_{x=L}$, and the pressure at the basal end, $p^{\mathrm{b}} = p|_{x=0}$, to know the profile of the intracellular pressure p. The average velocity of the intracellular flow can also be solved from p^{a} and p^{b} by using Eq. 8, i.e.,

$$\bar{v} = -\frac{R_0^2}{8\mu L} (p^{\mathrm{a}} - p^{\mathrm{b}}) \, , \tag{10}$$

so that $\bar{v}$ is not an unknown from modeling point of view.

Cross-membrane water fluxes occurs at the apical and the basal surfaces due to an osmotic gradient of the idealized solute (mainly Na^+). Our convention is that water fluxes are positive from outside to inside the cell. The continuity relation requires

$$\bar{v} = -J^{\mathrm{a}}_{\mathrm{water}} = J^{\mathrm{b}}_{\mathrm{water}} \, , \tag{11}$$

which gives two equations to solve for p^{a} and p^{b}.

To find the water flux, a model for the solute concentration, c, is needed. The steady-state equation for solute diffusion is

$$\frac{d}{dx}\left(-D\frac{dc}{dx}+\bar{v}c\right)=0\,. \tag{12}$$

So that the intracellular solute flux

$$J_c=-D\frac{dc}{dx}+\bar{v}c \tag{13}$$

must be a constant throughout the cell. This constant is determined by solute boundary fluxes at the apical and basal surface. We also assume that solute fluxes are positive inwards. At the cell apical and basal boundary, the solute flux is composed of a passive part, $J_{c,\text{passive}}$, and an active part, $J_{c,\text{active}}$ [5],

$$J_c|_{x=L}=-J_c^{\text{a}}=-(J_{c,\text{passive}}^{\text{a}}+J_{c,\text{active}}^{\text{a}})\,, \tag{14}$$

$$J_c|_{x=0}=J_c^{\text{b}}=J_{c,\text{passive}}^{\text{b}}+J_{c,\text{active}}^{\text{b}}\,, \tag{15}$$

where the passive flux follows the gradient of solute concentration across the cell membrane, i.e.,

$$J_{c,\text{passive}}^{i}=-g^i(c^i-c_0^i)\,,\quad i=\{\text{a},\text{b}\}\,, \tag{16}$$

where g is the coefficient of passive ion flux, $c^{\text{a}}=c|_{x=L}$, $c^{\text{b}}=c|_{x=0}$, and c_0 is the solute concentration outside of the cell. The expression for the active solute fluxes vary depending the types of solute, the cell type and potential active regulation by the cell, and is discussed below. Equations 14 and 15 serve as two boundary conditions for Eq. 12.

The water flux across the cell surface is determined by both the hydrostatic pressure gradient and solute osmotic gradient:

$$J_{\text{water}}^{i}=-\alpha^i(\Delta p^i-RT\Delta c^i)\,,\quad i=\{\text{a},\text{b}\}\,, \tag{17}$$

where α is the coefficient of water permeation, which can be different at the apical and the basal ends of the cell, R is the ideal gas constant, T is the absolute temperature, and

$$\Delta p^i=p^i-p_0^i\,,\quad \Delta c^i=c^i-c_0^i\,,\quad i=\{\text{a},\text{b}\}\,, \tag{18}$$

where p_0 is hydrostatic pressure outside of the cell.

Now we can examine different models of active solute flux across the cell surface. If we let

$$\begin{aligned}J_{c,\text{active}}^{\text{a}}&=\gamma_c^{\text{a}}RT(c_{in}^{\text{a}}-c_0^{\text{a}}-\Delta\mu_c^{\text{a}}),\\ J_{c,\text{active}}^{\text{b}}&=\gamma_c^{\text{b}}RT(c_{in}^{\text{b}}-c_0^{\text{b}}-\Delta\mu_c^{\text{b}})\,,\end{aligned} \tag{19}$$

we get a PPC as shown in **Fig. 2g**. (parallel lines). This models a simple solute pump where the flux is linearly proportional to the concentrate difference with a stall solute flux determined by $\Delta\mu_c^{\text{a,b}}$ [6]. This model gives an increasing fluidic pumping stall pressure as a function of decreasing osmolarity of the apical fluid, as seen for NHKc epithelium in Fig. 3n.

Alternatively, if the solute pumping flux has a pressure dependence, e.g.,

$$\begin{aligned}J_{\text{active}}^{\text{a}}&=\gamma^{\text{a}}\frac{c^{\text{b}}-c^{\text{a}}}{c_{0,r}}k_p(p_0^{\text{a}}-p_{0,r}),\\ J_{\text{active}}^{\text{b}}&=\gamma^{\text{b}}\frac{c^{\text{b}}-c^{\text{a}}}{c_{0,r}}k_p(p_0^{\text{b}}-p_{0,r})\,,\end{aligned} \tag{20}$$

we get a PPC as shown in **Fig. 2h**. (converging lines). In this case, the stall pressure, ΔP^* of the epithelium is insensitive to osmolarity of the apical fluid, as seen for ADPKD epithelium in **Fig. 4n**.

The model described here is phenomenological in nature, and does not include electrical charges of different species of ions. There is likely a strong coupling between different types of solutes, and a full molecular model is considerably more complex. Nevertheless, the model demonstrates that the basic physics of fluid flow across the epithelium coupled with active solute flow should produce active pumping behavior, as seen in experiments. Moreover, the observed stall pressure is probably due to a combination of active-flux dependence on osmolarity and pressure regulation of active flux. This is supported by observed changes in the localization of Na^+/K^+ ATPase (NKA) as a function of pressure. Therefore, a full molecular model will require understanding of how hydraulic pressure regulates active solute flux.

Supplementary Note 4: MDCK-II Domes as three dimensional epithelial pressure vessels

Validation of ΔP measured in MFKP using MDCK-II domes

To validate the apical-basal pressure difference measured for MDCK-II epithelium in MFKP, we examined fluid-filled domes seen in mature polarized MDCK-II monolayer on 2D impermeable substrates (glass). Domes (or blisters) are likely developed due to trans-epithelial pumping of ions and water following a similar mechanism. The three dimensional hemi-spherical shape is sustained by the hydrostatic pressure gradient developed (ΔP_{dome}) [1]. We measured the ΔP_{dome} by inserting a glass micro-needle-based pressure sensor into MDCK-II domes (**Fig. 2a,b**). The micro-needle has an oil-water interface with a known surface tension and the other end of which is connected to a pressure sensor (**Fig. 2c**). By measuring the interfacial curvature, the hydrostatic pressure in the dome can be calculated using Young-Laplace equation (Eq. 21). Further details of the exact working principle of this device is described elsewhere [2].

$$P_1 - P_2 = \frac{2\gamma}{R}, \tag{21}$$

where $P_1 - P_2$ is the pressure difference across the oil-media interface, γ is the oil-media surface tension for the interface, and R is the mean radius of curvature of the oil-media interface (**Fig. 2c**). Since P_1 is the pressure inside the dome, knowing the hydrostatic pressure in the media then gives ΔP_{dome}. ΔP_{dome} in MDCK-II domes of varying size was found to be in similar range as that measured in case of the MFKP (**Fig. 2d,e**). Note this method does not simultaneously measure fluid flux across the epithelium, therefore we do not know which part of the PPC curve the measurement is probing. However, the method provides estimates on upper bounds in dome pressure. These results, together with traction force measurements of pressure inside the same type of domes [4], show that MDCK-II epithelium can develop hydrostatic pressure of the order of 200 Pa.

Role of hydrostatic pressure gradient on the baso-lateral localization of NKA

Mature MDCK-II domes in epithelia on 2D impermeable substrates (glass) were also used to investigate the effect of hydrostatic pressure gradient on the localization of F-actin and NKA in the cells forming domes. We compared cells that have just formed a lumen, referred to here as a pre-dome or unstable ($\Delta P_{\mathrm{dome}} \approx 0$), with the cells experiencing high pressure (near stall pressure) in mature domes ($\Delta P_{\mathrm{dome}} \approx$

ΔP^*) (**Supplementary Fig. 9o, p**). Time lapse videos show that MDCK-II domes were unstable when the monolayer was immature, and were prone to collapse, likely due excessive fluid leakage (Supplementary Movie 5). However, as the monolayer matures, the domes became stable and eventually reaching a steady size due to a stall pressure (Supplementary Movie 6). We hypothesized that hydrostatic pressure gradient can change the baso-lateral polarization of NKA, which then leads to the decrease in trans-epithelial fluid flux. In both cases the epithelium was a free-standing monolayer, hence the influence of substrate focal adhesions on the localization of NKA can be ignored. Confocal reconstruction of MDCK-II pre-dome showed enrichment of F-actin in the cortex and NKA primarily on the baso-lateral domain (**Supplementary Fig. 9q-t**). However, in case of cells in mature domes experiencing high pressure near stall, NKA expression was depleted in the basal domain (**Supplementary Fig. 9u-x**). F-actin was still cortical in nature. By plotting the fluorescence intensity of each slice in the confocal stack versus the distance, we found that the overall intensity of both F-actin and NKA in the cells also decreased when the cells are experiencing near stall pressure (**Supplementary Fig. 9y, z**). Lattore et al. previously reported mechanical strain heterogeneity in MDCK cells forming domes of controlled sizes and cortical dilution of F-actin in super-stretched cells [4]. Therefore, to decouple the role of stretch from hydrostatic pressure, we chose to investigate relatively less stretched cells only. We found out that both stretched and unstretched cells in mature domes exhibit depolarization of NKA (**Supplementary Fig. 4**). Therefore these results are further evidence that the hydrostatic pressure gradient plays an important role in NKA polarization in MDCK-II cells.

Supplementary Note 5: Phenotypic similarity of cells in MFKP with tissue section under normal and diseased condition

Comparison based on immunostaining

Tissue sections from normal human kidney and cystic human ADPKD kidneys were compared with epithelial monolayers (NHK and ADPKD) grown in MFKP. Immunohistochemical analysis of tissue sections reveal that NKA (red) is expressed on the baso-lateral side of cells in both normal renal tubules and ADPKD cysts (**Supplementary Fig. 10a, b**). AQP2 (blue) is however enriched on the apical or sometimes sub-apical domains for both normal human kidney and ADPKD cysts (**Supplementary Fig. 10c, d**). The asterisk indicates the lumen side in cystic tissue section. This indicates that the usual markers of apico-basal polarity are similar in both normal tubular and cystic cells. The normal cells demonstrate regular cuboidal columnar morphology. However, cystic cells have an irregular, stretchy shape and decreased cell height (**Supplementary Fig. 10a, c**).

Immunocytochemical analysis of normal human kidney cortex (NHKc) grown in MFKP demonstrates co-localization of AQP2 (blue), NaKATPase (red) and F-actin (green). XY image represents top view of the cells on the porous membrane and XZ images show cross-sectional view of the cells along a line of interest (**Supplementary Fig. 10f**). The dashed white line in XZ image represents the porous membrane. Quantification of apico-basal intensity for AQP2, NKA and F-actin in NHKc epithelium showed enrichment of NKA primarily in the baso-lateral domain of the cells indicated by white arrow on the corresponding XZ image. AQP2 intensity was low indicating absence of the protein in NHKc cells, as the cortical tissue primarily is comprised of proximal tubule cells which lack AQP2 (**Supplementary Fig. 10f**). F-actin was fibrous on the basal side with strong enrichment in the cell-cell junctions (indicated by arrow in XZ) was observed. F-actin XZ images demonstrate that NHKc epithelium exhibit the regular columnar cuboidal

epithelium like that of cells in the non-cystic tissue sections (**Supplementary Fig. 10a, f**). In case of NHKm cells, AQP2 was highly enriched in the apical domain and was also present all over the cytoplasm. NKA intensity was low and is primarily located in the basal side of the cells. F-actin was fibrous throughout the cytoplasm and strong enrichment in the cell-cell junctions was observed (marked by arrow in the F-actin XZ image (**Supplementary Fig. 10h, i**). ADPKD cells were found to have AQP2 in on the apical side in a sporadic fashion in the epithelium. NKA was enriched in the baso-lateral domain and is not uniformly distributed all over the epithelium indicating cell type heterogeneity. F-actin was highly fibrous in the baso-lateral domain and unlike normal cells localization in the cell-cell junction was low (**Supplementary Fig. 10j, k**).

Cystic fluid analysis

The progressive growth of fluid filled cysts leads to increase in total kidney volume, which impairs normal function of the kidney in ADPKD patients [7]. Fluid accumulates into the cyst lumen over long period of time and the hydrostatic pressure is sustained by the epithelium lining the cyst. We analyzed the fluid collected from the same cysts from which the cells for PPC experiments were extracted (**Supplementary Fig. 10e**). The average osmolality of the fluid collected from all the 16 cysts was 315.83 mmol/kg, and the concentrations of Na^+, K^+ and Cl^- were found to be 137.19, 4.93 and 100.75 mmol/L, which is in the similar range of normal interstitial fluid [8]. Therefore, even if the cells pump ions and hence water from basal to apical side, the steady state osmolality and ion concentration were similar to interstitial fluid due to water flux into the lumen.

Supplementary Note 6: Normal and diseased cells on permeable and impermeable substrate

Human primary cells

qPCR experiments were performed on mRNA extracted from cells grown on permeable and impermeable supports (tissue culture treated polystyrene) ten important genes involved in regulating ion/water transport and mechano-sensation. Heatmaps indicating the expression of mRNAs extracted from NHKc, NHKm and ADPKD cells grown on permeable substrate (MFKP) and on impermeable substrate (tissue culture treated polystyrene dishes). The rows are normalized such that the relative concentration of across the cells lines has been shown using color code (**Supplementary Fig. 13a**). The genes under investigation included Aquaporins (AQP1, AQP3 and AQP5) and ion-pumps (NKA, NHE1, NKCC1, NKCC2) and ion-channels (CFTR, TRPM7 and TRPV4). Except for TRPM7 and TRPV4, the expression for all other genes was higher in cells grown in MFKP as compared to that on impermeable substrate, suggesting that the monolayer becomes much more physiologically relevant when grown in MFKP. Human ADPKD cystic cells grown on permeable membranes exhibit increased expression of key genes implicated in the secretory pathway- AQP5, CFTR, SLC12A1, SLC12A2 compared to NHKc and NHKm cells (**Supplementary Fig. 13a**). This indicates that human ADPKD cystic cells do not lose their phenotypic characteristics when grown in MFKP and are indeed secretory in nature. Three biological repeats were done for each condition.

Polycystin levels are generally low in adult kidney cells yet it is possible to detect a weak band in each of the samples by western blot. Both Polycystin 1 and Polycystin 2 appear to be expressed at a higher level in the ADPKD derived cells. This indicates that the mutation in the ADPKD kidney is not a full deletion

of either PKD1 nor PKD2 but rather appears to affect expression level. This phenomenon has been noted in animal models where dosage of Polycystins affects cyst growth [9] and overexpression of Polycystin 1 is linked to cystic disease [10]. **Supplementary Fig. 13e-i** confirms that we are able to detect Polycystin expression levels in all samples. The absence of PC1U in the NHK cells likely reflects the weakness of the bands as the PC1U band is typically weaker than the PC1CTF since the majority of PC1 in the cell is cleaved.

Mouse inducible cell lines

Supplementary Fig. 14 shows that the F-actin and NKA content and their cellular organization are significantly different in PKD-2 KO compared to DMSO control when grown in both MFKP and glass. PKD-2 KO cells have decreased basal F-actin fibers and most of the F-actin is localized in cell-cell junctions. This indicates that PKD-2 KO is sufficient to not only decrease apical-to-basal fluid flux but also dramatically re-arrange the F-actin and NKA localization.

Supplementary Note 7: Role of hydrostatic pressure gradient ΔP in localization of NKA in normal and diseased human primary cells.

ΔP decreases baso-lateral Na^+/K^+ ATPase localization in MDCK-II cells

Na^+/K^+ ATPase (NKA) has been implicated as the driving force behind trans-epithelial Na^+ transport and vectorial fluid movement in both normal and diseased cells [11]. To understand the negative feedback between active fluid flux and hydraulic pressure gradient (ΔP), we hypothesized that the ΔP disrupts NKA and hence decreases osmotically driven fluid flux. Ouabain chemically blocks (NKA) function in kidney epithelial cells in a dose dependent manner. MDCK-II domes were used to titrate for the right concentration of ouabain. **Supplementary Fig. 9a** shows that the percentage of growing domes decreases with increase in concentration of ouabain from 0.1 mM to 10 mM. Using time-lapsed imaging, dome sizes were quantified before and after addition of ouabain in the media. It takes around 4 hours for the effect of ouabain to kick in and fluid pumping stalls gradually. Based on this titration, cells in MFKP were treated with 1mM of ouabain for 4 hours before recording the PPC, which is also validated in recent literature [12] [13]. **Supplementary Fig. 9b** shows that fluid pumping is diminished in MDCK-II cells treated with 1 mM ouabain. Both J_0 and ΔP* decreased significantly with ouabain treatment (**Supplementary Fig. 9c,d**).

Live cell imaging of Na^+/K^+ ATPase (NKA) using SNAP-tagged MDCK-II cells (See Methods) indicate that, short-term exposure of ΔP ($\approx$30mins) decreases NKA localization on the lateral side by disrupting baso-lateral surface. **Fig. 3a-d** shows that ΔP = 200 Pa decreases NKA localization on the lateral domain. The disruption can be rescued after removal of ΔP (**Supplementary Movie 3**). **Fig. 3c** shows the intensity profile of NKA along the red band on **Fig. 3b**, that shows a confocal image of SNAP-tagged NKA. These intensity traces were calculated for every time point along the experiments and under the three pressure perturbations: ΔP = 0, ΔP = 200 Pa and ΔP = 0 (**Supplementary Fig. 5**). The peak values of the normalized intensity traces were used to quantify expression of lateral NKA shown in **Fig. 3d**. The intensity traces in **Supplementary Fig.5** indicate that NKA localization at the lateral surface changes under ΔP.

ΔP disrupts baso-lateral F-actin cortex in MDCK-II cells

Live cell imaging of GFP-tagged F-actin in MDCK-II cells (see Methods) under the same conditions showed that under ΔP, cells developed high-frequency F-actin invaginations **(Fig. 3 e-g)**. The invaginations have a lifetime of $\approx$15 seconds and eventually diminish (decreasing diameter of F-actin circles) (**Supplementary Movie 4**). Interestingly, the invaginations disappear almost immediately after removal of ΔP. It was previously shown that disruption of cortical F-actin leads to disruption of baso-lateral NKA [main text reference # 15,16]. Therefore, we conclude that ΔP causes disruption of F-actin cortex and a remodeling of NKA localization. Here, we discover a short-term (30 mins) reaction to elevated differential pressure an explanation for the negative feedback loop between pressure and fluid flux.

Na^+/K^+ ATPase localization in human cells under ΔP

Even though the direction of fluid flow in normal human kidney (NHK) and ADPKD cystic cells are different, J decreased with ΔP for all the three types of primary cells. To investigate the role of ΔP on the localization of NKA, a hydrostatic pressure difference of 200 Pa was applied to the basal channel for NHK cells and to the apical channel for ADPKD cells. These cells were then fixed and stained to examine for cytoskeletal organization and NKA localization.

In case of NHKc cells in control conditions (indicated by $\Delta P = 0$), F-actin is cortical in nature and primarily localized in the basal side, forming thick stress fibers (**Supplementary Fig. 15c,d, g**). NKA is enriched in the canonical baso-lateral domain with strong enrichment on lateral domain (**Supplementary Fig. 15c,d, h**). However, upon application of basal hydraulic pressure gradient (indicated by ΔP = 200 Pa), the basal stress fibers disappeared. The F-actin intensity in the cell-cell junctions was thicker in case of stall pressure as compared to control cells, indicating F-actin reinforcement in the junctions with perturbation (**Supplementary Fig. 15k,m,o,q**). The baso-lateral polarization and the enrichment of NKA on the lateral side was same under both control and basal ΔP (**Supplementary Fig. 15k-r**). The total NKA intensity was calculated by randomly choosing 20 cells in the maximum intensity projected view of both control and ΔP^* cells (**Supplementary Fig. 15h,j**). In case of cells exposed to basal ΔP, the total intensity had a wide distribution as compared to the control cells (**Supplementary Fig. 15b,c**). This could be the reason why NKA intensity of individual slices decreased a bit in the lateral domain (**Supplementary Fig. 15p**). However the difference in the average NKA intensity under both conditions was not significant ($p = 0.74$) This is consistent with the mRNA levels of ATP1A1 under both conditions (**Supplementary Fig. 15d**).

NHKm cells had thick basal F-actin stress fibers at $\Delta P = 0$. NKA was polarized on the baso-lateral side as expected (**Supplementary Fig. 16c,d,g,h**). Hydraulic pressure gradient on the basal side (indicated by ΔP = 200 Pa) caused a loss of basal F-actin fibers but the basal-to-apical distribution was still maintained. However, ΔP induced a dramatic depolarization of NKA as the localization changed from baso-lateral to mostly cytoplasmic (**Supplementary Fig. 16e,f,i,j**). This effect becomes more clear when we plotted the total intensity of each slice in the confocal stack against the distance (**Supplementary Fig. 16o,p**). Even though the lateral domain has more NKA as compared to the basal side in case of ΔP but the intensity is lower than that of control cells. As compared to the control, ΔP also diluted the enrichment of NKA on the lateral side (**Supplementary Fig. 16l,n,r**). The F-actin expression in the cell-cell junctions did not change (**Supplementary Fig. 16k,m,q**). The loss of F-actin on the basal side in NHKm cells due to ΔP was similar to that of NHKc cells but the baso-lateral dilution and depolarization of NKA is more dramatic in NHKm cells (**Supplementary Fig. 15s,t and 11s,t**). We compared the total NKA intensity of 20 cells

chosen randomly in the maximum intensity projected view of both control and ΔP cells (**Supplementary Fig. 16h,j**). We found that the total NKA expression decreased with ΔP ($p < 0.0001$) (**Fig. 6a**). Therefore, for NHKm cells, ΔP not only changed the localization of NKA but also decreased the overall expression in the cells, which is consistent with the decrease in mRNA expression of ATP1A1 under similar perturbation (**Fig. 6b**).

Like NHKc and NHKm cells, cystic cells from ADPKD patients also have thick F-actin stress fibers on the basal side. The intensity of F-actin is more on the basal side as compared to the apical side (**Supplementary Fig. 15c,d,g, 11c,d,g, 12c,d,g**). NKA is also polarized in the baso-lateral domain but the total expression is lower than that of NHKc and NHKm cells (**Supplementary Fig. 17c,d,h**). Apical ΔP changed the F-actin organization but not the NKA expression or localization. Unlike NHKc and NHKm where basal ΔP caused a dramatic decrease in basal F-actin stress fibers, in case of ADPKD cells, Apical ΔP induced a small decrease of basal F-actin stress fibers (**Supplementary Fig. 15g,i,o, 11g,i,o, 12g,i,o**). Rather the enrichment of F-actin on the sub-apical domain was observed. Clearly, all three cell types changed their cytoskeletal organization of F-actin when exposed to a hydrostatic pressure difference. NKA localization remained baso-lateral under both conditions (**Supplementary Fig. 17c-f,h,j**). The total intensity was also the same in both conditions ($p = 0.74$) (**Fig. 6c**). The F-actin enrichment in the cell-cell junction and the NKA enrichment on the lateral side of the cells was also the same under both conditions (**Supplementary Fig. 17k-n,q,r**) .

ΔP induced transcriptional changes in human and mouse cells

The effect of long-term ΔP exposure ($\approx$ 10 hours) in changing transcription of key ion channels and pumps responsible for fluid transport (**Fig. 6b,d,e,f**) was also investigated. qPCR data of mRNA levels for key genes for mouse cells with PKD-2 KO (**Fig. 6e,f**) and compared it with human cells (**Fig. 6b,d**). Diseased cells (both mouse and human) dont respond to pressure like normal cells. Particularly, the mRNA levels of ATP1A1 (gene that transcribes for NKA) doesnt change with ΔP (marked with green rectangle). Interestingly, for human NHKm cells and in mouse cells, ATP1A1 levels decreased with ΔP (**Fig. 6b,d**). This data indicates a novel mechanism of sensing ΔP and downstream transcriptional regulation.

Supplementary Figures:

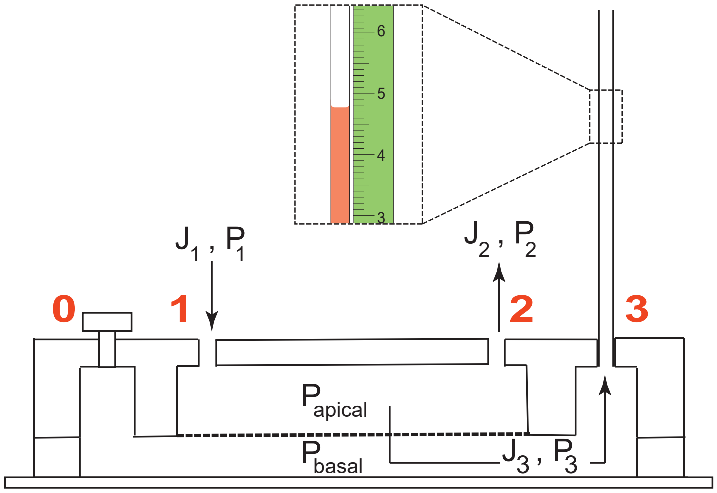

Supplementary Figure 1: Schematic of the longitudinal cross-section of MFKP without cells

Dashed box indicates a zoomed section of the fluid flow in the MC and a mm scale. J_1 and P_1 are the fluid flux and hydrostatic pressure at port 1, J_2 and P_2 are the fluid flux and hydrostatic pressure at port 2, J_3 and P_3 are the fluid flux and hydrostatic pressure at port 3.

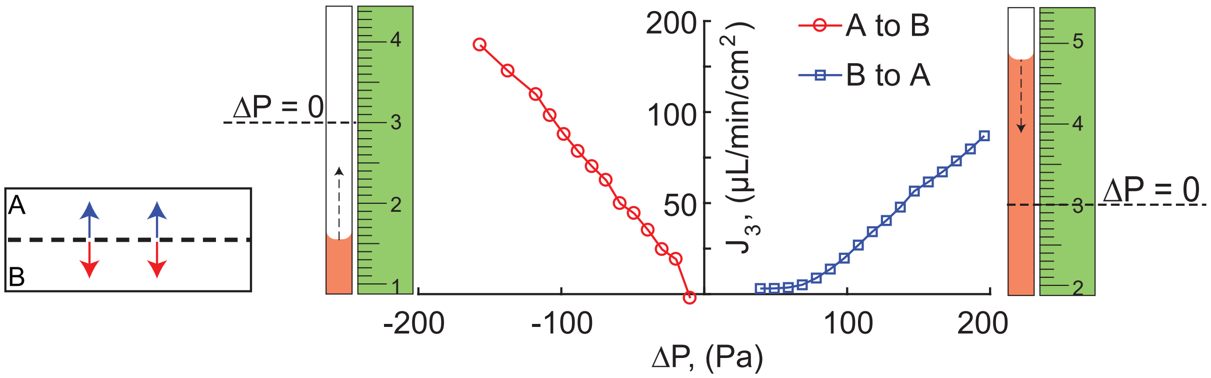

Supplementary Figure 2: Aapico-basal fluid flux J_3 as a function of the trans-membrane hydrostatic pressure gradient ΔP without cells

The flux is in direction of the pressure gradient, and is zero when $P_{\text{basal}} = P_{\text{apical}}$.

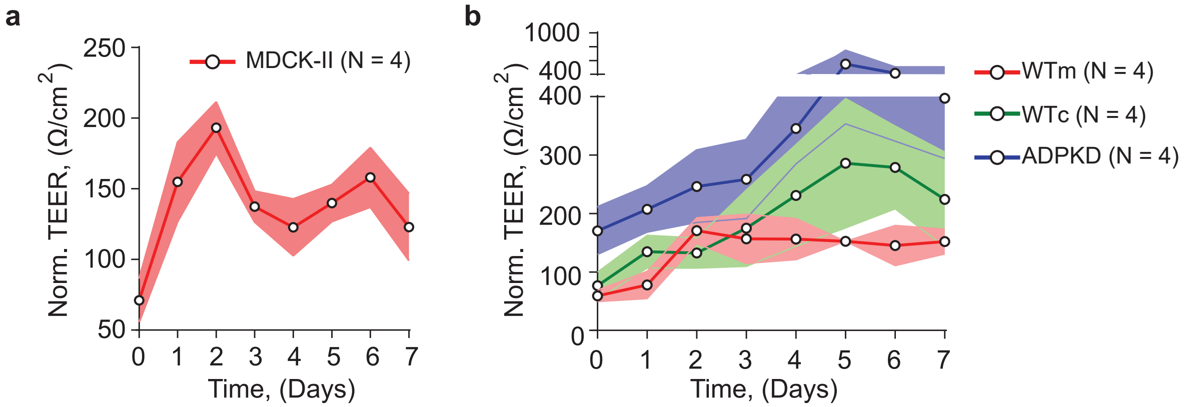

Supplementary Figure 3: Variation of the trans-epithelial electrical resistance (TEER) with the number of days post confluence

(**a**) MDCK II cells, (**b**) NHKc cells, NHKm and ADPKD cells. Data here are presented as mean values ± shaded SEM. N = biological replicates.

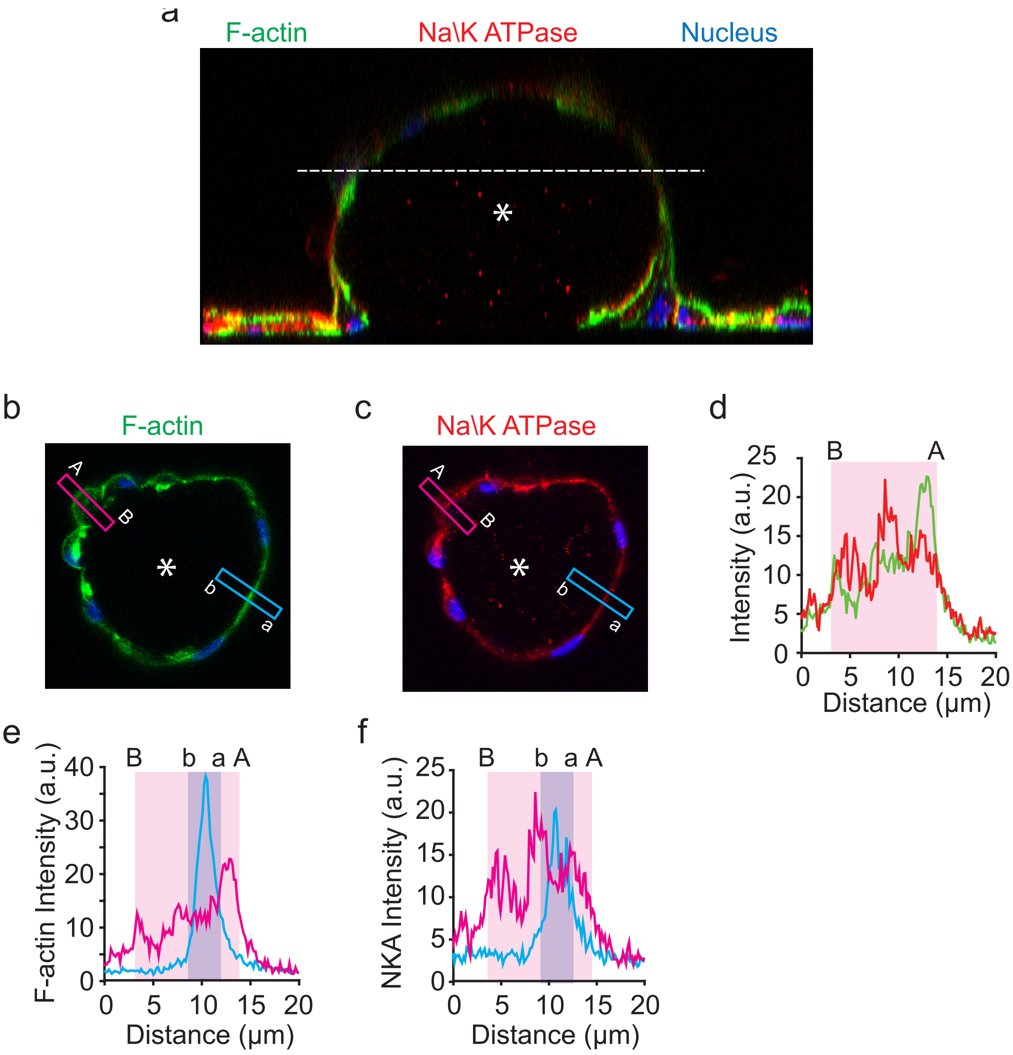

Supplementary Figure 4: Confocal images of MDCK domes

(**a**) 3D confocal reconstruction of a mature MDCK-II dome stained for F-actin (green), NKA (α sub-unit) (red) and DNA (Blue). The asterisk indicates lumen in the dome. (**b**) Cross-sectional IF image of F-actin along the dashed line in **a**. (**c**) Cross-sectional IF image of NKA along the dashed line in **a**. (**d**) Comparison of intensity profiles of F-actin and NKA from basal to apical side of a cell in **b** and **c**. The apical and basal domains of the cell are indicated by A and B. Green line indicates F-actin intensity along the pink band in **b** and red line indicates NKA intensity along the blue band in **c**. (**e**) comparison of basal to apical intensity of F-actin in a stretched and unstretched cell in the dome in **b**. The blue line indicates the F-actin intensity along the blue band in **b**. The pink line indicates the F-actin intensity along the pink band in **b**. (**f**) Comparison of basal to apical intensity of NKA in a stretched and unstretched cell in the dome in **b**. The blue line indicates the NKA intensity along the blue band in **b**. The pink line indicates the NKA intensity along the pink band in **b**. The apical and basal domains of the unstretched cell are indicated by A and B. The apical and basal domains of the stretched cell are indicated by **a** and **b**.

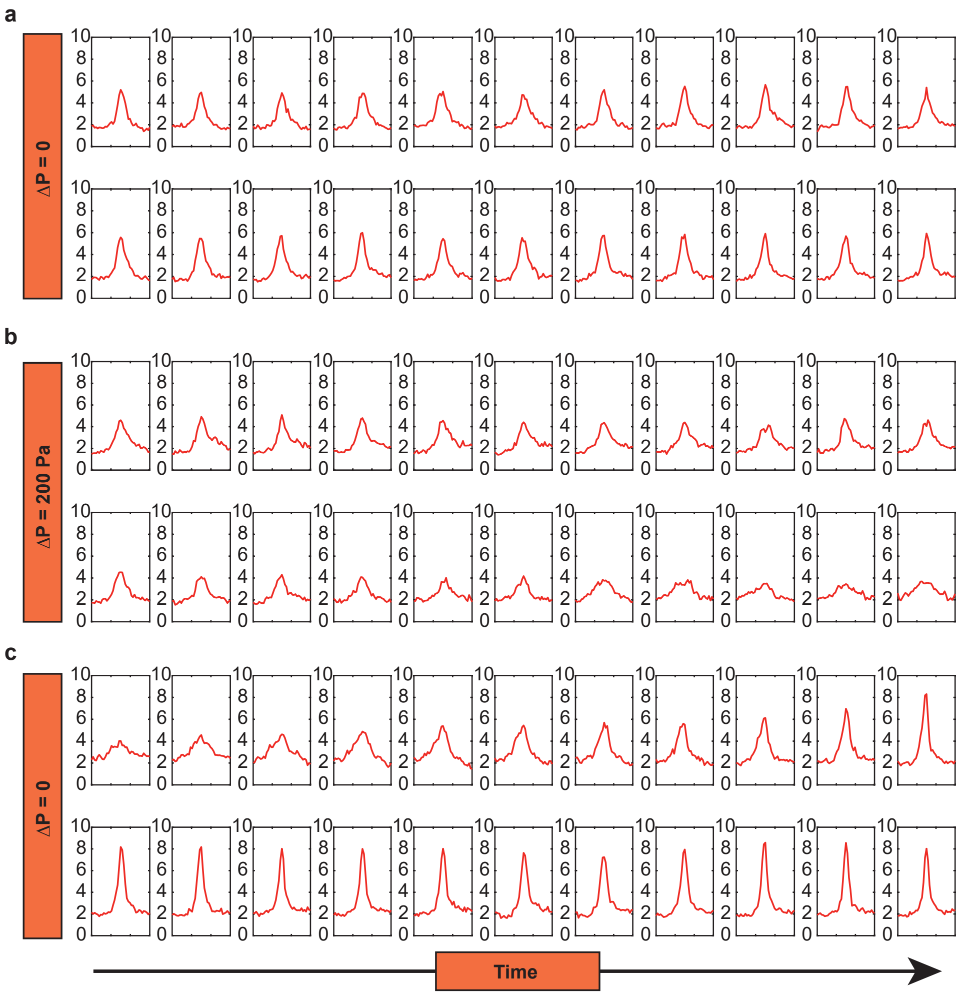

Supplementary Figure 5: SNAP-tagged NKA in MDCK cells

Montage of intensity analysis of a region of interest in SNAP-tagged NKA over time under the following conditions: (**a**) zero pressure gradient $\Delta P = 0$, (**b**) pressure gradient $\Delta P = 200$ Pa, and (**c**) pressure gradient released $\Delta P = 0$. For each condition, there are 22 time points from left to right split into two rows. For each condition, time starts from the first row-first column and ends till second row-last column.

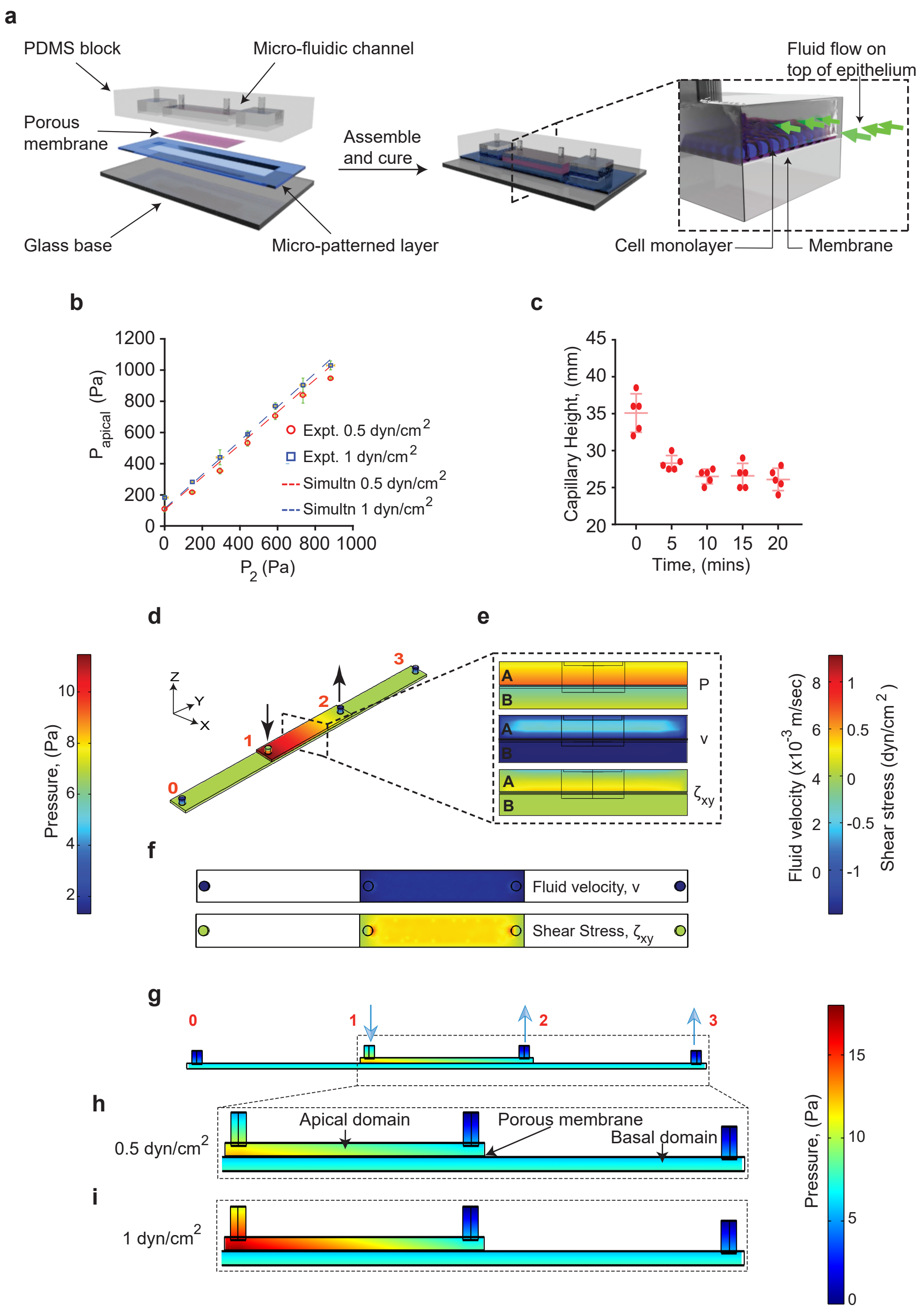
a
PDMS block
Micro-fluidic channel
Porous membrane
Glass base
Micro-patterned layer
Assemble and cure
Fluid flow on top of epithelium
Cell monolayer
Membrane
b
P_apical (Pa)
P_2 (Pa)
Expt. 0.5 dyn/cm2
Expt. 1 dyn/cm2
Simultn 0.5 dyn/cm2
Simultn 1 dyn/cm2
c
Capillary Height, (mm)
Time, (mins)
d
e
Pressure, (Pa)
Fluid velocity (x10-3 m/sec)
Shear stress (dyn/cm2)
f
Fluid velocity, v
Shear Stress, ζxy
g
h
0.5 dyn/cm2
Apical domain
Porous membrane
Basal domain
i
1 dyn/cm2
Pressure, (Pa)

Supplementary Figure 6: Fabrication and calibration of Micro-fluidic Kidney Pump (MFKP)

(**a**) Exploded view of components used in the fabrication and assembly of the Micro-fluidic Kidney Pump (MFKP). The dashed rectangle indicates cross-section of the assembled device with schematic representation of the epithelium grown on the porous membrane. Green arrows indicate fluid flow. (**b**) MFKP was calibrated by measuring the hydrostatic pressure in the apical channel (P_{apical}) while applying fluid shear stress (FSS) on the apical side and changing the hydrostatic pressure in port 2 (P_2). During calibration FSS was applied on top of the porous membrane with no cells by changing the flow rate and P_2 was increased by increasing the height of the fluid reservoir connected to port 2. Horizontal error bars indicate same-device variation (mean $\pm$ standard deviation, n = 3, independent experiments) and vertical error bars indicate device-device variation (mean $\pm$ standard deviation, n = 5, independent devices). Dashed lines indicate values obtained from the FEM simulation under similar velocity and pressure conditions. (**c**) The MC was calibrated using cell culture medium to measure the height of fluid due to capillary action. Data are presented as mean values (center line) $\pm$ standard deviation (error bars). (n = 5, independent devices). (**d**) FEM model indicating heat map of the pressure field in the MFKP under an FSS of 0.5 dyn/cm^2. Arrows indicate directions of fluid flux and the numbers 0,1, 2 and 3 indicate the flow ports. (**e**) Cross-sectional variation of pressure (P), fluid velocity magnitude (v) and FSS (τ_{xy}) in the XZ plane in MFKP. Apical (A) and basal (B) channels are separated by a porous membrane (bold black line). (**f**) Heatmap indicating the variation of fluid velocity (v) and FSS (τ_{xy}) on top of the porous membrane in MFKP along the XZ plane. (**g**) Heatmap indicating uniform pressure profile along the length of the basal channel (YZ plane) for FSS of 0.5 dyn/cm^2 (**h**) and 1 dyn/cm^2. (**i**). The pressure gradient along the apical channel in both FSS is due to the applied flow.

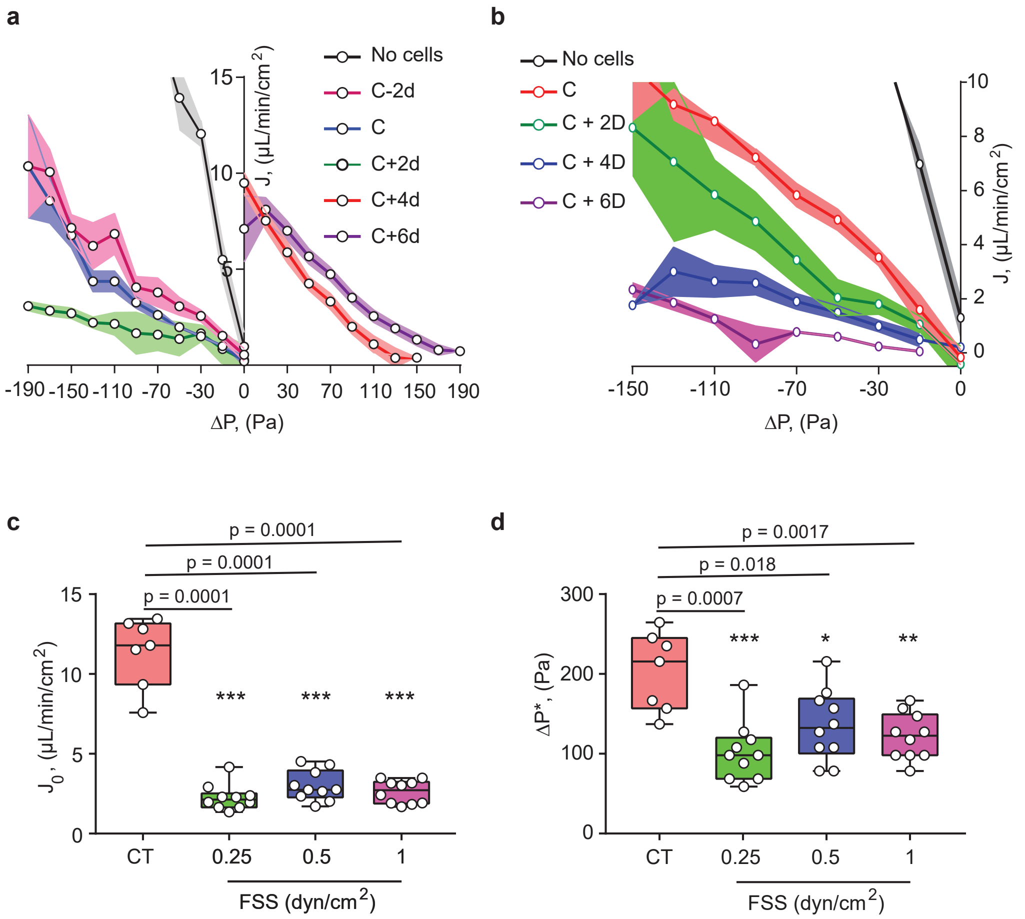

Supplementary Figure 7: Longitudinal characterization of the pumping performance in MFKP and role of fluid shear stress

(**a**) Longitudinal time course experiments with MDCK-II cells in MFKP, demonstrating active fluid pumping performance as a function of monolayer maturation. PPC readouts were taken every two days as cells continued to divide to reach confluence and then formed a strong monolayer with mature tight junctions. Both J_0 and ΔP^* were zero for No cells (black, N =5, independent devices), 2 days before confluence (C-2D, pink, N= 5, biological replicates), confluence (C, blue, N= 6, biological replicates), 2 days after

confluence (C+2D, green, N= 6, , biological replicates). Before confluence, the monolayer is leaky and is not able to develop a pressure differential. However after 4 days post-confluence, i.e. for C+4D (red, n= 10, N = 3, independent devices, biological replicates) and C+6D (purple, n= 11, N = 3, independent devices, biological replicates), positive J_0 and ΔP^* were measured, indicating active fluid pumping and pressure generation by the MDCK-II monolayer. Data are presented as mean values $\pm$ SEM (shaded area). (**b**) Similar longitudinal experiments for mouse fibroblast cells (NIH-3T3) in MFKP. 3T3 cells never developed trans-epithelial fluid flow or pressure differential. No cells (black, N = 5, independent devices), confluence (C, red, N= 6, biological replicates), 2 days after confluence (C+2D, green, N= 3, biological replicates), 4 days post-confluence (C+4D, blue, N= 5, biological replicates) and 6 days post-confluence (C+6D, purple, N= 4, biological replicates). Data are presented as mean values $\pm$ SEM (shaded area). Quantification of J_0 and (**c**) ΔP^* (**d**) for MDCK-II cells in the MFKP with fluid shear stresses varying of 0.25 dyn/cm^2 (n = 10, N = 3, independent device, biological replicates), 0.5 dyn/cm^2 (n = 10, N = 3, independent device, biological replicates) and 1 dyn/cm^2 (n = 10, N = 3, independent device, biological replicates) and compated to control cells (CT, n = 7, N = 3, independent device, biological replicates). For J_0, p-value$_{CT-0.25dyn/cm^2}$ = 0.0001, p-value$_{CT-0.5dyn/cm^2}$ = 0.0001 and p-value$_{CT-1dyn/cm^2}$ = 0.0001. For ΔP^*, p-value$_{CT-0.25dyn/cm^2}$ = 0.0007, p-value$_{CT-0.5dyn/cm^2}$ = 0.018 and p-value$_{CT-1dyn/cm^2}$ = 0.0017. (*p < 0.05 , **p < 0.01, ***p < 0.001, ***p < 0.0001. Unpaired, two-tailed, Mann-Whitney test was conducted for statistical analysis). For the box plots, lower and upper box boundaries 25th and 75th percentiles, respectively, line inside box median, lower and upper error lines 10th and 90th percentiles, filled circles indicate data points respectively.

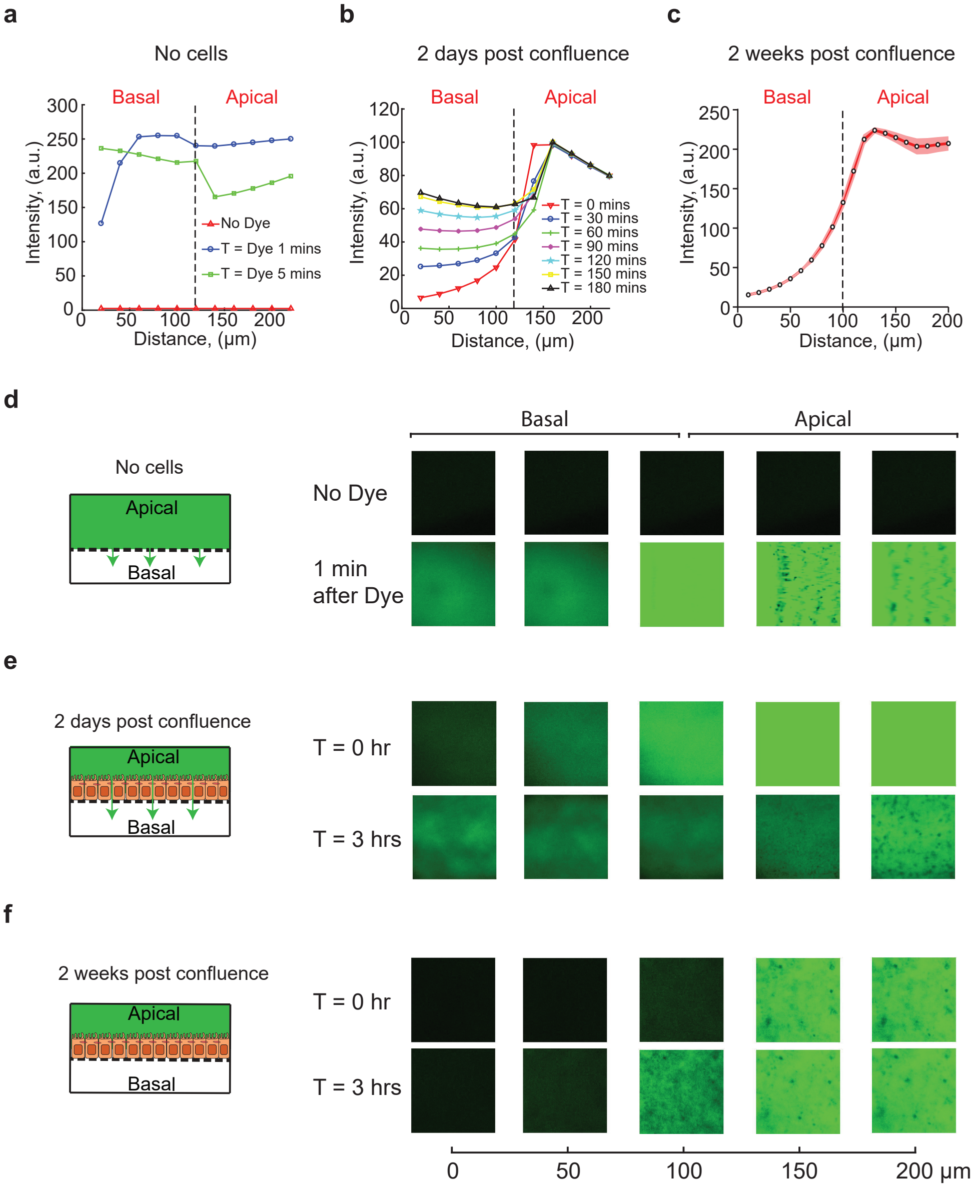

a
No cells
Basal
Apical
Intensity, (a.u.)
Distance, (μm)
No Dye
T = Dye 1 mins
T = Dye 5 mins
b
2 days post confluence
Basal
Apical
Intensity, (a.u.)
Distance, (μm)
T = 0 mins
T = 30 mins
T = 60 mins
T = 90 mins
T = 120 mins
T = 150 mins
T = 180 mins
c
2 weeks post confluence
Basal
Apical
Intensity, (a.u.)
Distance, (μm)
d
No cells
Apical
Basal
No Dye
1 min
after Dye
e
2 days post confluence
Apical
Basal
T = 0 hr
T = 3 hrs
f
2 weeks post confluence
Apical
Basal
T = 0 hr
T = 3 hrs
0
50
100
150
200 μm

Supplementary Figure 8: MDCK-II cells polarize to form strong apical-basal barrier in the MFKP

Confocal fluorescence images of basal and apical channels were taken before and after injection of FITC-conjugated dextran dye of MW 2000 kDa into the apical channel of MFKP in the absence of cells. Average intensity of each slice in the confocal stack were used to quantify the permeability of the porous membrane as well as the barrier strength of the epithelium. (**a**) Without the cells, the intensity of both apical and basal channel is almost zero. However, the average intensity on the basal channel shoots up within a minute of adding the dye to the apical channel. To prevent any leakage the basal channel was closed (by closing ports 0 and 3) before injecting dye into the apical channel. Within 5 minutes the dye diffuses completely into the basal channel. The dashed line indicates the position of the porous membrane that separates the basal and apical channel. (**b**) For MDCK-II epithelium 2 days post confluence, it takes about 3 hours for the dye to diffuse into the basal side from the apical side indicating poor barrier strength. (**c**) For MDCK-II epithelium 2 weeks post confluence, the dye does not diffuse. The average intensity of the slices in the basal channel remained almost same after 3 hours post injection of dye in the apical channel (n=4, independent devices). Data are presented as mean values $\pm$ standard deviation (shaded aread). (**d**) Comparison of fluorescence images of the basal and apical channel in MFKP with and without the dye (green) indicate diffusion of the dye through the porous membrane. The schematic on the left demonstrates experimental condition and green arrows indicate the direction of diffusion of the dye. (**e**) For an MDCK-II epithelium that is 2 days post confluence, representative images of the basal and apical channel in MFKP at T = 0 hour and at T = 3 hours indicate slow diffusion of the dye through the epithelium. (**f**) For an MDCK-II epithelium that is 2 weeks post confluence, representative images of the basal and apical channel in MFKP at T = 0 hour and at T = 3 hours indicate no dye diffusion through the epithelium.

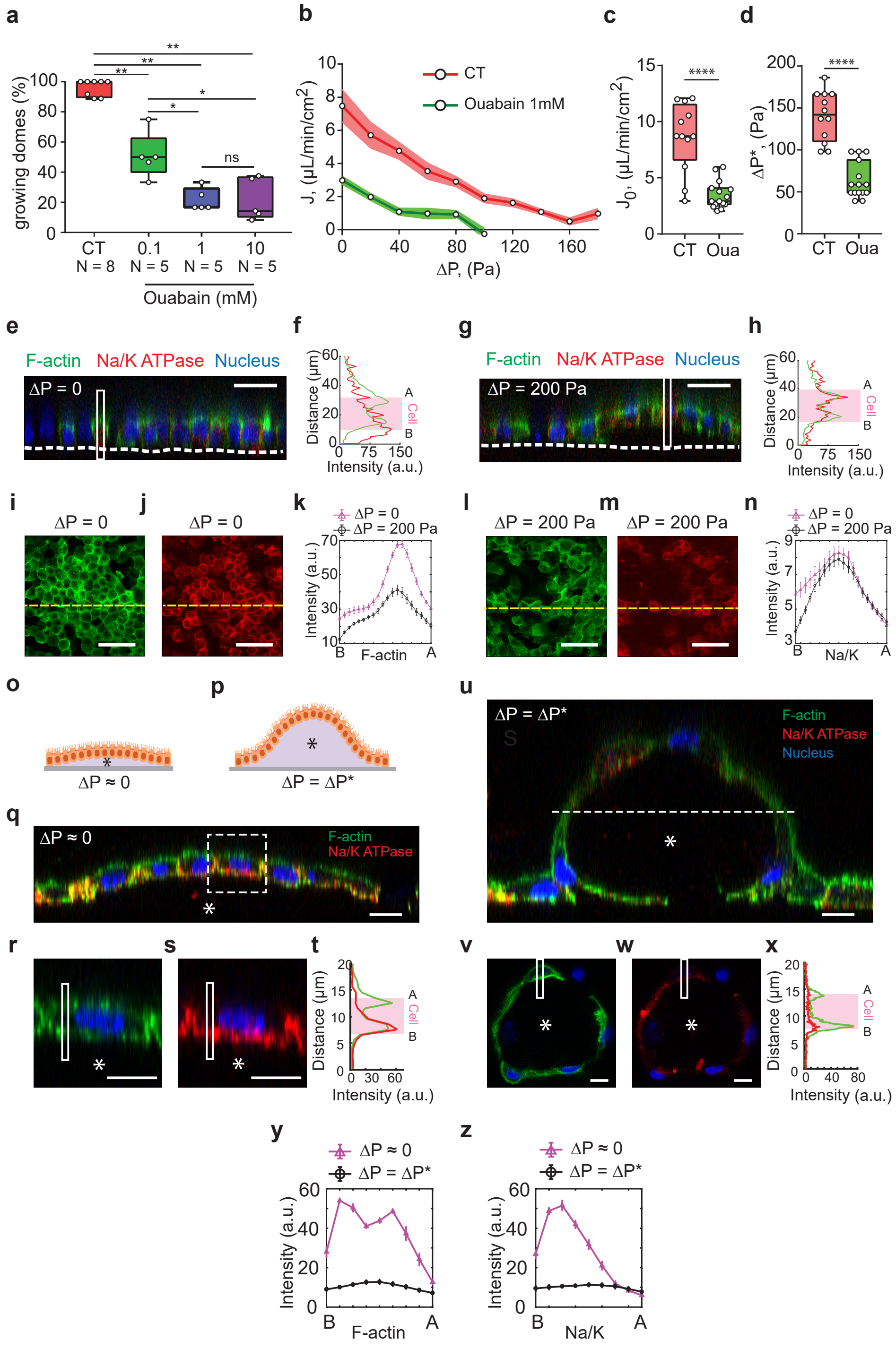

a
growing domes (%)
CT N = 8
0.1 N = 5
1 N = 5
10 N = 5
Ouabain (mM)
b
CT
Ouabain 1mM
ΔP, (Pa)
c
CT Oua
d
CT Oua
e
F-actin Na/K ATPase Nucleus
ΔP = 0
f
Distance (μm)
Intensity (a.u.)
Cell
g
F-actin Na/K ATPase Nucleus
ΔP = 200 Pa
h
Distance (μm)
Intensity (a.u.)
i
ΔP = 0
j
ΔP = 0
k
ΔP = 0
ΔP = 200 Pa
Intensity (a.u.)
F-actin
l
ΔP = 200 Pa
m
ΔP = 200 Pa
n
Na/K
o
ΔP ≈ 0
p
ΔP = ΔP*
q
ΔP ≈ 0
F-actin
Na/K ATPase
u
ΔP = ΔP*
F-actin
Na/K ATPase
Nucleus
r
s
t
Intensity (a.u.)
v
w
x
y
ΔP ≈ 0
ΔP = ΔP*
F-actin
z
Na/K

Supplementary Figure 9: Ouabain decreases fluid pumping performance in MDCK-II cells

MDCK-II cells form fluid filled domes when grown on impermeable supports. The hydrostatic pressure in domes is similar to the stall pressure measured with in MFKP (Fig. 2). These domes were used to study the effect of ouabain (NKA blocker) on the pump performance of MDCK-II cells. Ouabain decreases trans-epithelial fluid pumping by blocking NKA in MDCK-II cells. It takes around 4 hours for ouabain to take effect. (**a**) After 4 hours of treatment, the percentage of growing domes decreases with increase in ouabain concentration from 0.1-10 mM and saturates at 1 mM (n = N = 5,) (n, N, technical repeats, biological repeats) when compared to vehicle control (CT) (n = N = 8). Each data point is the average of n technical repeats for that cell passage number. p-value$_{CT-0.1mM}$ = 0.0016. p-value$_{CT-1mM}$ = 0.0016. p-value$_{CT-10mM}$ = 0.0016. p-value$_{0.1-1mM}$ = 0.0159. p-value$_{0.1-10mM}$ = 0.0317. p-value$_{1-10mM}$ = 0.6905. (*p < 0.05 , **p < 0.01, ns = not significant. Two-tailed Mann-Whitney t-test) (**b**) Pump performance curves of MDCK-II cells with and without 1 mM of Ouabain (CT, vehicle control, n = 12, N = 4 and Ouabain, n = 15, N = 4), n = number of devices, N = biological repeats. Data are presented as mean values $\pm$ SEM (shaded area) (**c-d**) Comparison of J_0 and ΔP^* for MDCK-II epithelium in vehicle control (CT) (n = 12, N = 3, independent device, biological replicates) and 1 mM Ouabain treatment (Oua) (n = 15, N = 3, independent device, biological replicates). (****p < 0.0001. Two-tailed Mann-Whitney t-test). For the box plots, lower and upper box boundaries 25th and 75th percentiles, respectively, line inside box median, lower and upper error lines 10th and 90th percentiles, filled circles indicate data points respectively. (**e**) XZ confocal section of MDCK-II epithelium in MFKP showing colocalization of F-actin (green), NKA (red) at zero hydrostatic pressure gradient (or $\Delta P = 0$). The white dashed line indicates the porous membrane. (**f**) IF intensity profiles of F-actin (green) and NKA (red) along the band in **d** in arbitrary units (a.u.). The pink rectangle indicates location of cell, A and B indicate apical and basal surface of the cell under consideration. (**g**) XZ confocal section of MDCK-II epithelium in MFKP showing colocalization of F-actin (green), NKA (red) in the cells at stall pressure (or ΔP = 200Pa). The white dashed line represents the porous membrane. (**h**) Intensity profiles of Factin (green) and NKA (red) along the band in **f**. (**i**) Maximum intensity projected XY IF image of F-actin at ΔP = 0. (**j**) Maximum intensity projected XY IF image of NKA at ΔP = 0. The yellow dashed lines represent the line of the XZ projection in **d**. (**k**) Comparison of the total intensity of F-actin in five cells chosen arbitrarily along the yellow dashed line is plotted versus Z. B and A indicate the basal and apical surface of the cells. Data are presented as mean values $\pm$ SD (error bars). (**l**) Maximum intensity projected XY IF image of F-actin at ΔP = 200Pa. (**m**) Maximum intensity projected XY IF image of NKA at ΔP = 200Pa. The yellow dashed lines represent the line of the XZ projection in **f**. (**n**) Comparison of the total intensity of NKA in five cells chosen arbitrarily along the yellow dashed line plotted versus Z. B and A indicate the basal and apical surface of the cells. Data are presented as mean values $\pm$ SD (error bars). Scale bar = 50 μm.

Fluid-filled domes on impermeable substrates were used to investigate the effect of stall pressure on the colocalization of F-actin and NKA in MDCK-II cells. (**o**) Schematic representation of an unstable MDCK-II dome, wherein the epithelium has just started to accumulate fluid on the basal side. This situation is $\Delta P \approx 0$. (**p**) Schematic representation of a stable MDCK-II dome, where the hydrostatic pressure gradient approximately equal to the stall pressure (ΔP^*). (**q**) Confocal reconstruction of an unstable dome showed colocalization of F-actin (green) and NKA (red) in the cells. Scale bar = 10μm. (**r**) Zoomed view of a cell indicated by dashed rectangle in **q**, showing enrichment of F-actin in the cortex. (**s**) Zoomed view of the cell indicated by dashed rectangle in **q**, showing enrichment of NKA on the baso-lateral domain. Scale bar = 10μm. (**t**) Intensity profiles of F-actin (green) and NKA (red) along the band in **r** and **s**. The pink

rectangle indicates location of cell with the band and A and B indicate apical and basal domain of the cell under consideration. The asterisk indicates basal side of the cells. (**u**) Confocal reconstruction of a stable dome showing colocalization of F-actin (green) and NKA (red) in the cells. Scale bar = 10μm. (**v**) Cross-sectional view of the dome indicated by dashed line in **u**, showing enrichment of F-actin in the cells. **w**, Cross-sectional view of the dome indicated by dashed line in **u**, showing enrichment of NKA in the cells. Scale bar=10μm. (**x**) Intensity profile of F-actin (green) and NKA (red) along the band in **v** and **w**. (**y**) Comparison of the total intensity of F-actin along the cell height in five cells chosen arbitrarily in the two conditions: unstable domes ($\Delta P \approx 0$) and stable domes ($\Delta P \approx \Delta P^{*}$). (**z**) Comparison of the total intensity of NKA along the cell height in five cells chosen arbitrarily in the two conditions: unstable domes ($\Delta P \approx 0$) and stable domes ($\Delta P \approx \Delta P^{*}$). Purple triangles indicate ($\Delta P \approx 0$) and black circles indicates ($\Delta P \approx \Delta P^{*}$).

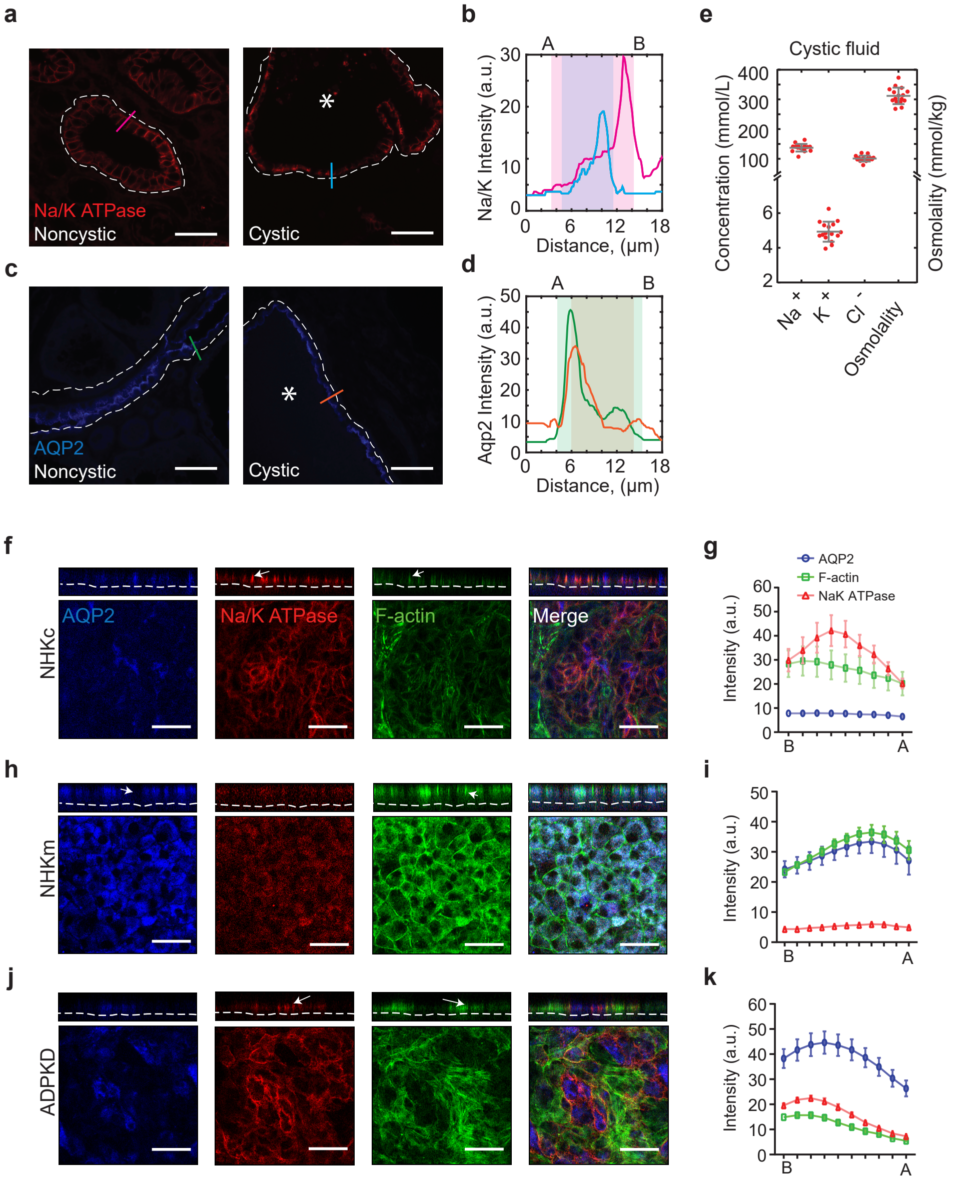

a
Na/K ATPase
Noncystic
Cystic
b
Na/K Intensity (a.u.)
Distance, (μm)
A
B
c
AQP2
Noncystic
Cystic
d
Aqp2 Intensity (a.u.)
Distance, (μm)
e
Cystic fluid
Concentration (mmol/L)
Osmolality (mmol/kg)
Na+
K+
Cl-
Osmolality
f
NHKc
AQP2
Na/K ATPase
F-actin
Merge
g
AQP2
F-actin
NaK ATPase
Intensity (a.u.)
h
NHKm
i
j
ADPKD
k

Supplementary Figure 10: Phenotypic resemblance of both normal (NHK) and ADPKD epithelium grown in MFKP to that of human kidney sections

(**a**) Immunohistochemical analysis of tissue section of normal human kidney and ADPKD kidneys reveal that in case of both normal tubules and cysts, Na/K ATPase (NKA, red) is located primarily on the baso-lateral side. (**b**) Intensity profile of NKA along the lines shown in **a**. The pink line plot is the NKA intensity profile along the pink line in a non-cystic cell. The blue line plot is the NKA intensity profile along the blue line on a cystic cell. A and B mark the apical and basal surface of cells under consideration. (**c**) Aquaporin -2 (AQP2, blue) is however located on the apical or sometimes sub-apical domain of cells in both normal human kidney tissue sections and cystic section in APPKD kidneys. (**d**) Intensity profile of AQP2 along the lines shown in **c**. The green line plot is the AQP2 intensity profile along the green line on a non-cystic cell. The orange line plot is the AQP2 intensity profile along the orange line on a cystic cell. The asterisk indicates the lumen side in cystic tissue section. Both NKA and AQP2 stains indicate that the apico-basal polarity remains the same both in normal tubules and cysts, where the apical side is facing the lumen and the basal side is facing the interstitial. Normal cells demonstrate regular cuboidal columnar morphology. However, the cystic cells have irregular and stretchy morphology coupled with decreased cell height. (**e**) Plot showing the concentration of Na^+, K^+ and Cl^- and osmolality of the cystic fluid from cysts extracted from ADPKD patients. (N = 16, independent biological replicates). Data are presented as mean values $\pm$ SD (error bars). (**f**), (**h**) and (**j**) Confocal immunofluorescence images of normal cortex (NHKc), normal medulla (NHKm) and ADPKD cells extracted from normal human kidneys and ADPKD kidneys seeded in MFKP, showing co-localization of AQP2 (blue), NKA (red) and F-actin (green). The dashed white line in XZ images represents the porous membrane. (**g**) Quantification of apico-basal intensity for AQP2, NKA and F-actin in NHKc epithelium grown on MFKP shows enrichment of NKA primarily in the baso-lateral domain of the cells indicated by white arrow on the XZ image (N = 5, biological repeats). Data are presented as mean values $\pm$ SD (error bars). AQP2 signal was low indicating absence of the protein in NHKc cells. F-actin stress fibers were present in the basal side and strong enrichment in the cell-cell junctions (indicated by arrow) was observed. F-actin XZ images demonstrate that NHKc epithelium exhibit the regular columnar cuboidal epithelium. (**i**) In case of NHKm cells, AQP2 was highly enriched in the apical domain and is also present all over the cytoplasm. NKA intensity was low and is primarily located in the basal side of the cells (N = 5, biological repeats). Data are presented as mean values $\pm$ SD (error bars). The F-actin is fibrous throughout the cytoplasm and strong enrichment in the cell-cell junctions was observed (marked by arrow in the F-actin XZ image. (**k**) ADPKD cells showed AQP2 expression on the apical side in a sporadic fashion in the epithelium (N = 5, biological repeats). Data are presented as mean values $\pm$ SD (error bars). NKA is enriched in the baso-lateral domain and is not uniformly distributed all over the epithelium. F-actin is highly fibrous all over the cytoplasm and unlike normal cells, localization in the cell-cell junction was relatively low. Scale bar = 50μm.

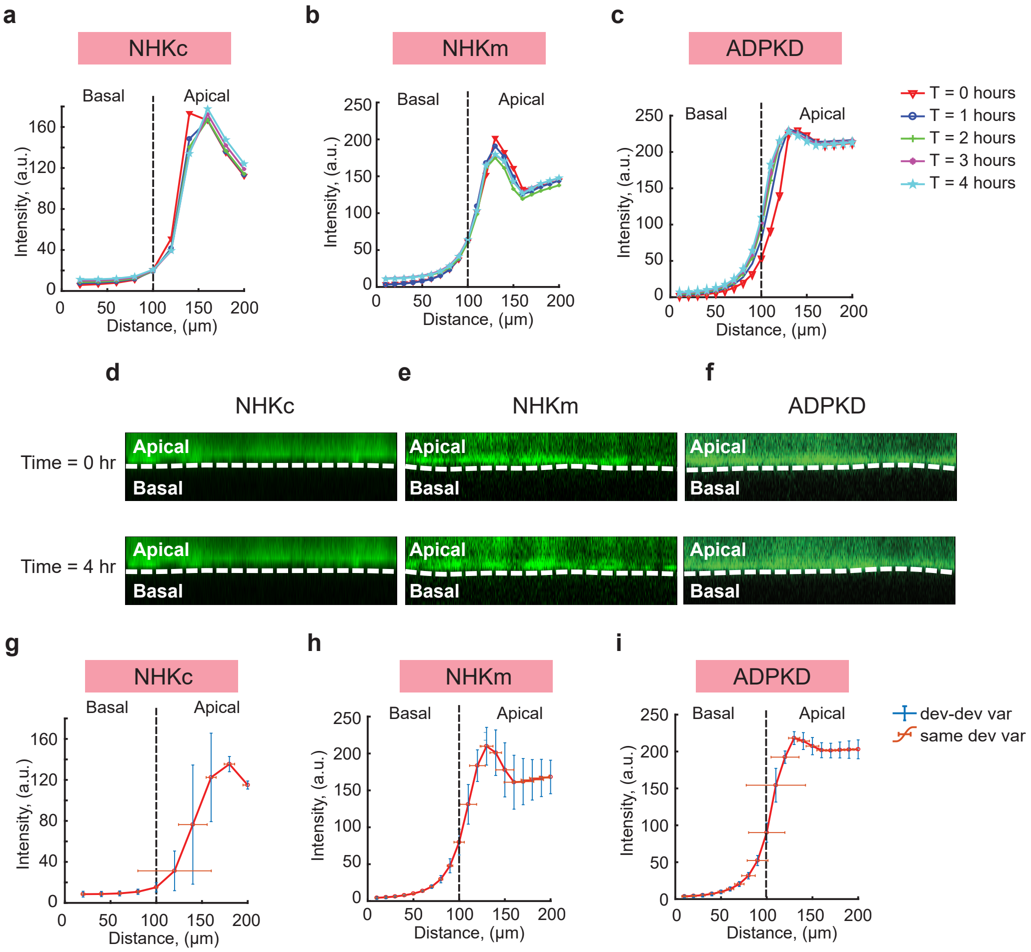
a
NHKc
Basal
Apical
Intensity, (a.u.)
Distance, (μm)
b
NHKm
c
ADPKD
T = 0 hours
T = 1 hours
T = 2 hours
T = 3 hours
T = 4 hours
d
e
f
Time = 0 hr
Time = 4 hr
g
h
i
dev-dev var
same dev var

Supplementary Figure 11: Normal human kidney tubular epithelial and ADPKD cystic cells polarize to form strong apico-basal barrier in the MFKP

Confocal fluorescence images of the basal and the apical channel were taken before and after injection of FITC-conjugated dextran dye of MW 2000 kDa into the apical channel of MFKP in the absence of cells. Average intensity of the stack of images were used to quantify the permeability of the porous membrane as well as the barrier strength of the epithelium. (**a**) For NHKc epithelium after 2 weeks post confluence, the dye did not diffuse from the apical to basal side. The average dye intensity of the basal channel remained the same 4 hours post injection of dye in the apical channel. The vertical dashed line (black) is the position of the porous membrane that separates the basal and apical side. (**b**) For NHKm epithelium after 2 weeks post confluence, the dye did not diffuse from the apical to basal side. The average dye intensity of the basal channel remained the same 4 hours post injection of dye in the apical channel.(**c**) In case of ADPKD epithelium after 2 weeks post confluence, the dye did not diffuse from the apical to basal side. The average dye intensity of the basal channel remained the same 4 hours post injection of dye in the apical channel. Representative fluorescence images of basal and apical channel at T = 0 hour and at T = 4 hours for NHKc (**d**), NHKm (**e**) and ADPKD cells (**f**). The horizontal dashed line (white) is the position of the porous membrane that separates the basal and apical side. The variability of the average intensity values in the same device and from device to device is plotted for NHKc (**g**), NHKm (**h**) and ADPKD cells (**i**). Horizontal error bars indicate same-device variability and vertical error bars indicate device-to-device variability (mean ± standard deviation, N = 3, biological replicates).

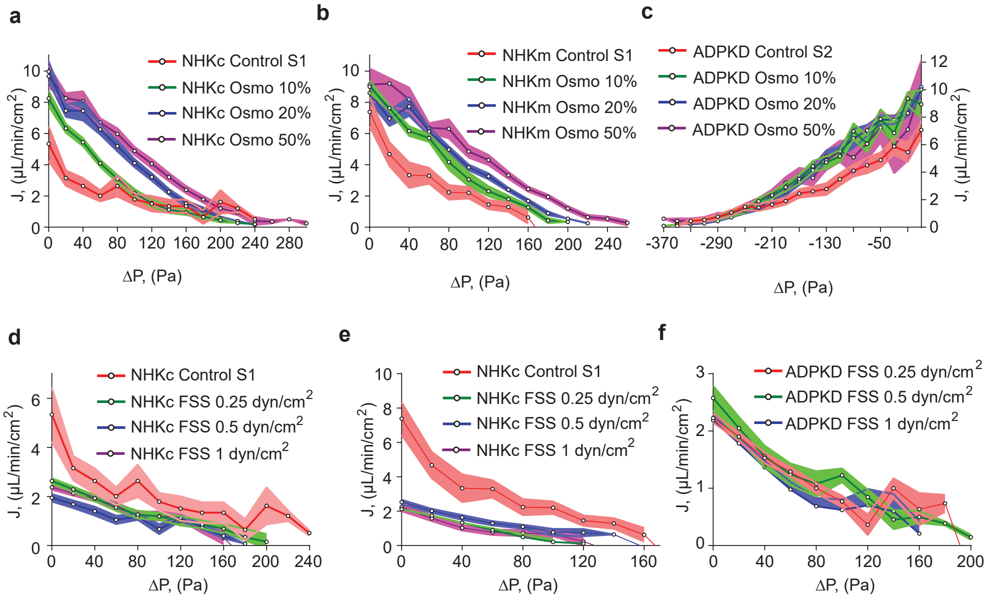

Supplementary Figure 12: Fluid pumping performance curve for NHKc, NHKm and ADPKD cells are dependent on mechanical and osmotic perturbations

(**a**) PPC shows that apical-to-basal trans-epithelial fluid flux (J) for normal human kidney type cortical cells (NHKc) decreases with increase in hydrostatic pressure. Apical hypo-osmotic perturbation (Osmo) of 10% (green, N= 11), 20% (blue, N= 9) and 50% (purple, N= 12) increased both J_0 and ΔP^* compared to control S1 (red, N= 10). Colored bands are SEM throughout. (**b**) PPC shows that apical-to-basal trans-epithelial fluid flux (J) for normal human kidney medulla cells (NHKm) decreases with increase in hydrostatic pressure. Apical hypo-osmotic perturbation (Osmo) of 10% (green, N= 7), 20% (blue, N= 8) and 50% (purple, N= 9) increased both J_0 and ΔP^* compared to control S1 (red, N= 8). (**c**) In case of ADPKD cells, cells pump fluid from the basal to apical channel therefore developing a stall pressure of ΔP^* = -300 Pa. PPC shows that basal-to-apical trans-epithelial fluid flux (J) for human ADPKD cystic cells (ADPKD) decreases with increase in hydrostatic pressure. With basal hypo-osmotic (Osmo) of 10% (green, N= 10), 20% (blue, N= 10) and 50% (purple, N= 8) increased both J_0 and ΔP^* compared to control S2 (red, N= 13). (**d**) In NHKc cells, apical fluid shear stress (FSS) of 0.25 dyn/cm2 (green, N= 12), 0.5 dyn/cm2 (blue, N= 10) and

1 dyn/cm2 (purple, N= 12) increased both J_0 and ΔP^* compared to control S1 (red, N= 10). (**e**) In NHKm cells, apical fluid shear stress (FSS) of 0.25 dyn/cm2 (green, N= 6), 0.5 dyn/cm2 (blue, N= 8) and 1 dyn/cm2 (purple, N= 8) increased both J_0 and ΔP^* compared to control S1 (red, N= 8). (**f**) In ADPKD cells, apical fluid shear stress (FSS) of 0.25 dyn/cm2 (green, N= 15), 0.5 dyn/cm2 (blue, N= 11) and 1 dyn/cm2 (purple, N= 10) reversed the direction of fluid flow and caused a positive ΔP^*.

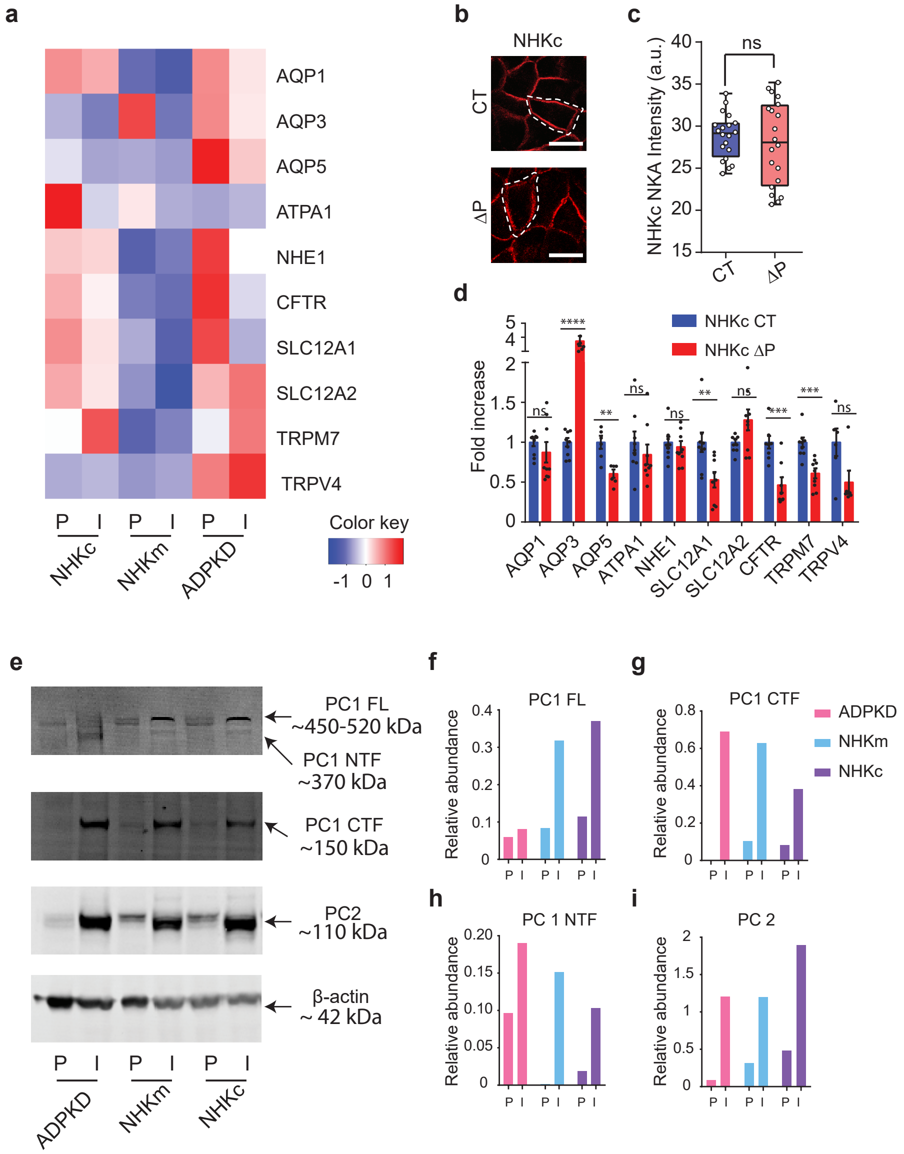
a
AQP1
AQP3
AQP5
ATPA1
NHE1
CFTR
SLC12A1
SLC12A2
TRPM7
TRPV4
P I P I P I
NHKc
NHKm
ADPKD
Color key
-1 0 1
b
NHKc
CT
ΔP
c
NHKc NKA Intensity (a.u.)
ns
CT
ΔP
d
Fold increase
NHKc CT
NHKc ΔP
AQP1
AQP3
AQP5
ATPA1
NHE1
SLC12A1
SLC12A2
CFTR
TRPM7
TRPV4
e
PC1 FL
~450-520 kDa
PC1 NTF
~370 kDa
PC1 CTF
~150 kDa
PC2
~110 kDa
β-actin
~ 42 kDa
P I P I P I
ADPKD
NHKm
NHKc
f
PC1 FL
Relative abundance
g
PC1 CTF
ADPKD
NHKm
NHKc
h
PC 1 NTF
i
PC 2

Supplementary Figure 13: Differential ion channel expression of normal and diseased cells grown in MFKP as compared to impermeable substrates.

(**a**) Heatmaps showing relative expressions of mRNAs extracted from NHKc, NHKm and ADPKD cells grown on permeable substrate (MFKP) and on impermeable substrate (tissue culture treated polystyrene dishes). P and I indicate permeable and impermeable substrate, respectively. The rows are normalized such that the relative concentration across the cells lines has been shown using color code. The list of genes includes Aquaporins (AQP1, AQP3 and AQP5) and ion-pump (NKA) and ion-channels (NHE1, NKCC1, NKCC2, CFTR, TRPM7 and TRPV4). Two biological repeats were done for each condition. (**b**) Immunofluorescence (IF) images of Na/K ATPase (NKA) α-subunit with and without hydrostatic pressure gradient. Control (CT, ΔP = 0) and hydrostatic pressure gradient ΔP = 200 Pa for 5 hours. Scale bars indicate 10 μm. (**c**) Comparison of total NKA intensity under the two conditions (n = 20, N = 3) (n, N, cells, biological repeats) (ns = no significance, p = 0.7381, Unpaired, two-tailed Mann-whiteney t-test was used). For the box plots, lower and upper box boundaries 25th and 75th percentiles, respectively, line inside box median, lower and upper error lines 10th and 90th percentiles, filled circles indicate data points respectively. (**d**) Comparison of relative expression changes of genes in NHKc cells control (CT, $\Delta P = 0$) and under hydrostatic pressure gradient (ΔP = 200 Pa). (n = 9, N = 3, independent experiments, biological replicates). Data are presented as mean values $\pm$ SEM (error bars). (*p_{AQP5} = 0.0152 , **$p_{SLC12A1}$ = 0.0028, **p_{CFTR} = 0.0070, ***p_{TRPM7} = 0.0005, ****$p < 0.0001$. Unpaired two-tailed Mann-Whitney t-test was used for statistical analysis). (**e**) Western blot with an antibody directed against PC1 C-terminus (top panel). Total lysate of cells derived from normal human cortex (NHKc), normal medulla (NHKm) and ADPKD kidneys grown on impermeable and permeable (MFKP) substrates were loaded on the gel. PC1 full length (PC1 FL) and PC1 CTF and PC1 NTF bands are indicated. Below, anti-PC2 western blot shows PC2 band at $\sim$110 kDa. Densitometry of relative protein abundance of each band is represented graphically for PC-1 (**f**), PC 1 FL (**g**), PC1 NTF (**h**), PC2 (**i**).

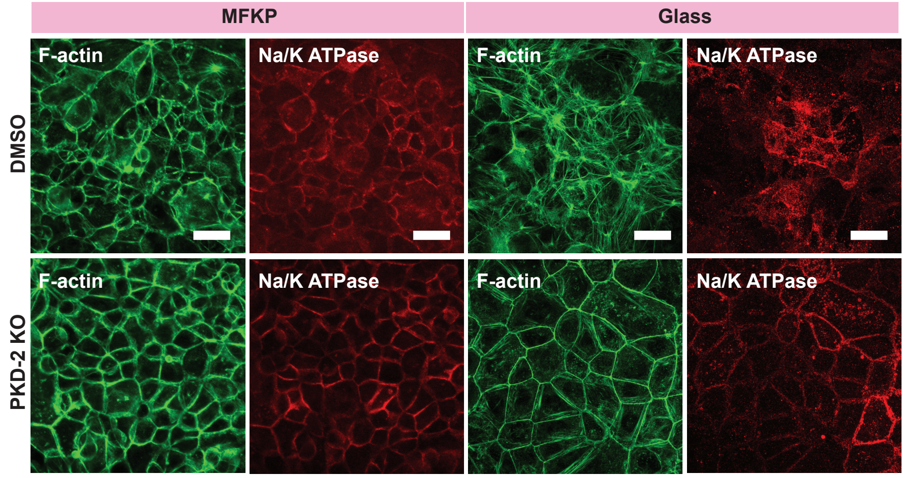

Supplementary Figure 14: PKD-2 KO causes loss of basal F-actin fibers and disrupts NKA localization in mouse cells

Maximum intensity projection of F-actin (green) and NKA (red) for DMSO control and PKD-2 KO in mouse cells were grown in MFKP. Normal cells have basal F-actin stress fibers and overall higher F-actin/cell compared to PKD-2 KO. NKA is also more basal in normal cells. In PKD-2 KO, F-actin is primarily localized at the cell-cell junctions near the apical surface and NKA follows the F-actin distribution. Cell appear to be more dilated. Similar behavior is observed when cells were plated on glass. Scale bar = 10μm.

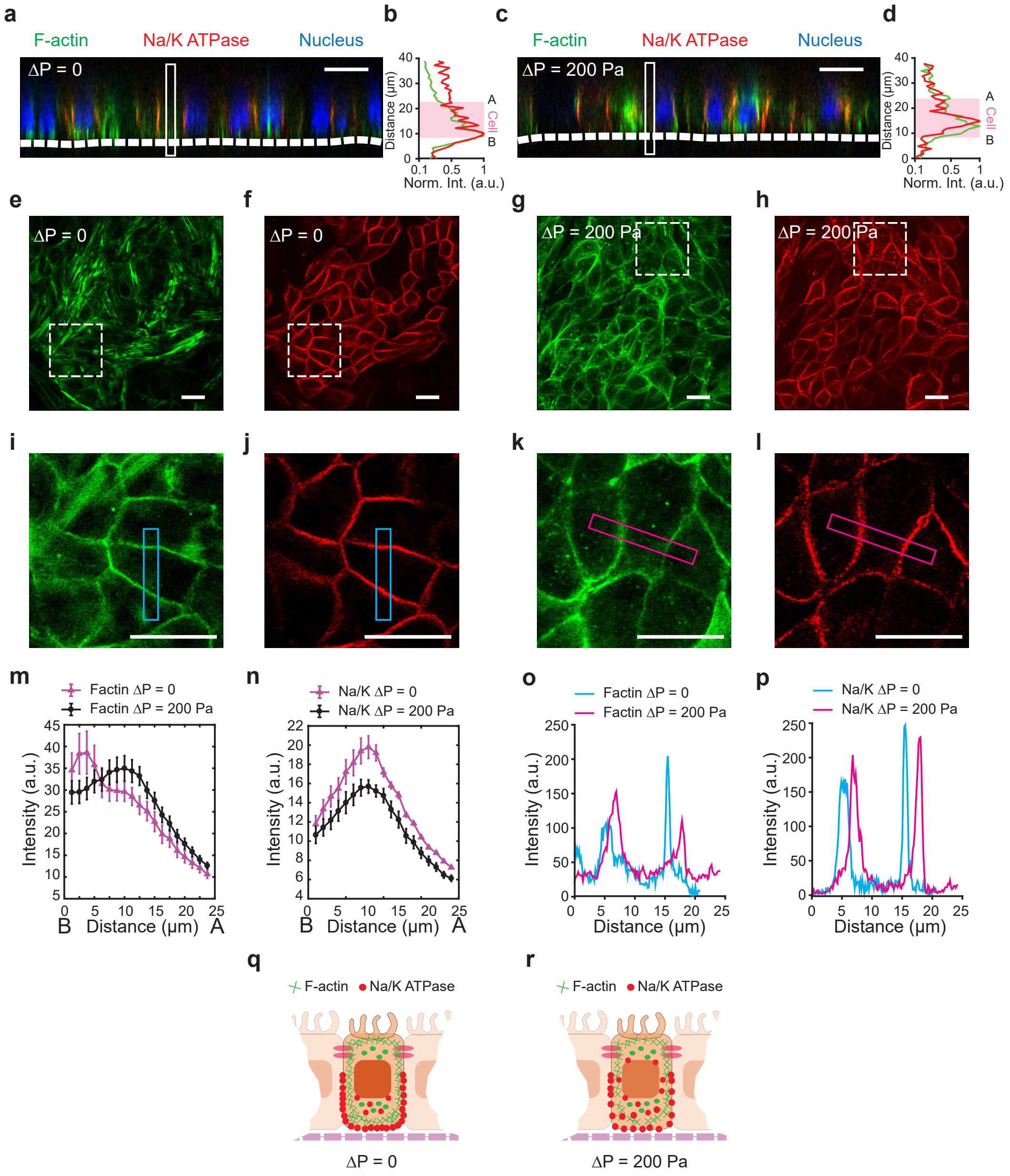

a
F-actin
Na/K ATPase
Nucleus
ΔP = 0
b
Distance (μm)
Norm. Int. (a.u.)
A
Cell
B
c
F-actin
Na/K ATPase
Nucleus
ΔP = 200 Pa
d
Distance (μm)
Norm. Int. (a.u.)
A
Cell
B
e
ΔP = 0
f
ΔP = 0
g
ΔP = 200 Pa
h
ΔP = 200 Pa
i
j
k
l
m
Factin ΔP = 0
Factin ΔP = 200 Pa
Intensity (a.u.)
B Distance (μm) A
n
Na/K ΔP = 0
Na/K ΔP = 200 Pa
Intensity (a.u.)
B Distance (μm) A
o
Factin ΔP = 0
Factin ΔP = 200 Pa
Intensity (a.u.)
Distance (μm)
p
Na/K ΔP = 0
Na/K ΔP = 200 Pa
Intensity (a.u.)
Distance (μm)
q
F-actin
Na/K ATPase
ΔP = 0
r
F-actin
Na/K ATPase
ΔP = 200 Pa

Supplementary Figure 15: Role of stall pressure on NKA in NHKc cells.

(**a**) XZ confocal section of NHKc epithelium in MFKP showing localization of F-actin (green), NKA (red) in the cells at zero trans-epithelial hydrostatic pressure gradient (or ΔP = 0). The white dashed line represents the porous membrane. (**b**) Normalized intensity profiles of F-actin (green) and NKA (red) along the white band in **a**. The pink rectangle indicates location of the cell under consideration. A and B indicate apical and basal domains of the cell under consideration. (**c**) XZ confocal section of NHKc epithelium in MFKP showing localization of F-actin (green), NKA (red) in the cells under pressure gradient ΔP = 200 Pa. (**d**) Normalized intensity profiles of F-actin (green) and NKA (red) along the band in **c**. The pink rectangle indicates location of the cell under consideration in **c**. (**e**) Maximum intensity projection of the 40 μm thick confocal stacks (41 sections at 1 μm intervals) of F-actin at ΔP = 0. (**f**) Maximum intensity projection of the 40 μm thick confocal stacks (41 sections at 1 μm intervals) of NKA (α sub-unit) at ΔP = 0. (**g**) Maximum intensity projection of the 40 μm thick confocal stacks (41 sections at 1 μm intervals) of F-actin at ΔP = 200 Pa. (**h**) Maximum intensity projection of the 40 μm thick confocal stacks (41 sections at 1 μm intervals) of NKA (α sub-unit) at ΔP = 200 Pa. (**i**) Zoomed IF image (represented by the dashed square in **e**) of a single slice showing cortical F-actin in NHKc cells at ΔP = 0. (**j**) Zoomed IF image (represented by the dashed square in **f**) of a single stack showing cortical NKA in NHKc cells at ΔP = 0. (**k**) Zoomed IF image (represented by the dashed square in **g**) of a single slice showing cortical F-actin in NHKc cells at ΔP = 200 Pa. (**l**) Zoomed IF image (represented by the dashed square in **h**) of a single stack showing cortica NKA in NHKc cells at ΔP = 200 Pa. (**m**) Comparison of the total intensity of F-actin in six cells chosen arbitrarily in **e** and **g** is plotted along the height of the cells. Letters B and A in the plot indicate the location of basal and apical surface of cells. Purple triangles indicate ΔP = 0 and black circles indicates ΔP = 200 Pa. Data are presented as mean values $\pm$ SD (error bars). (**n**) Comparison of the total intensity NKA in five cells chosen arbitrarily in **f** and **h** is plotted along the height of the cells. Purple triangles indicate ΔP = 0 and black circles indicates ΔP = 200 Pa. Data are presented as mean values $\pm$ SD (error bars). (**o**) Comparison of the cortical intensities of F-actin at ΔP = 0 and ΔP = 200 Pa. The blue line is the cortical intensity profile of F-actin in case of ΔP = 0 along the band (blue) in **i**. The pink line is the cortical intensity profile of F-actin in case of ΔP = 200 Pa along the band (pink) in **k**. (**p**) Comparison of the cortical intensities of NKA at ΔP = 0 and ΔP = 200 Pa. The blue line is the cortical intensity profile of NKA in case of ΔP = 0 along the band (blue) in **j**. The pink line is the cortical intensity profile of NKA in case of ΔP = 200 Pa along the band (pink) in **l**. (**q**) Schematic representation of the spatial localization of F-actin (green lines) and NKA (red dots) in NHKc cells in MFKP at ΔP = 0. (**r**) Schematic representation of the spatial localization of F-actin (green lines) and NKA (red dots) in NHKc cells in MFKP at ΔP = 200 Pa. Scale bar=20μm.

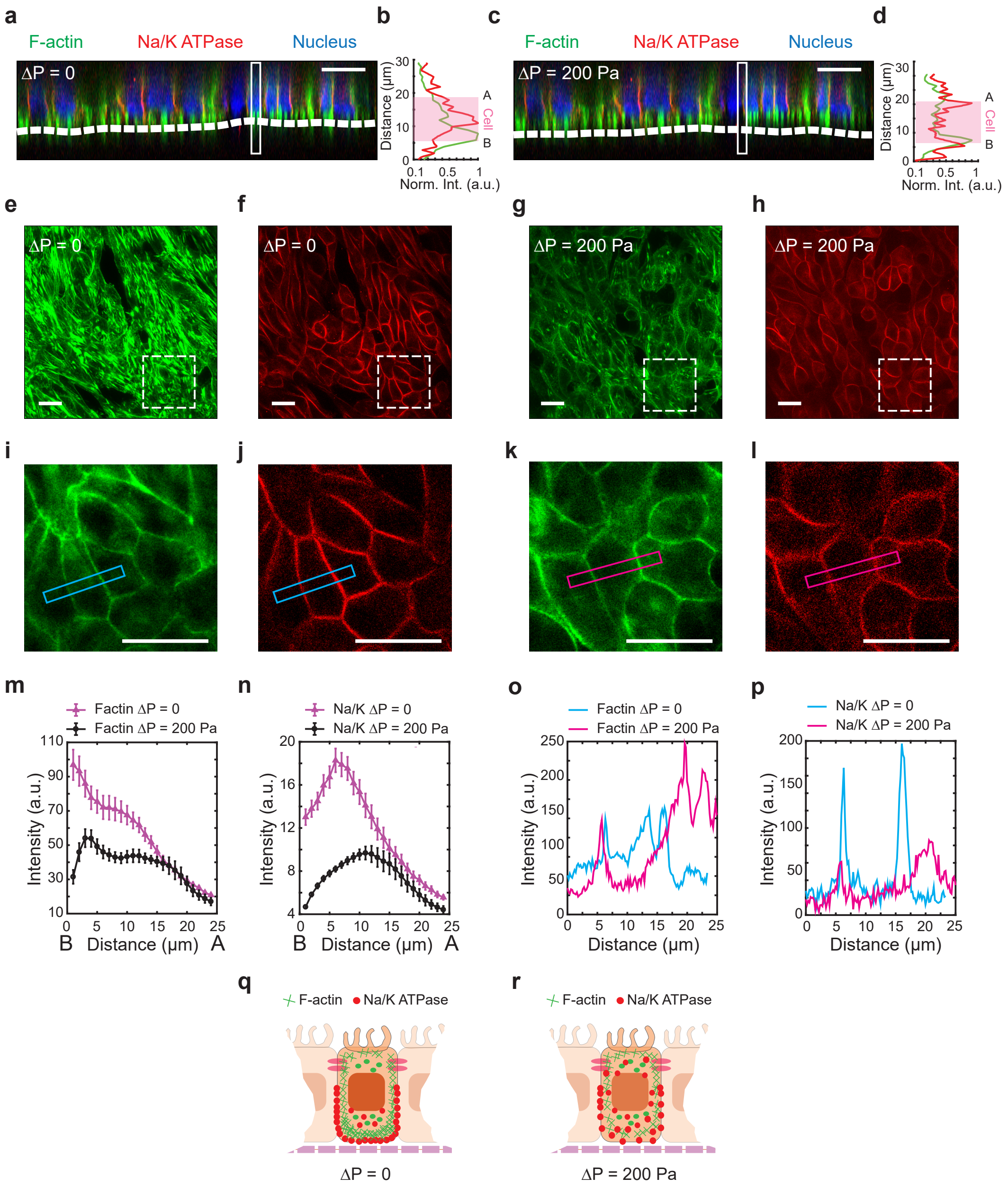

a
F-actin
Na/K ATPase
Nucleus
ΔP = 0
b
Distance (μm)
Norm. Int. (a.u.)
A
Cell
B
c
F-actin
Na/K ATPase
Nucleus
ΔP = 200 Pa
d
Distance (μm)
Norm. Int. (a.u.)
A
Cell
B
e
ΔP = 0
f
ΔP = 0
g
ΔP = 200 Pa
h
ΔP = 200 Pa
i
j
k
l
m
Factin ΔP = 0
Factin ΔP = 200 Pa
Intensity (a.u.)
B Distance (μm) A
n
Na/K ΔP = 0
Na/K ΔP = 200 Pa
Intensity (a.u.)
B Distance (μm) A
o
Factin ΔP = 0
Factin ΔP = 200 Pa
Intensity (a.u.)
Distance (μm)
p
Na/K ΔP = 0
Na/K ΔP = 200 Pa
Intensity (a.u.)
Distance (μm)
q
F-actin
Na/K ATPase
ΔP = 0
r
F-actin
Na/K ATPase
ΔP = 200 Pa

Supplementary Figure 16: Role of stall pressure on NKA in NHKm cells.

(**a**) XZ confocal section of NHKm epithelium in MFKP showing localization of F-actin (green), NKA (red) in the cells at zero trans-epithelial hydrostatic pressure gradient (or ΔP = 0). The white dashed line represents the porous membrane. (**b**) Normalized intensity profiles of F-actin (green) and NKA (red) along the white band in **a**. The pink rectangle indicates location of the cell under consideration. A and B indicate apical and basal domains of the cell under consideration. (**c**) XZ confocal section of NHKm epithelium in MFKP showing localization of F-actin (green), NKA (red) in the cells under pressure gradient ΔP = 200 Pa. (**d**) Normalized intensity profiles of Factin (green) and NKA (red) along the band in **c**. The pink rectangle indicates location of the cell under consideration in **c**. (**e**) Maximum intensity projection of the 40 μm thick confocal stacks (41 sections at 1 μm intervals) of F-actin at ΔP = 0. (**f**) Maximum intensity projection of the 40 μm thick confocal stacks (41 sections at 1 μm intervals) of NKA (α sub-unit) at ΔP = 0. (**g**) Maximum intensity projection of the 40 μm thick confocal stacks (41 sections at 1 μm intervals) of F-actin at ΔP = 200 Pa. (**h**) Maximum intensity projection of the 40 μm thick confocal stacks (41 sections at 1 μm intervals) of Na/K (α sub-unit) at ΔP = 200 Pa. (**i**) Zoomed IF image (represented by the dashed square in **e**) of a single stack showing cortical F-actin in NHKm cells at ΔP = 0. (**j**) Zoomed IF image (represented by the dashed square in **f**) of a single stack showing cortica NKA in NHKm cells at ΔP = 0. (**k**) Zoomed IF image (represented by the dashed square in **g**) of a single stack showing cortical F-actin in NHKm cells at ΔP = 200 Pa. (**l**) Zoomed IF image (represented by the dashed square in **h**) of a single stack showing cortical NKA in NHKm cells at ΔP = 200 Pa. (**m**) Comparison of the total intensity of F-actin in six cells chosen arbitrarily in **e** and **g** is plotted along the height of the cells. Letters B and A in the plot indicate the location of basal and apical surface. Purple triangles indicate ΔP = 0 and black circles indicates ΔP = 200 Pa. Data are presented as mean values $\pm$ SD (error bars). (**n**) Comparison of the total intensity NKA in five cells chosen arbitrarily in **f** and **h** is plotted along the height of the cells. Purple triangles indicate ΔP = 0 and black circles indicates ΔP = 200 Pa. Data are presented as mean values $\pm$ SD (error bars). (**o**) Comparison of the cortical intensities of F-actin at ΔP = 0 and ΔP = 200 Pa. The blue line is the cortical intensity profile of F-actin in case of ΔP = 0 along the band (blue) in **i**. The pink line is the cortical intensity profile of F-actin in case of ΔP = 200 Pa along the band (pink) in **k**. (**p**) Comparison of the cortical intensities of NKA at ΔP = 0 and ΔP = 200 Pa. The blue line is the cortical intensity profile of NKA in case of ΔP = 0 along the band (blue) in **j**. The pink line is the cortical intensity profile of NKA in case of ΔP = 200 Pa along the band (pink) in **l**. (**q**), Schematic representation of spatial localization of F-actin (green lines) and NKA (red dots) in NHKm cells in MFKP at ΔP = 0. (**r**) Schematic representation of the spatial arrangement of F-actin (green lines) and NKA (red dots) in NHKm cells in MFKP at ΔP = 200 Pa. Scale bar=20μm.

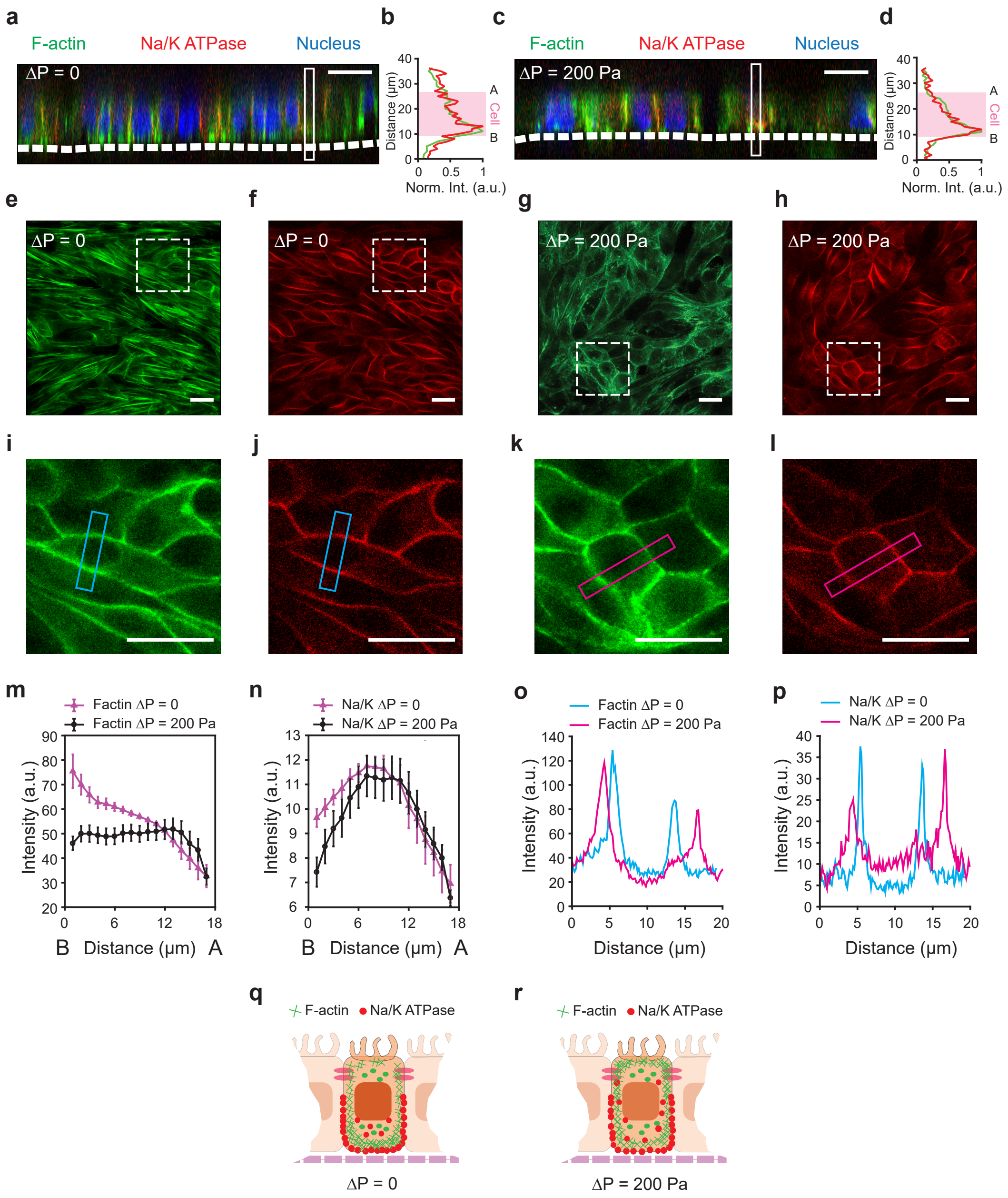

a
F-actin
Na/K ATPase
Nucleus
ΔP = 0
b
Distance (μm)
Norm. Int. (a.u.)
A
Cell
B
c
F-actin
Na/K ATPase
Nucleus
ΔP = 200 Pa
d
Distance (μm)
Norm. Int. (a.u.)
A
Cell
B
e
ΔP = 0
f
ΔP = 0
g
ΔP = 200 Pa
h
ΔP = 200 Pa
i
j
k
l
m
Factin ΔP = 0
Factin ΔP = 200 Pa
Intensity (a.u.)
B Distance (μm) A
n
Na/K ΔP = 0
Na/K ΔP = 200 Pa
Intensity (a.u.)
B Distance (μm) A
o
Factin ΔP = 0
Factin ΔP = 200 Pa
Intensity (a.u.)
Distance (μm)
p
Na/K ΔP = 0
Na/K ΔP = 200 Pa
Intensity (a.u.)
Distance (μm)
q
F-actin
Na/K ATPase
ΔP = 0
r
F-actin
Na/K ATPase
ΔP = 200 Pa

Supplementary Figure 17: Role of stall pressure on NKA in ADPKD cells.

(**a**) XZ confocal section of ADPKD epithelium in MFKP showing colocalization of F-actin (green), NKA (red) in the cells at zero trans-epithelial hydrostatic pressure gradient (or ΔP = 0). The white dashed line represents the porous membrane. (**b**) Normalized intensity profiles of F-actin (green) and NKA (red) along the white band in **a**. The pink rectangle indicates location of the cell under consideration. A and B indicate apical and basal domains of the cell under consideration. (**c**) XZ confocal section of ADPKD epithelium in MFKP showing localization of F-actin (green), NKA (red) in the cells under pressure gradient ΔP = 200 Pa. (**d**) Normalized intensity profiles of F-actin (green) and NKA (red) along the band in **c**. The pink rectangle indicates location of the cell under consideration. (**e**) Maximum intensity projection of the 40 μm thick confocal stacks (41 sections at 1 μm intervals) of F-actin at ΔP = 0. (**f**) Maximum intensity projection of the 40 μm thick confocal stacks (41 sections at 1μm intervals) of NKA (α sub-unit) at ΔP = 0. (**g**) Maximum intensity projection of the 40 μm thick confocal stacks (41 sections at 1 μm intervals) of F-actin at ΔP = 200 Pa. (**h**) Maximum intensity projection of the 40 μm thick confocal stacks (41 sections at 1μm intervals) of Na/K (α sub-unit) at ΔP = 200 Pa. (**i**) Zoomed IF image (represented by the dashed square in **e**) of a single stack showing cortical F-actin in ADPKD cells at ΔP = 0. (**j**) Zoomed IF image (represented by the dashed square in **f**) of a single stack showing cortical NKA in NHKc cells at ΔP = 0. (**k**) Zoomed IF image (represented by the dashed square in **g**) of a single stack showing cortical F-actin in ADPKD cells at ΔP = 200 Pa. (**l**) Zoomed IF image (represented by the dashed square in **h**) of a single stack showing cortica NKA in ADPKD cells at ΔP = 200 Pa. (**m**) Comparison of the total intensity of F-actin in six cells chosen arbitrarily in **e** and **g** is plotted along the height of the cells. B and A indicate the location of basal and apical surfaces of cells. Purple triangles indicate ΔP = 0 and black circles indicates ΔP = 200 Pa. Data are presented as mean values $\pm$ SD (error bars). (**n**) Comparison of the total intensity NKA in five cells chosen arbitrarily in **f** and **h** is plotted along the height of the cells. Purple triangles indicate ΔP = 0 and black circles indicates ΔP = 200 Pa. Data are presented as mean values $\pm$ SD (error bars). (**o**) Comparison of the cortical intensities of F-actin at ΔP = 0 and $\Delta P = \Delta P^*$. The blue line is the cortical intensity profile of F-actin in case of ΔP = 0 along the band (blue) in **i**. The pink line is the cortical intensity profile of F-actin in case of ΔP = 200 Pa along the band (pink) in **k**. (**p**) Comparison of the cortical intensities of NKA at ΔP = 0 and ΔP = 200 Pa. The blue line is the cortical intensity profile of NKA in case of ΔP = 0 along the band (blue) in **j**. The pink line is the cortical intensity profile of NKA in case of $\Delta P = \Delta P^*$ along the band (pink) in **l**. (**q**) Schematic representation of the localization of F-actin (green lines) and NKA (red dots) in ADPKD cells in MFKP at ΔP = 0. **r**, Schematic representation of the localization of F-actin (green lines) and NKA (red dots) in ADPKD cells in MFKP at ΔP = 200 Pa. Scale bar=20μm.

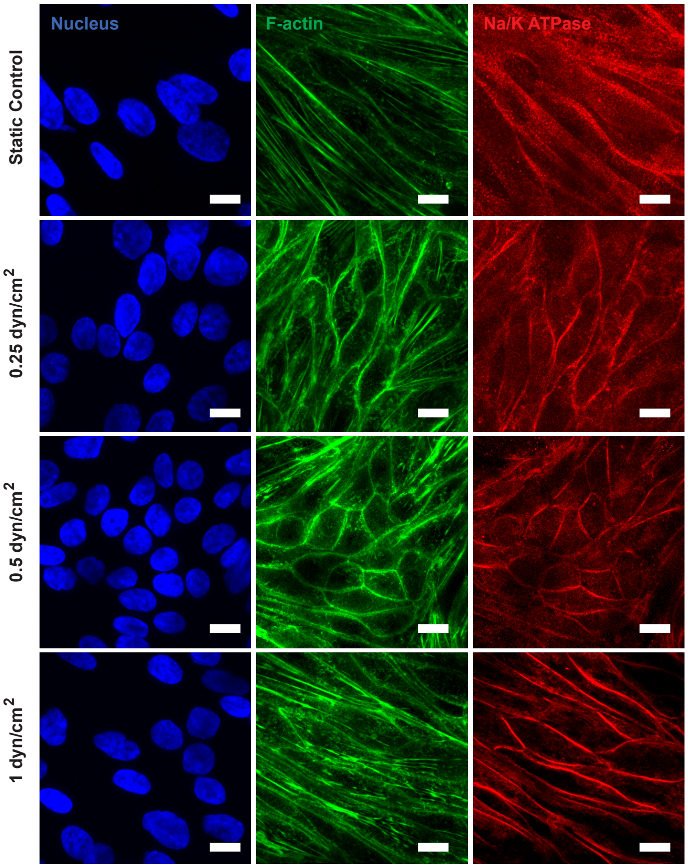

Supplementary Figure 18: Fluid shear stress increases baso-lateral localization of Na/K ATPase in human ADPKD cells

Human ADPKD cystic cells were exposed to apical fluid shear stresses (FSS) for 4 hours and fixed and stained for nucleus, F-actin and Na/K ATPase (NKA) α-subunit. All images are maximum intensity projections of confocal stacks taken from basal to the apical side (35 sections at 1 μm intervals). Compared to static control, FSS of 0.25, 0.5 and 1 dyn/cm^2 increased baso-lateral localizaton of NKA. FSS of 1 dyn/cm^2 caused the maximum polarization. FSS also induced reorganization of F-actin compared to static control. Scale bar = 10 μm.

Supplementary References

[1] Lever, J. E., Inducers of mammalian cell differentiation stimulate dome formation in a differentiated kidney epithelial cell line (MDCK), Proceedings of the National Academy of Sciences 76, 3, 1323-1327, (1979).

[2] Yang, J., Duan, X., Fraser, A., Ewald, A. and Sun, S.X., A microscale pressure sensor based on immiscible fluid/fluid interface. Manuscript submitted for publication, (2019).

[3] Maschmeyer, Ilka, et al. A four-organ-chip for interconnected long-term co-culture of human intestine, liver, skin and kidney equivalents, Lab on a Chip 15.12, 2688-2699, (2015).

[4] Latorre, Ernest, et al. Active superelasticity in three-dimensional epithelia of controlled shape, Nature 563, 7730, 203 (2018).

[5] Weinstein, A. M., A mathematical model of rat proximal tubule and loop of Henle. American Journal of Physiology-Renal Physiology, 308(10), F1076-F1097, (2015).

[6] Jiang, H. and Sun, S.X. Cellular pressure and volume regulation and implications for cell mechanics. Biophysical Journal. 105, 609-619 (2013).

[7] Grantham, Jared J., Arlene B. Chapman, and Vicente E. Torres. ”Volume progression in autosomal dominant polycystic kidney disease: the major factor determining clinical outcomes.” Clinical journal of the american society of Nephrology 1.1 (2006): 148-157.

[8] Rohatgi, Rajeev, et al. ”Cyst fluid composition in human autosomal recessive polycystic kidney disease.” Pediatric Nephrology 20.4 (2005): 552-553.

[9] Hopp, Katharina, et al. Functional polycystin-1 dosage governs autosomal dominant polycystic kidney disease severity, The Journal of clinical investigation 122.11, 4257-4273, (2012).

[10] Thivierge, Caroline, et al., Overexpression of PKD1 causes polycystic kidney disease. Molecular and cellular biology 26.4 (2006): 1538-1548.

[11] Terryn, Sara, et al., Fluid transport and cystogenesis in autosomal dominant polycystic kidney disease. Biochimica et Biophysica Acta (BBA)-Molecular Basis of Disease 1812.10, 1314-1321, (2011).

[12] Valente, Raphael C., et al., ”Diverse actions of ouabain and its aglycone ouabagenin in renal cells.” Cell biology and toxicology 26.3 (2010): 201-213.

[13] Nguyen, Anh-Nguyet T., et al., ”Ouabain binds with high affinity to the na, K-ATPase in human polycystic kidney cells and induces extracellular Signal?Regulated kinase activation and cell proliferation.” Journal of the American Society of Nephrology 18.1 (2007): 46-57.
