# Supplementary material for: Kidney epithelial cells are active mechano-biological fluid pumps
Source: Nat Commun. 2022 Apr 28;13:2317. doi: 10.1038/s41467-022-29988-w (PMC9050750; doi:10.1038/s41467-022-29988-w)
Supplement: Supplementary file 2 — Reporting Summary [file 41467_2022_29988_MOESM2_ESM.pdf]

nature research

Corresponding author(s): *Double-blind peer review submissions: write DBPR and your manuscript number here instead of author names.*

Last updated by author(s): *YYYY-MM-DD*

Reporting Summary

Nature Research wishes to improve the reproducibility of the work that we publish. This form provides structure for consistency and transparency in reporting. For further information on Nature Research policies, see Authors & Referees and the Editorial Policy Checklist .

Statistics

For all statistical analyses, confirm that the following items are present in the figure legend, table legend, main text, or Methods section.

n/a	Confirmed	
☐	☒	The exact sample size (*n*) for each experimental group/condition, given as a discrete number and unit of measurement
☐	☒	A statement on whether measurements were taken from distinct samples or whether the same sample was measured repeatedly
☐	☒	The statistical test(s) used AND whether they are one- or two-sided<br>*Only common tests should be described solely by name; describe more complex techniques in the Methods section.*
☒	☐	A description of all covariates tested
☒	☐	A description of any assumptions or corrections, such as tests of normality and adjustment for multiple comparisons
☐	☒	A full description of the statistical parameters including central tendency (e.g. means) or other basic estimates (e.g. regression coefficient) AND variation (e.g. standard deviation) or associated estimates of uncertainty (e.g. confidence intervals)
☐	☒	For null hypothesis testing, the test statistic (e.g. *F*, *t*, *r*) with confidence intervals, effect sizes, degrees of freedom and *P* value noted<br>*Give P values as exact values whenever suitable.*
☒	☐	For Bayesian analysis, information on the choice of priors and Markov chain Monte Carlo settings
☒	☐	For hierarchical and complex designs, identification of the appropriate level for tests and full reporting of outcomes
☒	☐	Estimates of effect sizes (e.g. Cohen's *d*, Pearson's *r*), indicating how they were calculated

Our web collection on statistics for biologists contains articles on many of the points above.

Software and code

Policy information about availability of computer code

Data collection	Matlab (R2017b) was used to perform computational modeling. ImageJ (version 1.41) was used to process images.
Data analysis	Statistical tests were performed using GraphPad Prism7.

For manuscripts utilizing custom algorithms or software that are central to the research but not yet described in published literature, software must be made available to editors/reviewers. We strongly encourage code deposition in a community repository (e.g. GitHub). See the Nature Research guidelines for submitting code & software for further information.

Data

Policy information about availability of data

All manuscripts must include a data availability statement. This statement should provide the following information, where applicable:

- Accession codes, unique identifiers, or web links for publicly available datasets
- A list of figures that have associated raw data
- A description of any restrictions on data availability

The main data supporting the results of this study are available within the Article and its Supplementary Information. Source data for the Main Figures and Supplementary figures in this study are available in the Source Data File.

Field-specific reporting

Please select the one below that is the best fit for your research. If you are not sure, read the appropriate sections before making your selection.

☒ Life sciences ☐ Behavioural & social sciences ☐ Ecological, evolutionary & environmental sciences

Life sciences study design

All studies must disclose on these points even when the disclosure is negative.

Sample size	Sample size was chosen based on throughput of technique used. Sample sizes were sufficient to show the same trends between the three (at least) or more (sometimes up to 14) replicates performed for each experiment. Additionally, sample sizes were approved by the reviewers.
Data exclusions	Data were not excluded from analysis.
Replication	Each experiment was repeated 3 or more times, with similar results observed each time.
Randomization	Cells are randomized into devices/wells based on technique used. Devices are randomized into drug treatment groups, and treatments are independently administered to each devices/well.
Blinding	No blinding was done because no human subjects were involved.

Reporting for specific materials, systems and methods

We require information from authors about some types of materials, experimental systems and methods used in many studies. Here, indicate whether each material, system or method listed is relevant to your study. If you are not sure if a list item applies to your research, read the appropriate section before selecting a response.

Materials & experimental systems

n/a	Involved in the study	
☐	☒	Antibodies
☐	☒	Eukaryotic cell lines
☒	☐	Palaeontology
☒	☐	Animals and other organisms
☒	☐	Human research participants
☒	☐	Clinical data

Methods

n/a	Involved in the study	
☒	☐	ChIP-seq
☒	☐	Flow cytometry
☒	☐	MRI-based neuroimaging

Antibodies

Antibodies used

For immunohistochemical analysis: Primary antibodies were administered at manufacturer recommended concentration: chicken anti-AQP2 (a gift from Dr. James B. Wade, University of Maryland School, Baltimore, MD, 1:600), mouse anti-Na+/K+ ATPase α-subunit (Clone C464.6, Millipore, 05-369, Lot # 3061212, 1:200). The secondary antibodies used were: goat anti-chicken secondary antibody (Invitrogen, A-21449, 1:200) and goat anti-mouse secondary antibody (Invitrogen, A-11001, 1:200).

For immunocytochemical analysis: Primary antibodies were administered at manufacturer recommended concentration: rabbit anti-AQP2 (Clone P41181, Invitrogen, PA538004, Lot # SI2454403, 1:200), mouse anti-Na+/K+ ATPase α-subunit (Clone C464.6, Millipore, 05-369, Lot # 3061212, 1:200), rat ZO-1 (Clone R40.76, Santa Cruz, SC-33725, Lot # B1518 and # C0818, 1:200) and rabbit anti-E-Cadherin (24e10, Cell Signaling, 3195T, Lot # 04/2014, 1:200). The secondary antibodies used were: Donkey anti-rabbit secondary antibody (Clone- Poly4064, Biolegend, 406410, Lot # B243995, 1:200), Goat anti-mouse secondary antibody (Invitrogen, A-21235, Lot # 1939631, 1:500), Goat anti-rat secondary antibody (Invitrogen, A-21247, Lot # 1921562, 1:200) and Donkey anti-rabbit secondary antibody (Clone- Poly4064, BioLegend, 406416, Lot # B243796, 1:200).

For immunoblotting: Primary antibodies were administered at manufacturer recommended concentration: anti-PC1 (7e12, Santa Cruz Biotechnology, sc-130554, 1:500) directed against the LRR, mouse anti-β-actin from (Sigma-Aldrich, A5316, 1:10,000), rat monoclonal E8 antibody raised against PC1-CTF (1:1000), Rabbit polyclonal 3374 antibody raised against C-terminal tail of PC2 (1:1000), provided by the NIH NIDDK sponsored Baltimore Polycystic Kidney Disease Research and Clinical Core Center, P30DK090868 (Dr. Feng Qian).
For mouse inducible pkd2 cells, primary antibody(1:1000 rabbit PC2; Maryland PKD Research and Clinical Core Center #3374) and secondary antibody goat anti-rabbit HRP; Jackson ImmunoResearch Laboratories 111035144, 1:5000 were used.

Validation

Antibodies were validated by each manufacturer prior to purchasing following rigorous validation protocol. Both E8 (PC1) and 3374 (PC2) were validated using rigorous, genetically defined positive and negative controls. Tagged recombinant proteins were expressed from a full-length cDNA expression constructs in mammalian cells. This allowed for detection of the proper cleavage pattern and modifications of the proteins. These were used as positive controls. Protein null tissues or cells were used to control for the specific detection of any protein products. Thus providing negative controls. The correct identification of proteins in the positive samples was demonstrated by the lack of the signals in null samples.

Eukaryotic cell lines

Policy information about cell lines

Cell line source(s)	MDCK-II and NIH 3T3 cell lines were obtained from ATCC.<br><br>Human cells primary cells:<br>NHKc [Primary Wild type cortical renal epithelial cells]<br>NHKm [Primary Wild type medullary renal epithelial cells]<br>ADPKD [Primary ADPKD renal epithelial cells]<br>Source: Primary human normal (cortex and medulla) and ADPKD (cortical cyst wall) renal epithelial cells were received from the Cell Culture and Engineering Core of the Maryland Polycystic Kidney Disease Research and Clinical Core Center (collection and usage of human material under the approval of the University of Maryland, Baltimore Internal Review Board).<br><br>Mouse PKd2 inducible cell lines:<br>Pax8rtTA, TetOCre, Pkd2cond/cond Immortalized clone # 125<br>Source: Maryland PKD Research and Clinical Core Center, Cell Culture and Engineering Core (CCEC), part of the NIIH U54 PKD Research Resource Consortium (PKD-RRC). Epithelial cell lines generated from Pax8 Tet-o-CrePkd2 fl/fl mice crossed with the immorto-mouse expressing the temperature sensitive SD40 large T-antigen.
Authentication	MDCK II cells were authenticated by ATCC. We did not perform independent authentication.<br><br>For human primary cells, their origin and epithelial nature were authenticated by visual inspection and marker presence by the Cell Culture and Cell Engineering Core of the Maryland PKD Research and Clinical Core Center before distribution. Similar resources are available from the PKD-RRC: https://www.pkd-rrc.org.<br><br>For mouse Pkd2 inducible cell lines, their origin (inner medulla) and epithelial nature were authenticated by visual inspection, marker presence, and by single cell RNAseq by the CCEC before distribution. The same cells are available to all the academics studying ADPKD through the PKD-RRC.
Mycoplasma contamination	MDCK-II, NIH 3T3 and human cells were tested negative for mycoplasma contamination.<br>For mouse cells, all immortalized cell lines from the CCEC are periodically tested for mycoplasma. The cells tested negative.
Commonly misidentified lines<br>(See ICLAC register)	None.
