# Supplementary material for: Kidney epithelial cells are active mechano-biological fluid pumps
Source: Nat Commun. 2022 Apr 28;13:2317. doi: 10.1038/s41467-022-29988-w (PMC9050750; doi:10.1038/s41467-022-29988-w)
Supplement: Supplementary file 3 — Description of Additional Supplementary Files [file 41467_2022_29988_MOESM3_ESM.pdf]

Description of Additional Supplementary Files

Supplementary Movie 1: Time-lapsed videography of the fluid pumping action of MDCK-II cells in MFKP and corresponding pump performance curve.
Time lapsed videography of the fluid pumping action of MDCK-II epithelium grown in MFKP. Plot representing the variation of fluid height in the MC with time. The pump performance curve of NHKc epithelium indicating the variation of trans-epithelial fluid flux (J) with the hydrostatic pressure gradient (ΔP) developed across the epithelium. Time step is 1 min.

Supplementary Movie 2: Time-lapsed videography of the fluid pumping action of NHKc, NHKm and ADPKD cystic cells in MFKP and corresponding pump performance curve.
Time lapsed videography of the fluid pumping action of NHKc, NHKm and ADPKD epithelium grown in MFKP. Variation of fluid height in the MC with time for NHKc, NHKm and ADPKD cells. The pump performance curve of NHKc, NHKm and ADPKD cells indicating the variation of transepithelial fluid flux (J) with the hydrostatic pressure gradient (ΔP) developed across the epithelium. Time step is 1 min.

Supplementary Movie 3: Na^+/K^+ ATPase dynamics under hydraulic pressure gradient (ΔP).
Live imaging of SNAP-tagged Na+/K+ ATPase (NKA) in MDCK-II epithelium grown in MFKP under a hydraulic pressure gradient (ΔP). Confocal imaging of the baso-lateral section a field of view in the epithelium is shown under three conditions: No pressure gradient (ΔP = 0), pressure gradient applied (ΔP = 200 Pa) and pressure gradient removed (ΔP = 0). Red arrowheads indicate location of NKA disruption. Scale bar = 10 µm.

Supplementary Movie 4: F-actin dynamics under hydraulic pressure gradient (ΔP)
Live imaging of GFP-tagged F-actin (F-tractin) in MDCK-II epithelium grown in MFKP under hydraulic pressure gradient (ΔP). Confocal imaging of the baso-lateral section a field of view in the epithelium is shown under three conditions: No pressure gradient (ΔP = 0), pressure gradient applied
(ΔP = 200 Pa) and pressure gradient removed (ΔP = 0). Scale bar = 10 µm.

Supplementary Movie 5: Fluctuating epithelial dome
Differential interference contrast (DIC) time-lapsed video of an unstable fluctuating MDCK-II domes on glass substrate. Original time step is 30 mins. Video was saved at 5 frames per second. scale bar = 50 µm

Supplementary Movie 6: Mature epithelial dome
Differential interference contrast (DIC) time-lapsed video of stable MDCK-II domes on glass substrate. Original time step is 30 mins. Video was saved at 5 frames per second. scale bar = 50 µm.
